# Author Correction: CBP-HSF2 structural and functional interplay in Rubinstein-Taybi neurodevelopmental disorder

**DOI:** 10.1038/s41467-023-41869-4

**Published:** 2023-09-28

**Authors:** Aurélie de Thonel, Johanna K. Ahlskog, Kevin Daupin, Véronique Dubreuil, Jérémy Berthelet, Carole Chaput, Geoffrey Pires, Camille Leonetti, Ryma Abane, Lluís Cordón Barris, Isabelle Leray, Anna L. Aalto, Sarah Naceri, Marine Cordonnier, Carène Benasolo, Matthieu Sanial, Agathe Duchateau, Anniina Vihervaara, Mikael C. Puustinen, Federico Miozzo, Patricia Fergelot, Élise Lebigot, Alain Verloes, Pierre Gressens, Didier Lacombe, Jessica Gobbo, Carmen Garrido, Sandy D. Westerheide, Laurent David, Michel Petitjean, Olivier Taboureau, Fernando Rodrigues-Lima, Sandrine Passemard, Délara Sabéran-Djoneidi, Laurent Nguyen, Madeline Lancaster, Lea Sistonen, Valérie Mezger

**Affiliations:** 1 Université de Paris, CNRS, Epigenetics and Cell Fate, F-75013 Paris, France; 2 Faculty of Science and Engineering, Cell Biology, Åbo Akademi University, Turku, Finland; 3 Turku Bioscience Centre, University of Turku and Åbo Akademi University, Turku, Finland; 4 Université de Paris, CNRS, Unité de Biologie Fonctionnelle et Adaptative, Paris, France; 5 Ksilink, Strasbourg, France; 6 Laboratory of Molecular Regulation of Neurogenesis, GIGA-Stem Cells and GIGA-Neurosciences, Interdisciplinary Cluster for Applied Genoproteomics (GIGA-R), University of Liège, CHU Sart Tilman, Liège, Belgium; 7 Université de Nantes, CHU Nantes, Inserm, CNRS, SFR Santé, Inserm UMS 016, CNRS UMS 3556, F-44000 Nantes, France; 8 INSERM, UMR1231, Laboratoire d’Excellence LipSTIC, Dijon, France; 9 University of Bourgogne Franche-Comté, Dijon, France; 10 Département d’Oncologie médicale, Centre Georges-François Leclerc, Dijon, France; 11 CNRS, UMR 7592 Institut Jacques Monod, F-75205 Paris, France; 12 Department of Medical Genetics, University Hospital of Bordeaux, Bordeaux, France and INSERM U1211, University of Bordeaux, Bordeaux, France; 13 Service de Biochimie-pharmaco-toxicologie, Hôpital Bicêtre, Hopitaux Universitaires Paris-Sud, 94270 Le Kremlin Bicêtre, Paris-Sud, France; 14 Université de Paris, INSERM, NeuroDiderot, Robert-Debré Hospital, F-75019 Paris, France; 15 Genetics Department, AP-HP, Robert-Debré University Hospital, Paris, France; 16 Department of Cell Biology, Microbiology, and Molecular Biology, College of Arts and Sciences, University of South Florida, Tampa, FL USA; 17 MRC Laboratory of Molecular Biology, Cambridge Biomedical, Campus, Cambridge, UK; 18 Present Address: KTH Royal Institute of Technology, Stockholm, Sweden; 19 Present Address: Neuroscience Institute-CNR (IN-CNR), Milan, Italy

**Keywords:** Developmental neurogenesis, Acetylation, Neural stem cells, Developmental disorders

Correction to: *Nature Communications* 10.1038/s41467-022-34476-2, published online 16 November 2022

In this article the affiliation ‘Ksilink, Strasbourg, France’ for Carole Chaput was missing.

In addition, the CIFRE grant 2021/0560, subsidized by the National Association of Research and Technology (ANRT—Association Nationale de la Recherche et de la Technologie, France) and relating to Association Ksilink for Carole Chaput was omitted.

The original article has been corrected.

