# Supplementary material for: CBP-HSF2 structural and functional interplay in Rubinstein-Taybi neurodevelopmental disorder
Source: Nat Commun. 2022 Nov 16;13:7002. doi: 10.1038/s41467-022-34476-2 (PMC9668993; doi:10.1038/s41467-022-34476-2)

SUPPLEMENTARY INFORMATION

de Thonel et al.
“CBP-HSF2 structural and functional interplay in Rubinstein-Taybi neurodevelopmental disorder”

Contains:

Supplementary Figures 1 to 11 (pages 2-24)
Supplementary Tables 1 and 2 (pages 25-27)
Supplementary Methods (pages 28-33)
Supplementary Notes (Supplementary Acknowledgments) (page 34)
Supplementary Discussion (page 35)
Supplementary References (pages 36-39)
Supplementary Figures Source Data (page 40-48)

SUPPLEMENTARY FIGURES

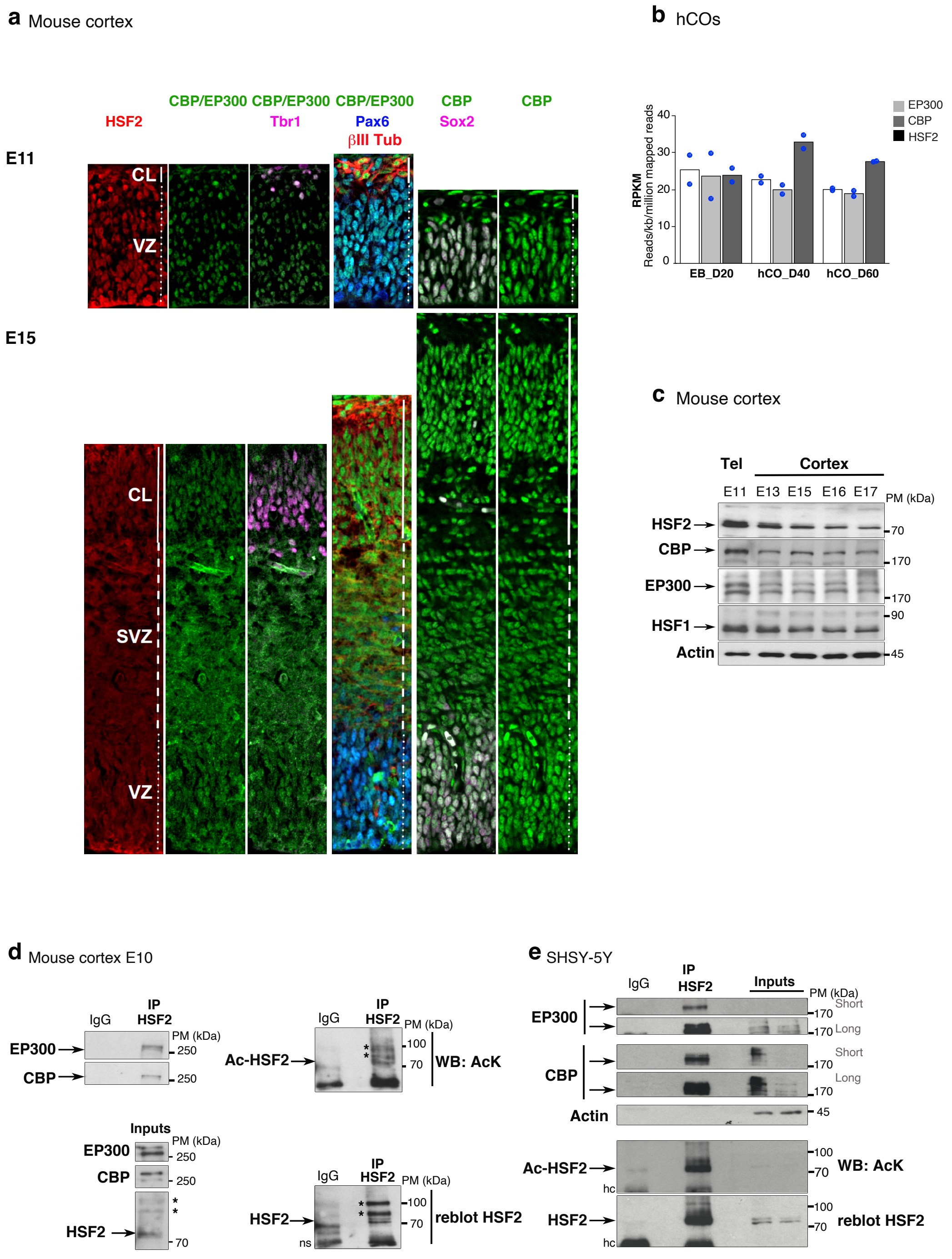

Supplementary Fig. 1 HSF2 is expressed, acetylated, and interacts with EP300/CBP in neurodevelopmental contexts. (related to Fig. 1)

(*Continued next page*)

Supplementary Fig. 1 HSF2 is expressed, acetylated, and interacts with EP300/CBP in neurodevelopmental contexts.

a Co-expression of HSF2 and CBP/EP300 in the E11 and E15 mouse cortex. Representative immunofluorescence of mouse telencephalon at embryonic day 11.5 (E11) or mouse cortex at E15.5 (E15) showing that HSF2 (red) and CBP/EP300 (green) are expressed in neuroprogenitor cells stained by Pax6 (blue) or Sox2 (purple) and in neurons stained by Tbr1 (purple) or class III β-tubulin (βIII tub, red). Bottom, apical side; top, basal side. VZ, ventricular zone; SVZ, subventricular zone; CL, cortical layer. Each panel is 70 µm wide.

b HSF2, EP300, and CBP (CREBBP) mRNAs are expressed along the differentiation process of human brain organoids. Graph comparing mRNAs amounts, expressed as RPKM (reads/kb/million mapped reads) (n = 2 independent data sets, for each stage from the RNA-Seq data GSE82022). Error bars, mean +/- standard deviation (s.d.).

c HSF2, CBP, EP300 proteins are expressed at all stages of mouse cortical development.
Representative immunoblots of protein extracts from E11 telencephalon (Tel) and E13 to E17 mouse cortex showing the expression profiles of HSF2, HSF1, CBP and EP300, in line with previously reported expression patterns[1–9] (n = 2 independent experiments).

d HSF2 interacts with EP300 and is present in an acetylated form in the E10 telencephalon. Representative immunoblots of immunoprecipitated HSF2 (IP HSF2) showing co-immunoprecipitation of endogenous EP300 and CBP and acetylation of endogenous HSF2 (Ac-HSF2) (n = 3 independent experiments). *, high molecular weight forms of HSF2 forms typically detected in the mouse cortex[3], possibly corresponding to post-translational modifications, e.g. sumoylation[10]. hc, heavy chain. Inputs, total proteins in input samples.

e HSF2 interacts with EP300 and is present in an acetylated form in the human SHSY-5Y neural cell line. Representative immunoblots of immunoprecipitated HSF2 (IP HSF2) showing co-immunoprecipitation of endogenous EP300 and CBP proteins (n = 3). hc, IgG heavy chain. Inputs, total proteins in input samples. Short and long exposures are shown.

Source data are provided as a Source Data file.

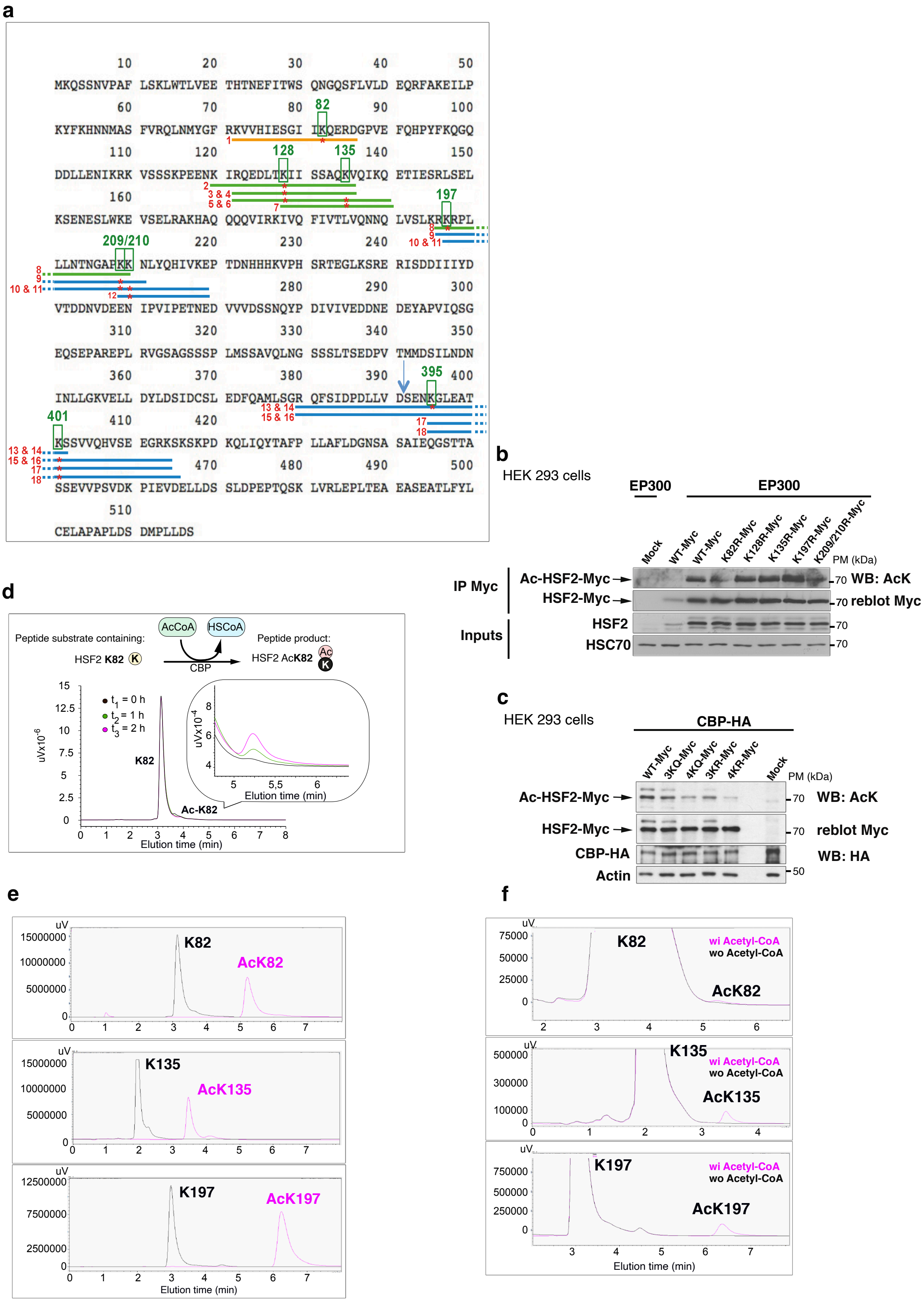

Supplementary Fig. 2 HSF2 is acetylated by CBP and EP300 in normal conditions. (related to Fig. 2).

(*Continued next page*)

Supplementary Fig. 2 HSF2 is acetylated by CBP and EP300 in normal conditions.

a Positioning of the acetylated lysine residues identified by MS in the mHSF2β protein. Amino-acid sequence of mouse HSF2 showing the acetylated lysine (green boxes) and the peptides used for *in vitro* acetylation tests (underlined with different colors corresponding to the different HSF2 domains and numbered in red). The peptides and their sequences are also listed in Supplementary Table 1.

b Impact of the mutations of single (K82, K128, K135, and K197) and doublet (K209/K210) lysine residues on HSF2 global acetylation by EP300. Immunoblots of immunoprecipitated HSF2-Myc (IP Myc) from HEK 293 cells co-transfected with mock, HSF2-Myc WT or HSF2-Myc mutants (on the indicated lysine residues) and with EP300-HA showing HSF2 acetylation levels (Ac-HSF2). Inputs, total proteins in input samples (n=3 independent experiments).

c Impact of the combined mutations of 3 (K128, K135, and K197) or 4 (K82, K218, K135, and K197) lysine into glutamine (3KQ or 4KQ) or arginine residues (3KR or 4KR) on HSF2 acetylation by CBP. Representative immunoblots of immunoprecipitated HSF2-Myc (IP Myc) from HEK 293 cells co-transfected with mock or HSF2-Myc WT or HSF2-Myc mutants (on the indicated lysine residues) with CBP-HA showing HSF2 acetylation levels (n = 3 independent experiments).

d Kinetics of *in vitro* acetylation of HSF2 peptides containing the K82 residue by CBP. Time course elution of HSF2K82 peptide detected by RP-UFLC, as in Fig. 2e. Note that we could not perform this experiment on the HSF2 peptide containing K128, due to its insolubility (Manufacturer's information). See Methods for HSF2K82 peptide sequence.

e Control experiments for the determination of the elution profiles of the HSF2 peptides K82 and acetylated K82 (AcK82), K135 and AcK135, and K197 and AcK197 peptides, using non-acetylated and synthetically acetylated commercial peptides. Separation was monitored by RP-UFLC.

f Control experiments for Acetyl-CoA-dependent acetylation of HSF2 K82, K135 and K197 peptides in the presence of CBP Full-HAT. HSF2 peptide substrates were incubated in the presence of recombinant purified CBP Full HAT and with or without acetyl-CoA for 20 minutes. As in (**e**).

Source data are provided as a Source Data file.

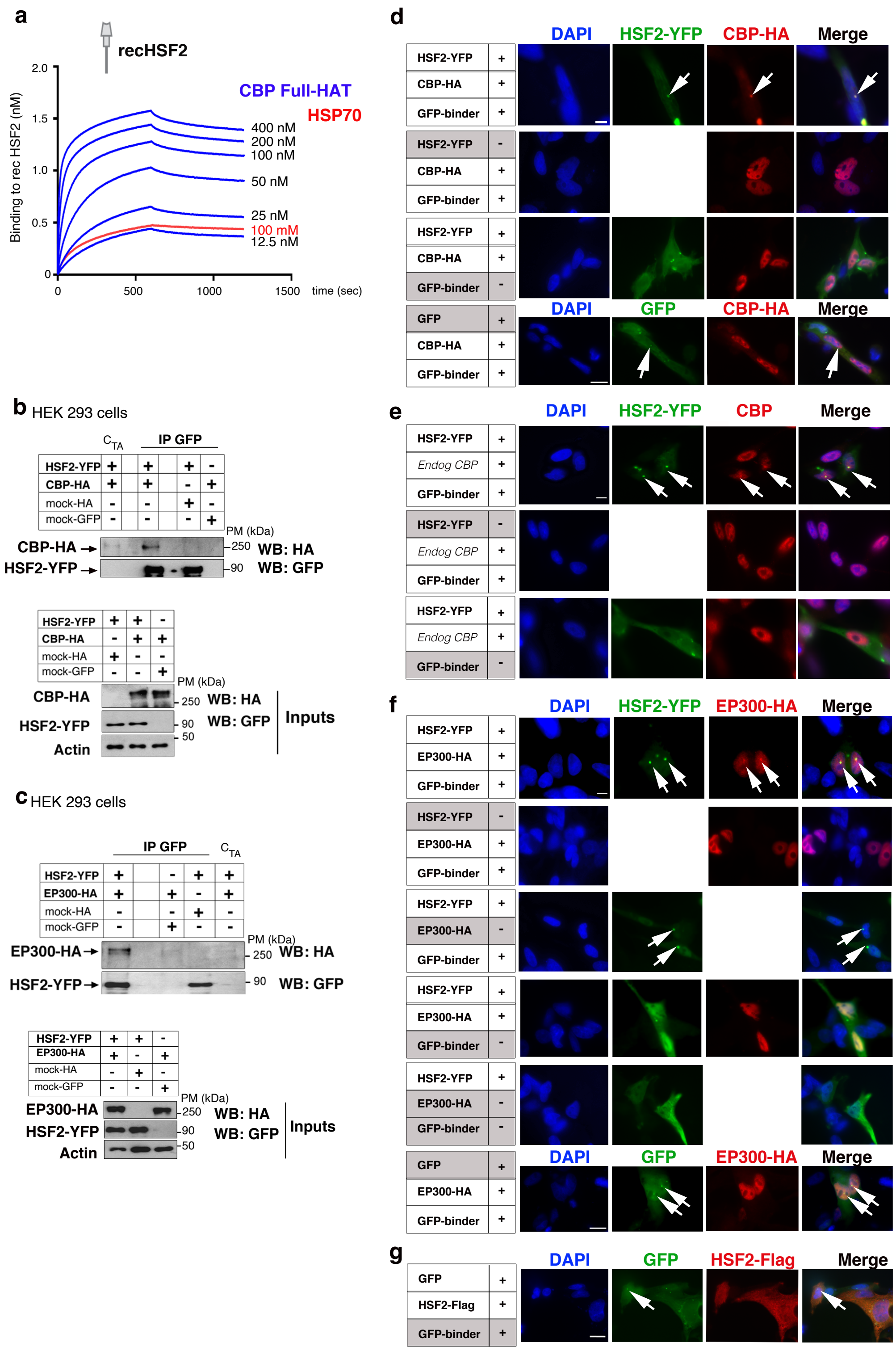

Supplementary Fig. 3 HSF2 interacts with CBP and EP300 in normal conditions. (related to Fig. 3).

(***Continued next page***)

Supplementary Fig. 3 HSF2 interacts with CBP and EP300 in normal conditions.
a *Determination of the Kd of CBP Full-HAT domain affinity to HSF2.* Biolayer interferometry to determine association and dissociation curves of CBP Full-HAT domain (concentration range from 12.5 µM to 400 nM) with biotinylated HSF2 immobilized on streptavidin sensor tips (blue curves). As a positive control the binding profile of HSP70 (100 nM) is plotted (red curve). The interaction of HSF2 with CBP Full HAT or CBP PHD domain is more efficient than with HSP70, which has been reported to interact with HSF2 (Fig. 3a; [11,12]).
b *Ectopically expressed YFP-HSF2 protein interacts with exogenous HA-CBP.* Representative immunoblots of immunoprecipitated HSF2-YFP (IP GFP) from HEK 293 cells transfected with combinations of HSF2-YFP, CBP-HA, mock-HA or mock-GFP showing co-immunoprecipitation of CBP with HSF2 (n = 3). C_{TA}, Trap®-A beads used as a negative control. Inputs, total proteins in input samples.
c *Ectopically expressed YFP-HSF2 protein interacts with exogenous EP3*00. Representative immunoblots of immunoprecipitated HSF2-YFP (IP GFP) from HEK 293 cells transfected with combinations of HSF2-YFP, EP300-HA, mock-HA or mock-GFP showing co-immunoprecipitation of EP300 with HSF2 (n = 3). C_{TA}, Trap®-A beads used as a negative control.
d-g *F3H control experiments of the visualization of interaction between HSF2-YFP and exogenous CBP-HA, endogenous CBP and exogenous EP300*. Representative confocal sections of BHK cells carrying a stably integrated Lac-operator array transfected with CBP-HA (**d**) or EP300-HA (**f**) or none (**e, g**) with either HSF2-YFP, GFP or none and with or without LacI-GFP binder (n = 3 independent experiments). White arrows, nuclear *lacO* array identified with GFP recruitment through the GFP binder. Scale bar: 10 µm. In absence of GFP-binder, HSF2-YFP is not recruited to the *lacO* array, as well as CBP-HA (**d**), endogenous CBP (**e**), EP300-HA (**f**). Note that in the case of absence of transfection with HSF2-YFP or EP300-HA, no images were taken at the corresponding wavelength (white squares).
d CBP-HA is recruited to the *Lac0* array only in presence of HSF2-YFP, but not in its absence or with GFP. Note that in absence of GFP-binder, HSF2-YFP is able to aggregate in the cytosol but fails to recruit CBP-HA.
e endogenous CBP is recruited to the *Lac0* array only in presence of HSF2-YFP, but not in its absence.
f EP300-HA is recruited to the *Lac0* array only in presence of HSF2-YFP, but not in its absence or with GFP.
g Flagged-HSF2 failed to be recruited at lacO array.
Source data are provided as a Source Data file.

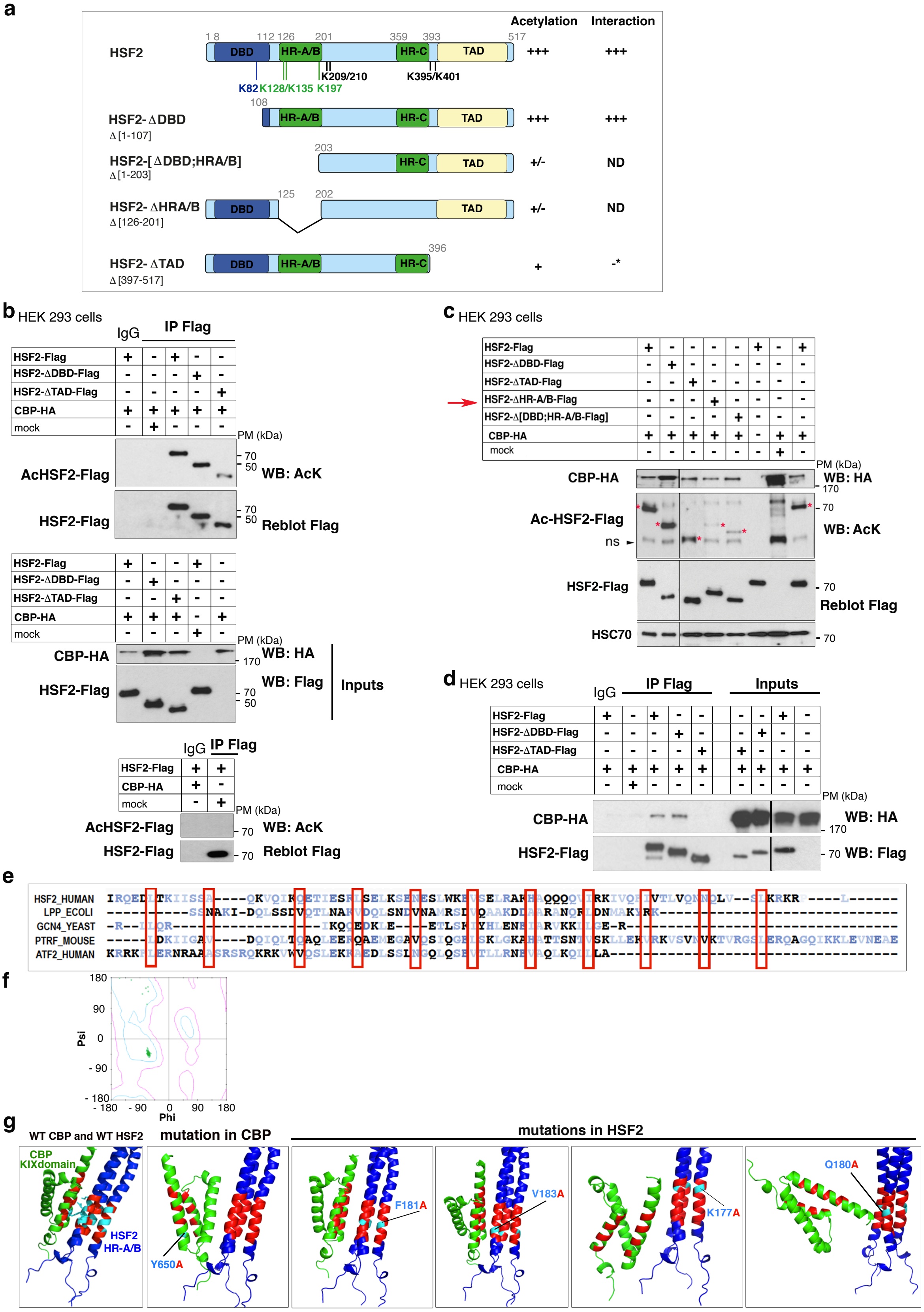

Supplementary Fig. 4 Identification of the HSF2 domains that interact with CBP. (related to Fig. 4).

(Continued next page)

Supplementary Fig. 4 Identification of the HSF2 domains that interact with CBP.
a ***Qualitative summary of the impact of the deletion of different functional domains of Flag-HSF2 on its acetylation status and interaction with CBP-HA***, corresponding to experimental data shown in (**b-d**). +++, strong; + moderate; +/- low acetylation or interaction. ND not detectable, in that case, the Flag tag is not recognized by the antibody, likely because it is masked by the aberrant conformation of the truncated HSF2 protein. -*, not observed, the interaction might be very labile in the absence of the TAD domain, a typical docking site for CBP in many transcription factors.
b-d ***Determination of the HSF2 domains necessary for HSF2 acetylation by CBP-HA and interaction with CBP.*** HEK 293 cells were transfected with CBP-HA and the WT HSF2-Flag or HSF2 deleted forms (as indicated and represented in **a**) (n = 3).
b ***Representative immunoblots of immunoprecipitated HSF2-Flag*** (IP Flag) showing HSF2 acetylation status (WB: AcK) and the expression levels of the different HSF2 constructs (WB: Flag). Inputs, total proteins in input samples. Immunoprecipitated HSF2-Flag (IP Flag) is not acetylated in absence of CBP-HA (negative control).
c ***Representative immunoblots of HSF2 acetylation levels*** by CBP-HA comparing WT Flag-HSF2 with the HSF2 deleted forms described in (**a**). CBP-HA and HSF2-Flag expression levels are shown. * Acetylated forms of the WT and deleted HSF2-Flag. HSC70, loading control.
d ***Representative immunoblots of immunoprecipitated HSF2-Flag*** (IP Flag) showing co-immunoprecipitation of CBP-HA with WT or deleted HSF2-Flag and the levels of the different HSF2 constructs. Inputs, total proteins in input samples (n=3 independent experiments).
e ***Sequence alignment of HR-A/B domains used for in silico modeling*** between human HSF2 HR-A/B (from aa. 121 to 201), lipoprotein Lpp56 of *E. coli*, yeast transcription factor GCN4 (mutated on some residues to generate stabilized heptad repeats), murine PTRF and human ATF2 transcription factors[13,14]. Alignment was done with Discovery Studio and Clustal W multiple sequence alignment program. Red box, critical aa in the heptad repeat.
f ***Ramachandran plot*** (*Discovery studio*) showing the good quality of the triple coiled-coil model structure of the HSF2 HR-A/B determined by *in silico* modeling (Fig. 4b) and based on sequence similarity with protein HR-A/B aligned in (**e**).
g In silico *analysis of the impact of the single mutations Y650A in the CBP KIX domain, F181, V183, K177, and Q180A in the HSF2 KIX recognition motif on CBP – HSF2 interactions*. The mutations Y650A, K177A and Q180A hamper the interaction between the HRA/B domain and KIX domain, while F181A and V183A have no effect (*Firedock* analysis; Supplementary Table 3).
Source data are provided as a Source Data file.

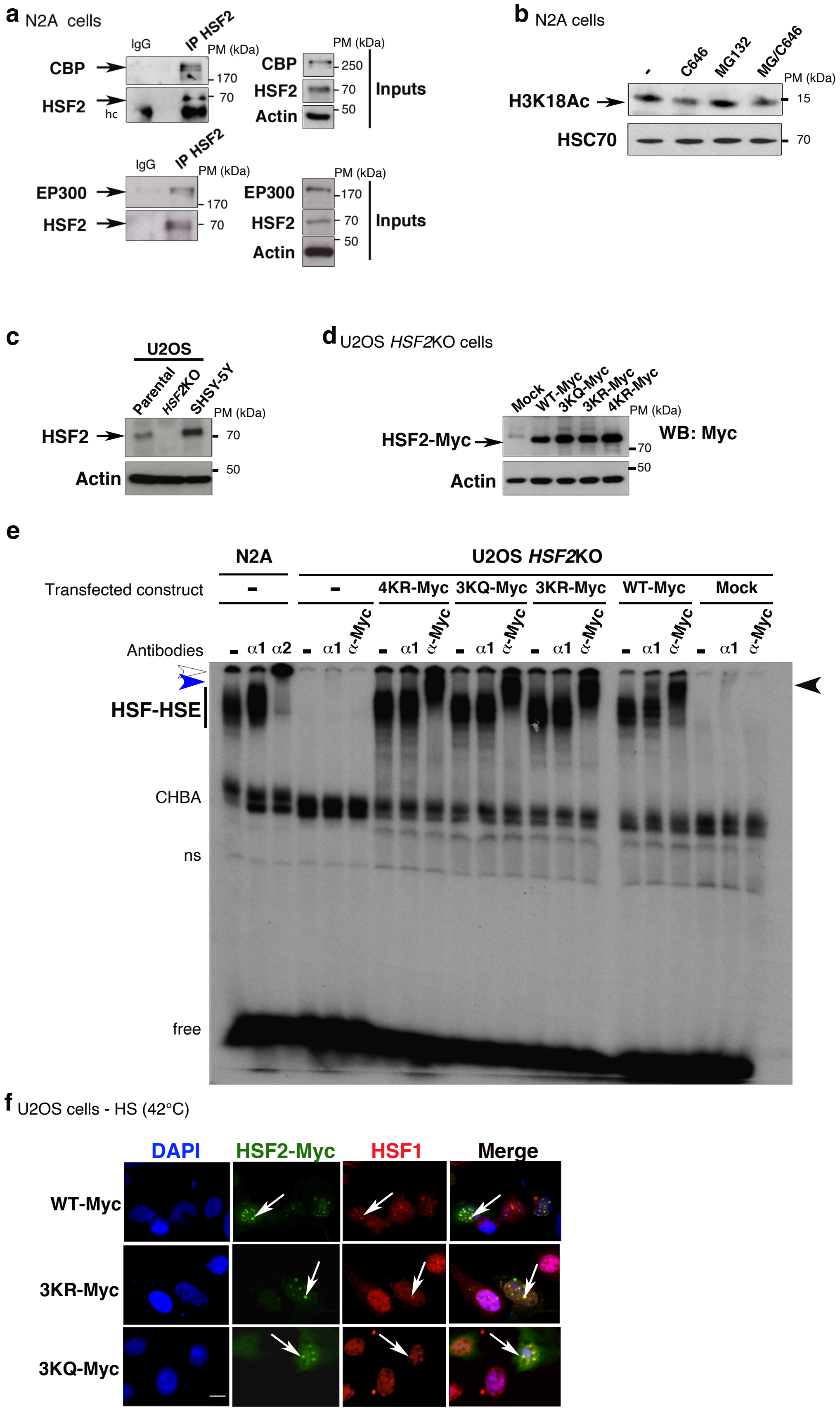

Supplementary Fig. 5. HSF2 interaction with and acetylation by CBP/EP300 in the N2a neural cell line and validation of HSF2 mutants. (related to Fig. 5).

(Continued next page)

Supplementary Fig. 5 HSF2 interaction with and acetylation by CBP/EP300 in the N2a neural cell line and validation of HSF2 mutants.

a *Interaction between endogenous HSF2 and CBP/EP300 proteins in neural cells.* Representative immunoblots of HSF2 immunoprecipitated from N2a cells showing co-immunoprecipitation of CBP or EP300 proteins (n = 3). Inputs, total proteins in input samples. Actin, loading control. hc: IgG heavy chain.

b *Decreased acetylation levels of lysine residue K18 of histone H3 (H3K18Ac) assess C646 efficiency in inhibiting CBP/EP300 activity*. Representative immunoblots of protein extracts from N2a cells treated with the CBP/EP300 inhibitor C646 (40 µM for 4 h) and/or with MG132 (20 µM for 6h), showing the impact of C646 on lysine acetylation. HSC70, loading control. Relative to Fig. 5a (n=2 independent experiments).

c *Representative immunoblot analysis of HSF2 content in the CRISPR/Cas9 HSF2*KO *cells*. Representative immunoblots of protein extracts from parental and *HSF2*KO U2OS cells, and SHSY-5Y cells showing respective endogenous HSF2 levels. SHSY-5Y were loaded as positive controls for the detection of HSF2. Actin, loading control. See [15] for the detailed description of the *CRISPR/Cas9 HSF2*KO cells (n=3 independent experiments).

d *Myc-HSF2WT, Myc-HSF2 3KQ, Myc-HSF2 3KR, and Myc-HSF2 4KR are ectopically expressed at similar levels in U2OS* HSF2*KO cells*. Representative immunoblots of protein extracts from cells (transiently transfected cells) (n=3 independent experiments). Actin, loading control.

e *Ectopically expressed Myc-HSF2WT, and mutant Myc-HSF2 3KQ, Myc-HSF2 3KR, and Myc-HSF2 4KR exhibit HSF2 DNA-binding activity* ex vivo. Gel-shift analysis of HSF1 and HSF2 DNA-binding activity in U2IS *HSF2*KO cells, expressing Myc-tagged HSF2WT, HSF2 3KQ, HSF2 3KR, or HSF2 4KR, (or Myc (mock) as a negative control). The presence of HSF1 and/or HSF2 in the HSF-HSE complex was analyzed by supershifting (arrowheads) with anti-HSF1 (α1; black arrowhead) or anti-HSF2 (α2; white arrowhead), or anti-Myc antibodies (α-Myc; blue arrowhead). N2A cells were loaded as positive controls for anti-HSF2 antibodies. Notably, *HSF2*KO cells expressing HSF2WT, HSF2 3KQ, HSF2 3KR, and HSF2 4KR proteins allow the formation of an HSE-HSF complex, which is mainly supershifted by anti-HSF2 (but almost not by anti-HSF1), whereas, as expected, mocked transfected *HSF2*KO cells are devoid of HSF2 activity. HSF-HSE: HSF-HSE complexes. CHBA: constitutive HSE-binding activity, which is not carried by HSFs[16,17]; NS: non-specific DNA-protein complex; free: unbound double-stranded HSE oligonucleotide (n = 2 independent experiments).

f *Myc-HSF2WT, HSF2 3KQ, or HSF2 3KR proteins localize into nuclear-stress bodies upon HS.* Representative immunofluorescence of U2OS cells overexpressing HSF2-Myc WT, 3KR and 3KQ in response to heat shock condition (1 h at 42°C), showing the ability of the HSF2 WT and HSF2 mutant proteins (Myc, green) to localize to nSBs (HSF1 nuclear speckles, red) upon HS (n = 2 independent experiments). Scale bar, 20 µm.

Source data are provided as a Source Data file.

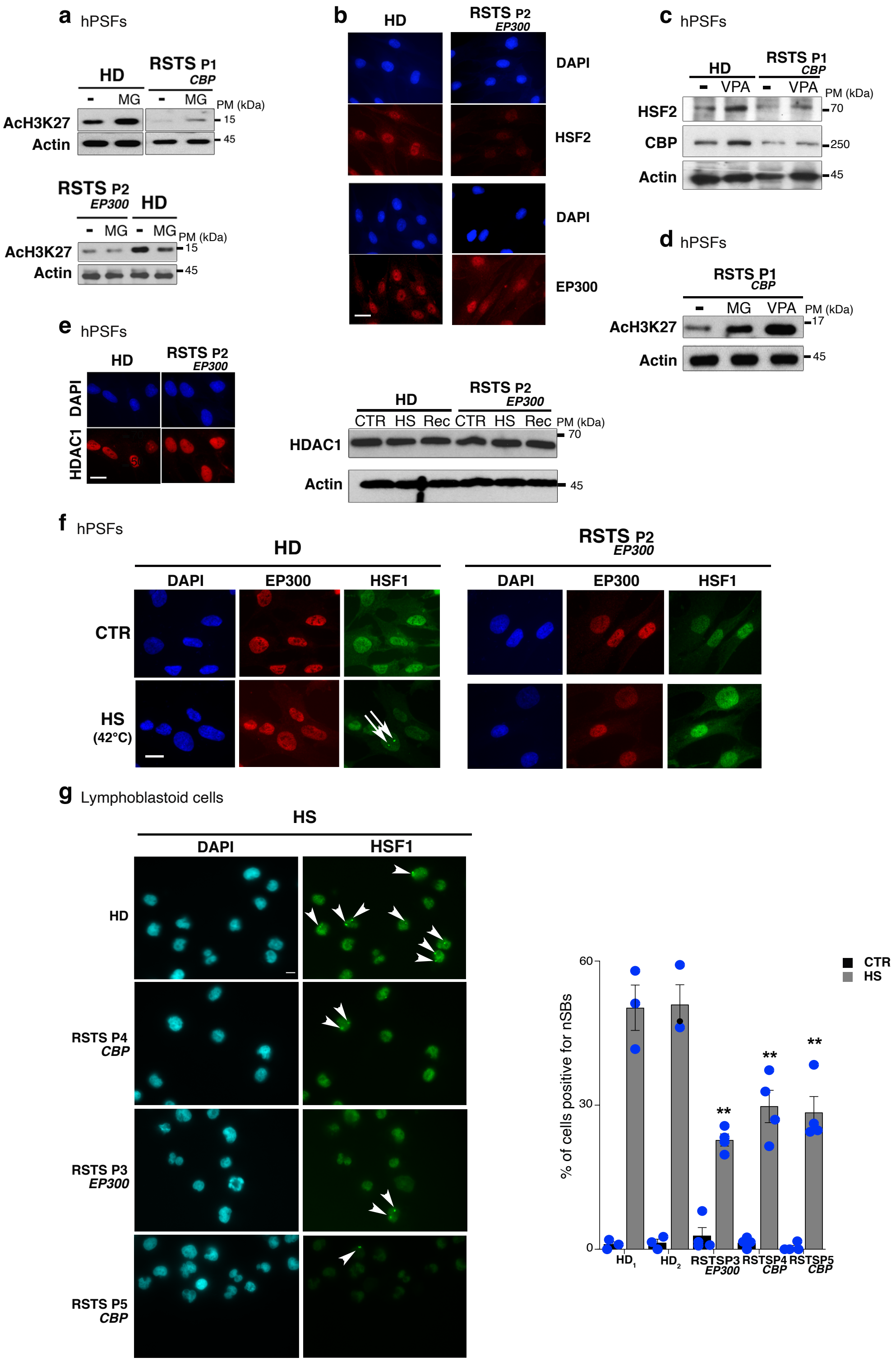

Supplementary Fig. 6 Altered HSF2 protein levels and dysregulation of the stress response in RSTS. (related to Fig. 6).

(*Continued next page*)

Supplementary Fig. 6 Altered HSF2 protein levels and dysregulation of the stress response in RSTS.
a ***Reduction of H3K27 acetylation (AcH3K27) in hPSFs from RSTS patients compared to HD***. Representative immunoblots of protein extracts from HD, RSTS $P1_{CBP}$, and RSTS $P2_{EP300}$ hPSFs treated with MG132 (MG, 20 µM, 6 h) (n = 2).
b ***HSF2 staining is reduced in $RSTS_{EP300}$ hPSFs.*** Representative immunofluorescence of HD and RSTS $P2_{EP300}$ (n = 3). Scale bar, 10 µm.
c ***VPA does not increase HSF2 levels in RSTS $P1_{CBP}$ and $P2_{EP300}$ hPSFs*** (related to Fig. 6b and c), although these cells contain similar levels of HDAC1 (compare to **e**). Representative immunoblot of protein extracts from HD and RSTS $P1_{CBP}$ hPSFs treated with VPA (1 mM, 3 h) (n = 2 independent experiments).
d ***Assessment of the efficiency of VPA in RSTS $P1_{CBP}$ hPSFs***, in increasing in H3K27 acetylation (Related to Fig. 6b). Representative immunoblots of protein extracts from RSTS $P1_{CBP}$ hPSFs treated with VPA (1 mM, 3h), MG132 (20µM, 6h) or vehicle (-) showing increased acetylation of H3K27 by VPA and MG132 (n = 2 independent experiments).
e ***HD and RSTS $P2_{EP300}$ hPSFs contain similar levels of HDAC1***. Immunofluorescence of HD and RSTS $P2_{EP300}$ (n = 1). Immunoblots of protein extracts from HD and RSTS $P2_{EP300}$. (n = 2).
f ***Formation of nSBs, with HSF1 and EP300 expression in HD and $RSTS_{EP300}$ hPSFs, upon HS*** (related to Fig. 6d, e)**.** Representative immunofluorescence of HD and RSTS $P2_{EP300}$ hPSFs in control (CTR) or HS conditions (1 hour at 42°C) (n = 3 independent experiments). Scale bar: 10 µm.
g ***Altered formation of nSBs by HS in $RSTS_{EP300}$ or $RSTS_{CBP}$ lymphoblastoid cells.*** Representative immunofluorescence of HD and RSTS $P4_{CBP}$ cells in HS conditions (1 hour at 43°C). Scale bar, 10 µM. Quantification of the percentage of cells containing nSBs (n = 3, 100 – 150 cells). Error bars, mean +/- SEM; p = 0.0095; **, p < 0.01.
Significance was calculated by two-sided Mann-Whitney test in panel g.
NB: Class I HDAC inhibition by VPA could not restore the HSF2 levels in $RSTS_{CBP}$ or $RSTS_{EP300}$ hPSFs, although HD and RSTS cells displayed similar levels of HDAC1 (Supplementary Fig. 6c-e)
Source data are provided as a Source Data file.

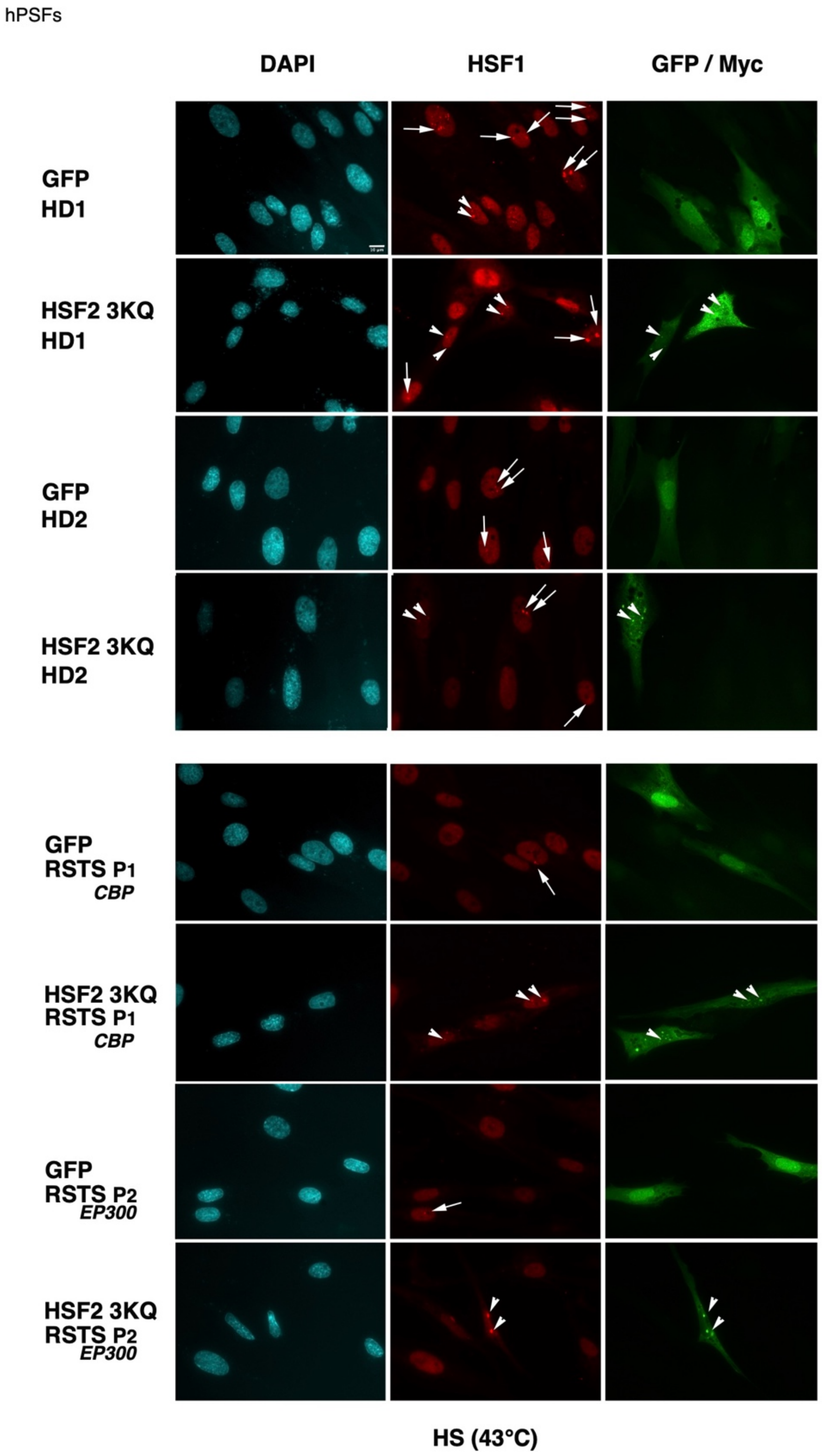

Supplementary Fig. 7 Representative immunofluorescence corresponding to the rescue experiments of HSF2 levels by introduction of HSF2 3KQ that restores the ability to induce nuclear stress bodies (nSBs) in RSTS cells. (related to Fig. 7a)

Supplementary Fig. 7 Representative immunofluorescence corresponding to the rescue experiments of HSF2 levels by introduction of HSF2 3KQ that restores the ability to induce nuclear stress bodies (nSBs) in RSTS cells.
Representative immunofluorescence of HD1, HD2, RSTS P1*CBP* and RSTS P2*EP300* hPSFs, transfected with HSF2 3KQ-Myc or GFP constructs, upon heat shock conditions (43°C for 1 h). Arrows and arrowheads respectively point to nSBs (HSF1-positive nuclear dots, red) located into non-transfected cells or transfected cells, the latter being identified by their expression of either GFP or Myc-HSF2 3KQ (green) (n = 3 independent experiments). Scale bar: 10 µm.
Source data are provided as a Source Data file.

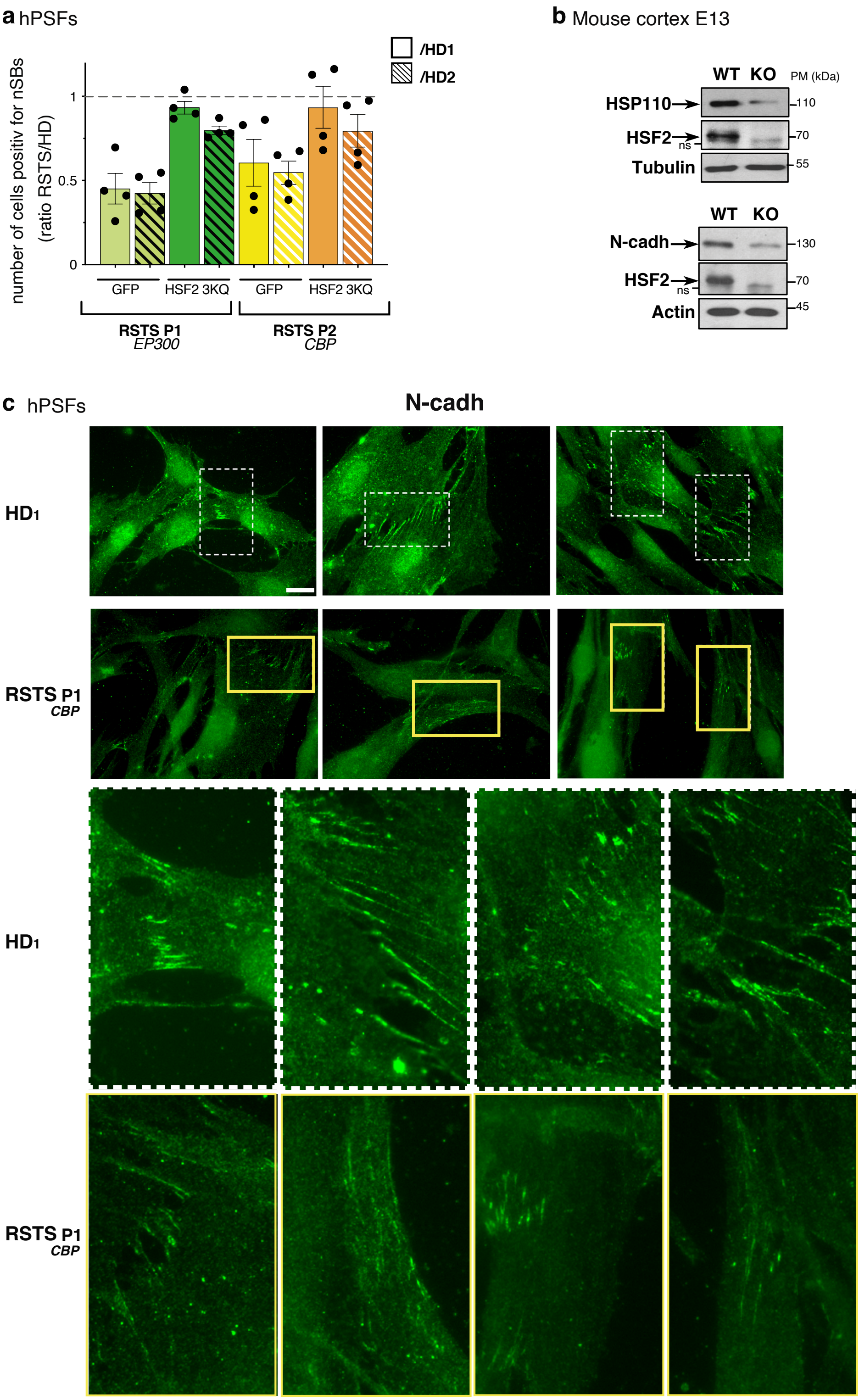

Supplementary Fig. 8 HSF2-dependent dysregulated stress response and neurodevelopmental gene *expression in cells from* RSTS hPSFs. (related to Fig. 7)

(***Continued next page***)

Supplementary Fig. 8 HSF2-dependent dysregulated stress response and neurodevelopmental gene expression in cells from RSTS hPSFs.

a *Rescue of HSF2 levels by introduction of HSF2 3KQ restores the ability to induce nSBs in RSTS cells*. Same data as in Fig. 7a, but different representation: Here, the graph visualizes the average ratios of RSTS P1$_{CBP}$ or P2$_{EP300}$ cells containing nSBs upon HS (1h at 43°C), all normalized either to HD_1 (plain) or HD_2 (dashed) (n = 4). hPSFs were transfected with GFP (control) or HSF2 3KQ to stabilize HSF2. Error bars, mean+/- (SEM).

b *Decreased expression of the HSF2 targets, HSP110 and N-cadherin in mouse* Hsf2*KO cortices at embryonic day E13*. Representative Immunoblots of protein extracts from mouse *Hsf2* wild-type (WT) and *Hsf2*KO E13 brain cortices showing HSP110 or N-cadherin levels (n = 3). Tubulin or actin, loading control.

c *Reduced immunofluorescence of N-cadherin in RSTS hPSFs at cell-cell junctions, compared to HDs*. Representative immunofluorescence of HD1 and RSTS P1$_{CBP}$ hPSFs stained by N-cadherin (green). Yellow and white dotted rectangles, magnified areas showing cell-cell adherent junctions (n = 3 independent experiments). Scale bar: 20 µm.

Source data are provided as a Source Data file.

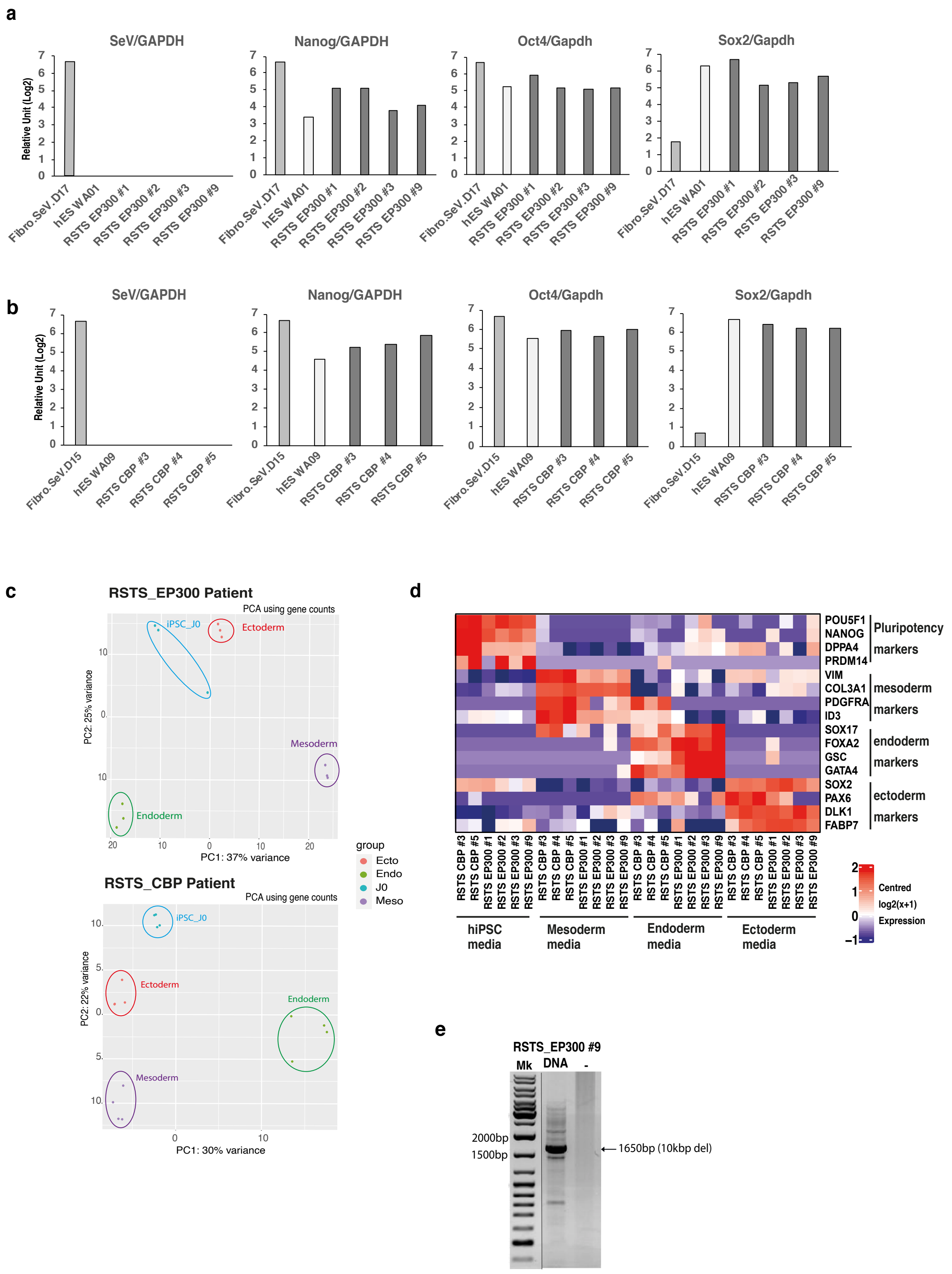

Supplementary Fig. 9 Characterization of the hiPSC clones derived from RSTS P1$_{CBP}$ and RSTS P2$_{EP300}$ patient primary skin fibroblasts. (related to Fig. 7)

(*Continued next page*)

Supplementary Fig. 9 Characterization of the hiPSC clones derived from RSTS P1$_{CBP}$ and RSTS P2$_{EP300}$ patient primary skin fibroblasts.

a-b RT-q-PCR measurement of *SeV*, *OCT4*, *NANOG* and *SOX2* expression in the indicated hiPSC lines.

c Principal component analysis (PCA) plots generated from RNAseq data of RSTS hiPSC and their derivatives after differentiation into endoderm, mesoderm, and ectoderm lineages.

d Heatmap of selected gene expression (RNA-seq data) after differentiation of RSTS hiPSCs into endoderm, mesoderm, and ectoderm lineages, or not (hiPSC) showing their pluripotency.

e Genotyping of RSTS P2$_{EP300}$ iPSCs carrying a 10-11kDa deletion in the *EP300* gene (n = 2 independent experiments).

Source data are provided as a Source Data file.

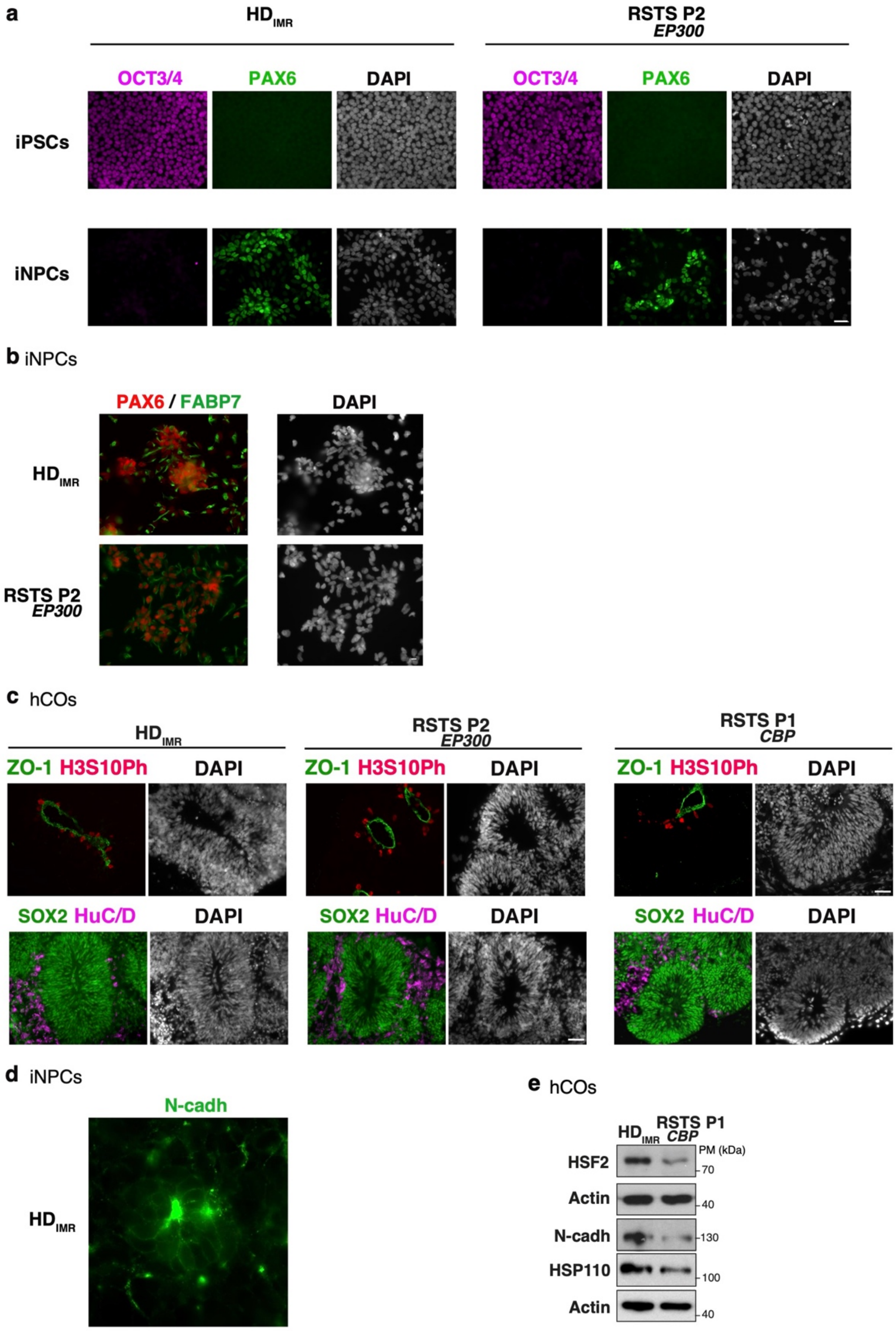

Supplementary Fig. 10 Characterization of iPSCs from the working bank and iPSC-derived NPCs (iNPCs) and of MG132 iNPC treatment. (related to Fig. 7)

(*Continued next page*)

Supplementary Fig. 10 Characterization of iPSCs from the working bank and iPSC-derived NPCs (iNPCs) and of MG132 iNPC treatment.

a Representative Immunofluorescence of HD_{IMR90} and RSTS $P2_{EP300}$ iPSCs and iNPCs (derived from the iPSCs) showing that iPSCs express pluripotency (OCT3/4, purple) but not neuroprogenitor cell markers (PAX6, green), in contrast to NPCs do not express OCT3/4 but PAX6, showing their induction toward the neural lineage (n = 3 independent experiments). Scale bar: 50 µM.

b Representative Immunofluorescence of HD_{IMR90} and RSTS $P2_{EP300}$ iNPCs, showing that they express markers of proliferative neural cells: the marker of neuroprogenitor cells, PAX6 (red), and the radial glia marker FABP7 (green) (n = 3 independent experiments). Scale bar: 50µM.

c Representative Immunofluorescence of HD_{IMR90}, RSTS $P2_{EP300}$ and RSTS $P1_{CBP}$ hCOs showing the ability of HD and RSTS iPSCs to generate stratified cortical-like structures (“loops”) at D25. Neuroprogenitor expressing SOX2 (green) are organized around a ventricle-like cavity delimited by the apical marker ZO-1 (green). Mitotic cells (H3S10Ph, red) are mainly located apically, as seen in neural proliferative areas (VZ). Neurons (HuC/D, magenta) accumulates at the periphery of the proliferative areas resembling to early cortical plates (n = 3 independent experiments). Scale bar, 50µm.

d *N-cadherin signal in HD iNPCs*. Same picture as in Fig. 7f but with higher signal intensity to visualize N-cadherin distribution at the cell membrane and compare it to RSTS iNPCs immunofluorescence in Fig. 7f.

e *Reduced levels of HSF2 and its targets in RSTS $P1_{CBP}$ hCOs, compared with HD hCOs*. Representative immunoblots of proteins extracts from HD_{IMR90} and RSTS $P1_{CBP}$ D25 hCO, comparing HSF2 and its target levels (n = 2 independent experiments).

Source data are provided as a Source Data file.

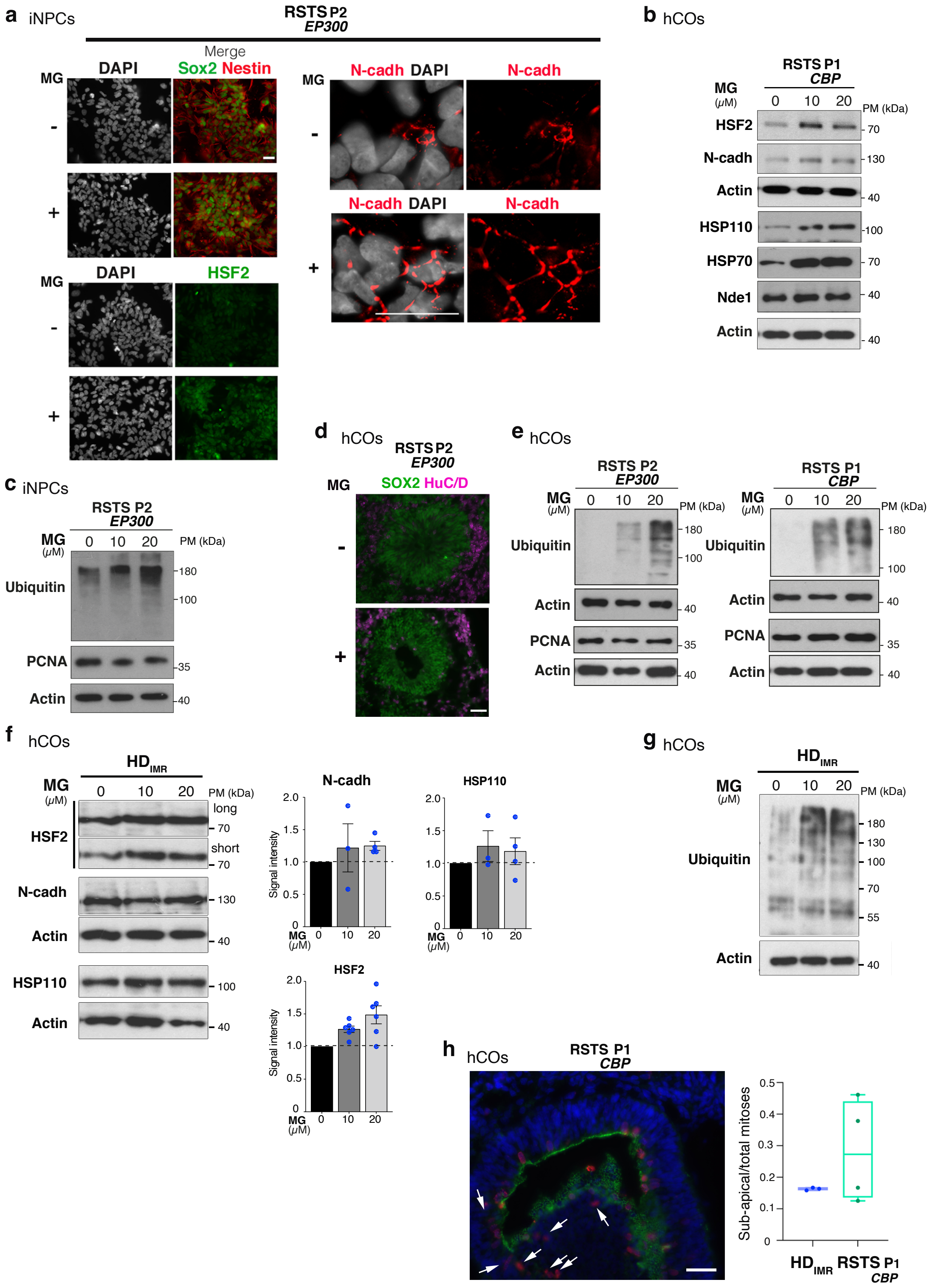

Supplementary Figure 11 RSTS iNPC and hCO characterization upon MG132 treatment. (related to Fig. 8)

(*Continued next page*)

Supplementary Figure 11 RSTS iNPC and hCO characterization upon MG132 treatment.

a The levels of HSF2 and of its targets are restored in the presence of the proteasome inhibitor MG132 in RSTS $_{EP300}$ iNPCs. Representative immunonofluorescence of RSTS P2 $_{EP300}$ iNPCs treated with 20 µM MG132 (+) or vehicle (-) for 8h (n = 3). MG132 treatment does not impair the progenitor characteristics of iNPCs as assessed by immunostaining of SOX2 (green) and Nestin (radial glia-like cells, red). HSF2 (green) signal intensity and N-cadherin (red) distribution are disturbed by MG132 treatment. Scale bars: 50µm.

b ***The levels of HSF2 and of its targets are restored in the presence of the proteasome inhibitor MG132 in RSTS $_{CBP}$ hCOs.*** Representative immunoblots of protein extracts from RSTSP1 $_{CBP}$ hCOs at D24, treated for 8 h with vehicle (0) or MG132 (10 or 20µM), showing HSF2 and its targets levels (n = 3 independent experiments).

c ***Restricted global effects of MG132 on proteome balance upon 8-h treatment in RSTS-derived iNPCs, with no increase in the levels of the known proteasome substrate PCNA.*** Representative immunoblots of protein extracts from RSTS P2 $_{EP300}$ iNPCs after treatment for 8 h with vehicle (0) or MG132 (10 or 20µM) showing Ubiquitin, PCNA and actin levels (n = 3). The levels of PCNA, a known target of the proteasome, are not affected by MG132.

d ***MG132 treatment does not induce major defects in hCO differentiation***. Representative immunofluorescence RSTSP2 $_{EP300}$ D25 hCOs treated with vehicle (-) or MG132 (+) for 16 h, showing neuroprogenitor cells (SOX2, green) and neurons (HuC/D, purple) (n = 3 independent experiments). Scale bar: 50 µm.

e ***Restricted global effects of MG132 on proteome balance upon treatment by MG132, with no increase in the levels of the known proteasome substrate PCNA, in RSTS $_{EP300}$ and RSTS $_{CBP}$ hCOs.*** Representative immunoblots of protein extracts from RSTS P2 $_{EP300}$ and RSTS P1 $_{CBP}$ D25 hCOs after treatment for 8 h with vehicle (0) or MG132 (10 or 20µM) showing ubiquitin, PCNA and actin levels (n = 3).

f ***Treatment of HD hCOs by MG132 does not result in marked increase in the levels of HSF2 or its targets.*** Representative immunoblots of protein extracts from HD $_{IMR90}$ D25 hCOs showing HSF2 and its targets levels after treatment for 8 h with vehicle (0) or MG132 (10 or 20µM). Quantification of signal intensity, normalized to actin (n = 3 independent experiments). Error bars, mean +/- s.d.

g ***Restricted global effects of MG132 on proteome balance in HD hCOs.*** Representative immunoblots of protein extracts from HD $_{IMR90}$ D25 hCOs treated 8 h with vehicle (0) or MG132 (10 or 20µM), showing ubiquitin and actin levels (n = 2 independent experiments).

h ***Ectopic mitoses in RSTS $_{CBP}$ hCOs, compared to HD counterparts, reveal cell-cell adhesion problems***. Representative immunofluorescence images of RSTS P1 $_{CBP}$ D25 hCOs showing the apical belt of VZ-like zones stained for ZO-1 (green) and the apical mitotic progenitor cells (H3S10Ph, red). Scale bar: 50µm. Quantification of the mean sub-apical mitoses, relative to total mitoses per hCO loop (for HD, n = 3 hCOs, 15 loops, 142 mitoses; for RSTS, n = 4 hCOs, 18 loops, 93 total mitoses). The box indicates the upper and lower quartiles and the whiskers indicate the 5th and 95th percentiles of the data.

Source data are provided as a Source Data file.

SUPPLEMENTARY TABLES

#	Sequence<br>(Acetylated lysine in bold)	**Position** of the peptide**/ acetylated lysine**	**Domain of localization of the acetylated K**
1	(K)VVHIESGII**K**QER(D)	72-86/**82**	DBD
2	(K)IRQEDLT**K**IISSAQK(V)	120-136/**128**	HR-AB
3	(R)QEDLT**K**IISSAQK(V)	122-136/**128**	
4	(R)QEDLT**K**IISSAQK(V)	122-136/**128**	
5	(R)QEDLT**K**IISSAQ**K**VQIK(Q)	122-139/**128/135**	
6	(R)QEDLT**K**IISSAQ**K**VQIK(Q)	122-139/**128/135**	
7	(K)IISSAQ**K**VQIK(Q)	128-140/**135**	
8	(R)**K**RPLLLNTNGAPK(K)	196-210/**197**	
9	(R)KRPLLLNTNGAP**K**K(N)	196-210/**209**	Just downstream HR-A/B
10	(K)RPLLLNTNGAP**KK**NLYQHIVK(E)	197-218/**209/210**	
11	(K)RPLLLNTNGAP**KK**NLYQHIVK(E)	197-218/**209/210**	
12	(K)**K**NLYQHIVK(E)	209-219/**210**	
13	(R)QFSIDPDLLVDSEN**K**GLEATK(S)	381-401/**395**	
14	(R)QFSIDPDLLVDSEN**K**GLEATK(S)	381-401/**395**	
15	(R)QFSIDPDLLVDSENKGLEAT**K**SSVVQHVSEEGR(K)	380-415/**401**	
16	(R)QFSIDPDLLVDSENKGLEAT**K**SSVVQHVSEEGR(K)	380-415/**401**	
17	(K)GLEAT**K**SSVVQHVSEEGR(K)	395-414/**401**	Between HR-C and before AD
18	(K)GLEAT**K**SSVVQHVSEEGRK(S)	395-415/**401**	

Supplementary Table 1. Summary of the acetylated peptides found in the mass spectrometry analysis of the mHSF2b isoform. (relative to Figure 2c and Supplementary Figure 2a)
The acetylated lysine residue and its position are highlighted in bold. The peptides are ranked from the N-terminal to C-terminal extremities of the mHSF2**b** protein. Some of the peptides are listed more than one time because they have been found several times in the same M/S experiment. Peptides have been attributed numbers (#), ranked from their N-terminal extremity of the HSF2 protein (relative to Supplementary Figure 2a). Colors correspond to the HSF2 protein domains, schematized in Figure 2c.

Complex	Zdock	Firedock
**Complex HR-A/B_KIX**	**14.66**	**-13.04**
**Mutation Y650A**	14.38	**0.70**
Mutation V183A	14.10	-5.42
Mutation F181A	14.22	-6.16
Mutation Q180A	14.38	-2.36
Mutation K177A	14.16	-8.33

Supplementary Table 2. Docking study between the HR-A/B domain of HSF2 and the KIX domain of CBP/EP300
Docking study - between HR-A/B domain of HSF2 and KIX domain of CBP/EP300 - was processed using *ZDOCK (score) and* FireDock *(energetic score)* and the best *pose* of the complex were sorted in the absence of mutation (belong to the Top 3 for Zdock and Top 1 for Firedock) and in the presence of mutations, within the KIX domain (Y650), or in the HR-A/B domain (V183A, F181A, Q180A, K177A). In red, mutations that impair the interaction between the HR-A/B domain of HSF2 and the KIX domain of CBP/EP300.

SUPPLEMENTARY METHODS

Description of mutations in patients

<u>RSTS hPSFs were isolated from two patients (P1 and P2):</u>

Patient P1- hPSF RSTS*CBP*: Mutation pLys1139X (exon 18) located in the catalytic CBP HAT domain, needed for its acetyltransferase activity. This mutation did not affect CBP or EP300 levels and a marked impact on H3K27 acetylation was observed (Supplementary Fig. 6a).

Patient P2- hPSF RSTS*EP300*: Deletion of at least 10 kb (chr22:41533381.41543435) and to the maximum of 11 kb (chr22:41532396.41543758) confirmed by quantitative multiplex fluorescent (QMF)-PCR. This mutation leads to a truncated protein, with deletion of a binding site, the KIX domain, which is essential for the interaction between HSF2 and EP300/CBP proteins. We show that hPSF exhibit a decreased level in H3K27 acetylation compared to HD as expected in this cell model ([18]; Supplementary Fig. 6a).

<u>Lymphoblastoid cells were derived from three patients (P3-P5):</u>

Patient P3- LB RSTS*EP300*: Mutation pTrp1649X (Exon 30) in *EP300 gene* which gives rise to a stop codon in the HAT domain leading to a C-terminal deletion. This mutation leads to >50% decrease of EP300 protein level with a predicted altered activity (MutPred2).

Patient P4- LB RSTS*CBP*: Deletion in 5' (2 first exons) in *CREBBP gene*. This mutation leads to a truncated protein, which is probably unstable and leads to decreased CBP protein levels.

Patient P5- LB RSTS*CBP*: Mutation p.Arg1498* (exon 27) located in the catalytic CBP HAT domain with formation of a stop codon leading to a C-terminal deletion. This mutation leads to a decrease of CBP protein level.

Generation and characterization of human induced pluripotent stem cell (iPSC) lines

The iPSC lines were generated from RSTS$_{CBP}$ patient [P1]- and RSTS$_{EP300}$ patient patient [P2]-derived primary skin fibroblasts at the iPSC core facility of Nantes University[19-22]. Fibroblasts were reprogrammed by Sendai viruses expressing Oct4, Sox2, Klf4 and c-Myc (CytoTuneTM-IPS 2.0 Sendai Reprogramming kit, Life Technologies). The iPSC clones were picked and expanded on mouse embryonic fibroblasts (MEFs) feeder cells in KSR-FGF2 medium (DMEM/F12 supplemented with 0.1% β-mercaptoethanol, 20% knockout serum replacement, 10 ng/mL basic fibroblast growth factor, 2 mmol/L l-glutamine and 1% NEAA). Until P10, colonies were mechanically passaged with a needle. At P10, iPSC clones were adapted to feeder-free culture conditions: Vitronectin (VTN-N) Recombinant Human Protein, Truncated (5µg/mL, Life Technologies) with mTeSR (Stem Cell Technologies). Feeder-free iPSCs were passaged using the Passaging Solution XF (StemMACSTM, Miltenyi Biotec). Mycoplasma detection was realized by the MycoAlert™ kit (LONZA, LT07-318).

Genotype verification of patient-derived iPSCs

Genomic DNA was extracted using QIAamp DNA mini-kit and sent to Eurofins Genomics for sequencing (RSTS$_{CBP}$ iPSCs). For *EP300* PCR-amplification of a fragment encompassing the deleted region (10 kb) was performed.

PCR PRIMERS:
hEP300-in7-5p: CTGTTCTTGAACTCCTGACC
hEP300-ex12-3p: TTGAGAGCTCCAGGTTGAGC
hCREBBP_ex18_F: AGAATCCCATGGACCTCTCC
hCREBBP_ex18_R: ATCCAAGGGACTGCATGACA

RT-qPCR

Total RNA was extracted using RNeasy® columns and DNAse-treated using RNase-free DNase (Qiagen). For quantitative PCR, first-strand cDNAs were generated using 500ng of RNA, SuperScript™ II Reverse Transcriptase (Invitrogen), 25µg/ml polydT and 9.6µg/ml random primers (Invitrogen).
To quantitate transcripts, absolute quantitative PCR was performed on a StepOne (Applied Biosystems) using power SYBR green PCR master mix (Applied Biosystems), for genes listed in the primers table (end of Experimental Procedures). For each sample, the ratio of specific mRNA level relative to GAPDH levels was calculated. Experimental results are shown as levels of mRNA relative to the highest value. SeV expression was measured using primers recommended by the manufacturer.

PCR PRIMERS

Gene Name	Primer sequence 5'-3'	Amplicon size (bp)	Melting Temp. (°C)	Position
GAPDH	AATCCCATCACCATCTTCCA			
	TGGACTCCACGACGTACTCA	82	80.5	494-576
OCT4	TGGGTGGAGGAAGCTGACAACAAT			
	TTCGGGCACTGCAGGAACAAATTC	142	82.1	1005-1147
SOX2	CCTACTCGCAGCAGGGCACC			
	CTCGGCGCCGGGGAGATACA	169	78	1114-1283
NANOG	ATAGCAATGGTGTGACGCAGAAGG			
	CTGGTTGCTCCACATTGGAAGGTT	116	82	701-816

All primers have a hybridization temperature of 60°C. GAPDH, OCT4 and NANOG amplicons span two adjacent exons.

Early germ layer differentiation

hPSC lines were differentiated into endoderm, mesoderm and ectoderm using Stemmacs Trilineage Kit (Miltenyi biotec). 80,000 cells for mesoderm, 130,000 cells for endoderm and 100,000 cells for ectoderm were plated in 24 wells plates, and cultured in specific media for 7 days, as specified by the protocol. On day 7, differentiated cells were analyzed by flow cytometry and 3'SRP.

Expression profiling by 3'SRP

3' SRP data generation

For 3' SRP profiling, RNA-sequencing protocol was performed according to our implementation of Soumillon et al. protocol (DOI: https://doi.org/10.1101/003236 and DOI: 10.21203/rs.3.pex-1336/v1). Briefly, total RNA was extracted using RNeasy® columns and DNAse-treated using RNase-free DNase (Qiagen). The libraries were prepared from 10 ng of total RNA per samples. The mRNA poly(A) tails were tagged with universal adapters, well-specific barcodes and unique molecular identifiers (UMIs) during template-switching reverse transcription. Barcoded cDNAs from multiple samples were then pooled, amplified and tagmented using a transposon-fragmentation approach which enriches for 3'ends of cDNAs. A library of 350–800 bp was run on an Illumina® HiSeq 2500 using a Hiseq Rapid SBS Kit v2-50 cycles and a Hiseq Rapid PE Cluster Kit v2.

3' SRP data preprocessing

Read pairs used for analysis matched the following criteria: all 16 bases of the first read had quality scores of at least 10 and the first 6 bases correspond exactly to a designed well-specific barcode. The second reads were aligned to RefSeq human mRNA sequences (hg19) using bwa version 0.7.17. Reads mapping to several transcripts of different genes or containing more than 3 mismatches with the reference sequences were filtered out from the analysis. 3' SRP profiles were generated by counting for each sample the number of unique UMIs associated with each RefSeq genes. Sequenced samples with at least 50000 counts and 6000 expressed genes were retained for further analysis.

3' SRP analysis

Data were normalized using DESeq2 package. PCAs were computed with the normalized expression profiles. Normalized, log transformed, centered expression profiles were plotted as a heatmap.

SNP analysis
DNA was extracted from somatic and iPSCs samples using the QIAGEN QiaAmp kit, according to the manufacturer's recommendations. The gDNA was quantified and qualified using a nanodrop. 200 ng of gDNA was outsourced to Integragen Company (Evry, France) for karyotype analysis using HumanCore-24-v1 SNP arrays. This array contains over 300,000 probes distributed throughout the genome with a median coverage of one probe every 9,500 bases. All genomic positions were based upon Human Genome Build 37 (hg19).

DNA samples were hybridized on HumanCore-24-V1 SNP arrays according to the manufacturer's instructions by Integragen. Analysis was performed with GenomeStudio software (v1.0). Chromosome abnormalities were determined by visual inspection of logR ratios and B-allele frequencies (BAF) values and comparing parental cells and iPS-derived samples. LogR ratio, the ratio between observed and expected probe intensity, is informative regarding copy number variation (i.e. deletions/duplications) and BAF is informative regarding heterozygosity. We used the SNP data to compute CNV. In particular, this type of chips allows to detect loss of heterozigocy (LOH), an important concern for hiPSCs, which is not possible with classical CGH arrays. All iPSC had SNP profiles similar to parental lines.

Purification of HSF2 from HEK 293 cells and mass spectrometry analysis of acetylated lysines
The protocol used was the same as for HSF1, as described [23]. Briefly, HEK 293 cells were transfected with mouse HSF2-beta Flag with or without CMV-EP300, treated with 1 µM trichostatin A and 5 mM nicotinamide 18 h prior harvesting and lysis in RIPA buffer. HSF2-Flag was immunoprecipitated, using α-Flag M2 affinity gel beads (Sigma F2426), and eluted with Flag peptide. Purified mHSF2-Flag was separated by SDS-PAGE, excised from the gel, digested with trypsin, and subjected to tandem mass spectrometric analysis by a hybrid quadrupole time-of-flight instrument (QSTAR, Applied Biosystems, Foster City, CA) equipped with a nanoelectrospray source. MS/MS spectra were searched against the IPI mouse sequence database (68,222 entries; version 3.15) using Mascot (Matrix Science, Boston, MA; version 1.9.05) and X! Tandem (www.thegpm.org; version 2006.04.01.2) database 4 search algorithms. Mascot and X! Tandem were searched with a fragment and precursor ion mass tolerance of 0.3 Da assuming the digestion enzyme trypsin with the possibility of one missed cleavage. Carbamidomethylation of cysteine was included as a fixed modification whereas methionine oxidation, N-terminal protein and lysine acetylation were included as variable modifications in the database search. Peptide identifications were accepted at greater than 95.0% probability as determined by the Peptide Prophet algorithm 7 and validated by manual inspection of the MS/MS spectra, as shown in Supplementary Table 2. Related to Fig. 2C and Supplementary Fig. 2A and Supplementary Table 1.

Modelling of the HR-A/B domain and KIX domain of CBP
Prediction of secondary structure of the HSF2 HR-A/B domain was performed using Psipred (http://bioinf.cs.ucl.ac.uk/psipred/) and nps@ (https://npsa-prabi.ibcp.fr/). The tertiary structure of the same domain was predicted using http://petitjeanmichel.free.fr/itoweb.petitjean.freeware.html. Sequence similarity between human HSF2 HR-A/B[13], lipoprotein Lpp56 of *E. coli*[24], yeast transcriptional factor GCN4 (mutated on some residues to stabilize heptad repeats;[25]) and murine PTRF (Polymerase I and Transcript-Release Factor) and human ATF2 (Activating Transcription Factor 2; a member of the ATF/CREB family) that is known to interact with CBP/EP300[26] was explored using *Uniprot* ([27]; https://www.uniprot.org/) and *ClustalW* ([28]; https://www.ebi.ac.uk/Tools/msa/clustalw2/). Step 1: Based on this sequence similarity a sequence alignment of H-RA/B was developed (using *Uniprot and ClustalW*; see Supplementary Fig. 4e), a structural model of the monomer HRA/B was generated using using *Modeller* (v9.19 [29]; https://salilab.org/modeller/), verified by *ERRAT* ([30]; http://servicesn.mbi.ucla.edu/ERRAT/) and *RESprox* ([31]; http://www.resprox.ca/) and Ramachandran plot (see Supplementary Fig. 4e; [32]) followed by the development of the trimer using *SymmDock* ([33]; v Beta 1.0; http://bioinfo3d.cs.tau.ac.il/SymmDock/). Step 2: the interaction between HR-A/B and the KIX domain (pdb: 2LXT; [34]) was simulated and compared using *Zdock* (v 2.1; [35]) and *Firedock* [36,37]. More precisely, the ten best results generated by *Zdock* and *Firedock*, and scored according to their Root

Mean Square Deviation (RMSD), which were compared thanks to a visualization program ICM[38]. The mutation of the key residues involved in the interaction between HR-A/B and the KIX domain have been performed using PyMOL (v2.0) and the docking was done as described above.

Production of KIX-GST, His-CBP domain and HSF2 proteins

Escherichia coli 21 (DE3) were transformed with the different 6His-tag CBP constructs for production of the different CBP domains as previously described in [39] . All proteins were stored in 20 mM Tris-HCl, 150 mM NaCl, pH 7.5 and kept at -80°C until use. *E. coli* BL21 bacteria were transformed with the different GST-KIX constructs[40] and grown in presence of ampicillin and chloramphenicol at 37°C (4-6h). Bacteria were then grown with 1 mM Isopropyl β-D-1-thiogalactopyranoside over-night. After centrifugation (4,400 rpm at 4°C), bacteria were lysed in PBS pH 8, 300 mM NaCl, Triton X100 1%, 1 mg/mL lysozyme, protease inhibitors under stirring at 4°C for 30 min. Bacteria were sonicated (BRANSON sonicator, power 20%, 10’’ ON/20’’OFF) and centrifuged at 4°C, 16000g for 30 min. Gluthatione sepharose 4B beads (G4510-10ML Sigma-Aldrich) were added to the cleared supernatants and subjected to rotation for 1 h 30 min at 4°C. The mixture was loaded into a column (Sigma-Aldrich) and washed with PBS/NaCl 300 mM pH8, then Tris 50 mM/NaCl 150 mM pH 8. Proteins were eluted with 5mL of elution buffer (50 mM Tris HCl pH8, 150 mM NaCl, 10 mM GSH). The protein concentration was measured using the Bradford method. *In vitro* transcription and translation reactions were performed using a TNT T7-coupled reticulocyte lysate system as recommended by the supplier (Promega, Charbonnieres-les-Bains, France). 1 µG of the plasmid DNA template was transcribed and the protein was translated at 30°C for 90 min.

SNAP-Tag labelling of HSF2 molecules and analysis of protein decay

Principle: cells expressing SNAP-tagged HSF2 are incubated with a fluorescent SNAP substrate, which covalently labels the live pool of SNAP-HSF2. Addition of a non-fluorescent blocking substrate at a given time (t_0) prevents further labelling of newly synthesized SNAP-HSF2 and allows the follow up of the fluorescence decay as an estimation of HSF2 protein decay.

CRISPR/Cas9 *Hsf2*KO U2OS cells were transfected with SNAP-HSF2 WT, -HSF2 3KQ or -HSF2 3KR constructs (Xtrem-Gen HP, Sigma-Aldrich), incubated in the presence of the cell-permeable SNAP-Cell® Oregon green fluorescent substrate (1.25 mM) and then with SNAP-Cell® Block (0.5 mM) during the pulse chase according to the manufacturer’s instructions (New England Biolabs). Cells were lysed in modified Laemmli buffer (5% SDS, 10% glycerol, 32.9 mM Tris-HCl pH6.8) supplemented with 1 mM DTT (Sigma-Aldrich), and their extracts (15 µG) were run on 10% SDS-PAGE. Gels were then scanned on a Typhoon Trio imager (GE Healthcare; excitation 532 nm, emission 580 nm, PMT 700 V) for determination of signal intensity of the covalently-bound fluorescent products as described in [41].

Antibodies

Antibodies	species	Clone	reference	Manufacturer	WB	IP	IF	Validation
Acetyl-lysine (Pan)	rabbit	pAb	#9441	Cell signalling Technology	WB : 1/1000		IF: 1/100(	RRID: AB_331805
Actin	mouse	AC40	A3853	Sigma-Aldrich	WB : 1/4000			RRID:AB_262137
Alexa Fluor 488	mouse		715-546-151	J.ImmunoRes			IF:1/800	
Alexa Fluor 488	rabbit		A-11008	J.ImmunoRes			IF:1/800	
Alexa Fluor 594	rabbit		A-11037	J.ImmunoRes			IF:1/800	
CBP	rabbit IgG	D6C5	#7389	Cell signalling Technology	WB : 1/1000		IF:1/100	RRID:AB_2616020
CBP	rabbit	A-22	sc-369	Santa-Cruz	WB : 1/1000			RRID:AB_631006
Cy3TM-3	mouse		715-165-150	J.ImmunoRes			IF:1/800	
EP300/CBP	Mouse	pAb	sc-32244	Santa-Cruz			IF:1/25	RRID:AB_628076
EP300	rabbit	pAb	sc-584	Santa-Cruz	WB : 1/500		IF:1/200	RRID:AB_2293429
FABP7/BLBP	mouse	AT1D1	AM09059PU-S	Origen			IF:1/400	RRID:AB_1652805
Flag tag	mouse	M2	F1804	Sigma-Aldrich	WB : 1/100(	IP: 2μg		RRID:AB_262044
GFP tag	mouse	IgG1	MAB2510	Millipore	WB : 1/1000			RRID:AB_94623
GFP-Trap-A	mouse		gta-20	chromotek		IP: 25μL		RRID:AB_263135
GST tag	mouse	IgG	AE001	Ab Clonal	WB : 1/200(	IP: 2.5μL		RRID:AB_2770403
H3K18Ac	rabbit	pAb	GTX128943-S	Euromedex	WB : 1/2000			RRID: AB_2885843
H3K27Ac	rabbit	pAb	C15410174	Diagenode	WB : 1/1000			RRID: AB_2716835
HA tag	mouse	16B12	MMS101R	Covance	WB : 1/2000		IF:36526	RRID:AB_291262
HDAC1	rabbit	pAb	ab7028	Abcam	WB : 1/4000		IF:1/900	RRID:AB_305705
HRP mouse	mouse		115 035 135	J.ImmunoRes	WB : 1/50 000			
HRP mouse Fab	mouse	F(ab')2	115 036 072	J.ImmunoRes	WB : 1/50 000			
HRP rabbit	rabbit	IgG1	211 032 171	J.ImmunoRes	WB : 1/50 000			
HSC70	rat	mAb	ADI-SPA-815	Stressgen	WB : 1/1000			RRID:AB_10617277
HSF1	rabbit	pAb	#4356	Cell signalling Technology	WB : 1/1000		IF:1/800	RRID:AB_2861388
HSF2	mouse	G11	sc-74529	Santa-Cruz	WB : 1/250	IP: 4μg		Mouse HSF2-/-KO (Suppl.8b)
HSF2	mouse	3E2	ab69621	abcam		IP: 4μg		El Fatimy et al., (2014)
HSF2	rabbit	pAb	SFI57	Lea Sistonen Lab (Ostling et al., 2007)			IF:1/600	Mouse HSF2-/-KO; Ostling et al., (2007)
HSP70	mouse	C92F3A-5	ADI-SPA-810	Stressgen	WB : 1/1000			RRID: AB_2039260
HSP90	mouse	H9010	SMC-107	Stressmarq	WB : 1/3000			RRID: AB_2697870
HSP110	mouse	58F12	NCL-HSP105	Leica Biosystem	WB : 1/10 000			RRID: AB_563775
HuC/D	mouse	16A11	A21271	Thermoscientific			IF:1/500	RRID: AB_221448
IgG	mouse		I5381	sigma-Aldrich				
Myc tag	mouse	9B11	#2276	Cell signalling Technology	WB : 1/1000			RRID: AB_331783
Myc-Trap-A	mouse		yta-20	chromotek		IP: 25μL		RRID: AB_2631369
Nanog	rabbit	pAb	14295-1-AP	Proteintech			IF:1/200	RRID: AB_1607719
Nde1	rabbit		10233-1-AP	Proteintech	WB : 1/1500			RRID: AB_2149877
Nestin	mouse	10C2	MA1-110	Invitrogen			IF:1/200	RRID: AB_2536821
N-cadherin	rabbit		22018-1-AP	Proteintech	WB : 1/2000			RRID: AB_2813891
N-cadherin	mouse	GC4	C2542-100UL	Sigma			IF:1/1000	RRID: AB_258801
Oct-3/4	rabbit	EPR17929	Ab181557	Abcam			IF:1/200	RRID: AB_2687916
Pax 6	rabbit	pAb	12323-1-AP	Proteintech			IF:1/500	RRID: AB_2159695
PCNA	rabbit		PA5 #272-14	Thermoscientific	WB : 1/1000			RRID: AB_2544690
Phospho-Histone H3	rat	HTA28	ab10543	Abcam			IF:1/200	RRID: AB_2295065
Snap	rabbit	pAb	P9310	NEB	WB : 1/1000			RRID: AB_10631145
Sox 2	rabbit	pAb	ab97959	Abcam			IF:1/200	RRID: AB_2341193
	Trap-A CTL		bab-20	Chromotek		IP: 25μL		RRID: AB_2827547
Anti-TJP1/ZO-1	mouse	1A12	33-9100	FisherScientifique			IF:1/1000	RRID: AB_2533147
TBR1 coralite 594	mouse	IgG1-k	CL594-66564	Proteintech			IF:1/500	RRID: AB_2529847
Tuj-1	mouse	2G10	T8578	sigma-Aldrich			IF:1/1000	RRID: AB_1841228
Ubiquitin	mouse	FK2	BML-PW8810-0100	enzolifes sciences	WB : 1/500			RRID: AB_10541840

Plasmids and constructs

Plasmid name	Reference	vector	Origin	Supplier
Cas9 guide RNA HSF2	Cong et al., 2013	pX300 Cas9	human	
CBP DN-HA				
CBP-HA	kind gift of Pr. Wei Gu	pcDNA3-CMV	mouse	
EP300-HA	kind gift from W Sellers (East Tennessee State University, USA?); Duval et al., 2015	1246 pCMVb MycHA	human	Addgene # U10718
GFP binder nanobody (GBP)	Kind gift from P.A. Defossez (University Paris-Diderot, France), Zolghadr et al., 2008			
GFP tag		pEGFP-N1		Clontech (6085-1)
HDAC1 DN(D181A)-Flag	Kuzmochka et al., 2014	pcDNA3.1	human	
HDAC1-FLAG	Emiliani et al., 1998	pcDNA3.1	human	
HDAC1-GFP	kind gift from J Steve	eGFP-C3	human	
HDAC2-MYC	kind gift of Tony Kouzarides lab	pcDNA3.1/myc-HisA	mouse	evex 305
HDAC3-MYC	kind gift of Tony Kouzarides lab	pCMV3-Amyc	human	evex 476
His3.3-Snap	kind gift of Dr. S. Polo. (University Paris-Diderot, France)	pSNAPf	human	NEB (N9183)
HSF2alpha WT and mutant-Snap	cf materials and methods	pSNAPf	human	NEB (N9181)
HSF2alpha-CTAP(GS)-Gw	Bürckstümmer et al., 2006	PCEMM-CTAP	mouse	Euroscarf
HSF2alpha-Myc	Alastalo et al., 2003	pcDNA4™/TO/myc-His-A	human	Life Technologie
HSF2beta-CTAP(GS)-Gw	Bürckstümmer et al., 2006	PCEMM-CTAP	mouse	Euroscarf
HSF2beta-Flag	Pirkkala et al., 2000	pFLAG-CMV-2	mouse	Sigma-Aldrich
HSF2beta-Flag deletion mutants	Alastalo et al., 2003	pFLAG-CMV-2	mouse	Sigma-Aldrich
HSF2beta-YFP	cf materials and methods	pEYFP-C1	mouse	Clontech
Kix WT-GST	kind gift from Dr. Lemassson; Yan et al., 1998	pGEX-2T	mouse	
Kix Y650AY-GST	kind gift from Dr. Lemassson; Cook et al., 2011	pGEX-2T	mouse	
Myc tag		pcDNA3.1 MycHis		Life technology (V80020)

SUPPLEMENTARY NOTES

SUPPLEMENTARY ACKNOWLEDGEMENTS

We thank Slimane AIT-SI-ALI, Pierre-Antoine DEFOSSEZ, Claire ROUGELLE (UMR7216) and Vanessa RIBES (Institut Jacques Monod, (IJM) Paris, France) for helpful discussions and comments on the manuscript, Anne PLESSIS (IJM) for helpful discussions on setting the SNAP-TAG technology, Anne VANET (Institut Jacques Monod, Paris, France) for helpful discussions on the HSF2 structural modelling. We thank Pierre-Antoine DEFOSSEZ and Laure FERRY (UMR7216) for helpful guidance in F3H and GFP-Trap experiments, Clara GIANFERMI (UMR7216) for microscopy pictures of organoids and nSBs. We are very grateful to Isabelle LE PARCO and her Team at the Buffon animal housing facility (Institut Jacques Monod, Paris, France) for her management of the Covid-19 pandemic period.

SUPPLEMENTARY DISCUSSION

Interactions HSF2 and EP300

A similar mode of interaction between the HSF2 KIX-binding motifs and the residue corresponding to Y650 in EP300 can be expected[42].

HSF2 stabilization in neural development models

It should be noted that BTZ unexpectedly failed to induce HSF2 protein levels, both in iNPCs and hCO at different of differentiation, in contrast to MG132. This could be explained by the fact that the mechanism of action of BTZ on the proteasome and its output can differ from that of MG132, in different cell systems[43]. One possibility in the neural context that is very sensitive to HSF2 levels, is that BTZ has too strong effect on HSF2 levels compared to MG132[15,44]and might trigger compensatory mechanisms. These could thus prevent HSF2 marked increase by BTZ, in contrast to what is classically observed in non-neural cell systems[15,44].

General impact of the impairment of the HSF pathway in the multifaceted RSTS pathology

During the last decade, HSFs have been associated with a wide spectrum of pathophysiological conditions, and the specific roles of HSFs, either individually or in combination with each other or with other transcription factors, are of great biomedical interest. Especially, the mechanisms by which the expression levels of HSFs are regulated, in a context-dependent manner, have remained poorly understood. However, there is a wealth of documented cases where either excessive or insufficient HSF protein levels favor the development or progression of devastating diseases, including cancer, neurodevelopmental, and neurodegenerative disorders. The above cited deleterious *de novo* variant of HSF2 associated to Angelman Syndrome[45] , thus remarkedly extends the implication of HSF2 in monogenic NDDs.

The dysregulation of the HSF2 pathway in RSTS, so sensitive to various conditions, might contribute to the poor correlation existing between a given CBP/EP300 genotype and the clinical features observed in the corresponding RSTS patients, and may represent one avenue worth exploring in the future. Moreover, there might be broader involvement of the HSF pathway in other aspects of the RSTS pathology. RSTS patients suffer from extreme vulnerability to airway infections, which is mainly due to defects in mounting a response to polysaccharides[46,47]. Because the HSF pathway is involved in response to polysaccharides, inflammatory and immune responses, as well as lung protection against stress, its deregulation could also contribute to this aspect of the pathology[48-51] . Any imbalance in the delicate composition of the repertoire of HSPs under unstressed conditions and alteration in the triggering of HSF-driven stress-responses could profoundly influence vulnerability to infections. In addition, the disequilibrium between HSF1 and HSF2 activities in RSTS cells might also participate to RSTS vulnerability to cancer. Indeed, HSF1 is a wide and potent facilitator of cancer and HSF2 is also involved in cancer, in particular through the regulation of a large repertoire of genes beyond the classical *HSPs*, including members of the Cadherin superfamily[52-55].

Rare diseases are in the center of growing interest based on the recent acceptance that they represent a global public health and economic problem. For example, RSTS, despite its rarity (1:100,000 births) represents one on 300 patients institutionalized for intellectual disability[56]. Moreover, they are conceptually considered as extreme components in the spectrum of a large diversity of diseases, and their study has the strong potential to highlight shared features in related common disorders, that can thus be transposable to other pathologies and deeply transform our ways to comprehend these diseases. The functional impact of the regulation of HSF2 stability revealed in the context of RSTS might also represent an important reading key in related diseases, including neurodevelopmental disorders, as it was also shown for the abnormal activation of HSF1 and HSF2 in fetal alcohol syndrome and Angelman syndrome[3,45,57] .

SUPPLEMENTARY REFERENCES

1. Kallio M, Chang Y, Manuel M, Alastalo TP, Rallu M, Gitton Y, Pirkkala L, Loones MT, Paslaru L, Larney S, Hiard S, Morange M, Sistonen L, Mezger V. Brain abnormalities, defective meiotic chromosome synapsis and female subfertility in HSF2 null mice. *EMBO J.* **21**, 2591–2601 (2002).
2. Chang Y, Ostling P, Akerfelt M, Trouillet D, Rallu M, Gitton Y, El Fatimy R, Fardeau V, Le Crom S, Morange M, Sistonen L, Mezger V. Role of heat-shock factor 2 in cerebral cortex formation and as a regulator of p35 expression. *Genes Dev.* **20**, 836–847 (2006).
3. El Fatimy R, Miozzo F, Le Mouël A, Abane R, Schwendimann L, Sabéran-Djoneidi D, de Thonel A, Massaoudi I, Paslaru L, Hashimoto-Torii K, Christians E, Rakic P, Gressens P, Mezger V. Heat shock factor 2 is a stress-responsive mediator of neuronal migration defects in models of fetal alcohol syndrome. *EMBO Mol. Med.* **6**, 1043–1061 (2014).
4. Duchateau, A., de Thonel, A., El Fatimy, R., Dubreuil, V. & Mezger, V. The 'HSF connection': Pleiotropic regulation and activities of Heat Shock Factors shape pathophysiological brain development. *Neurosci. Lett.* **725**, 134895 (2020).
5. Rallu M, Loones M, Lallemand Y, Morimoto R, Morange M, Mezger V. Function and regulation of heat shock factor 2 during mouse embryogenesis. *Proc. Natl. Acad. Sci. U. S. A.* **94**, 2392–2397 (1997).
6. Kawasaki, H. *et al.* Distinct roles of the co-activators p300 and CBP in retinoic-acid-induced F9-cell differentiation. *Nature* **393**, 284–289 (1998).
7. Yao TP, Oh SP, Fuchs M, Zhou ND, Ch'ng LE, Newsome D, Bronson RT, Li E, Livingston DM, Eckner RGene dosage-dependent embryonic development and proliferation defects in mice lacking the transcriptional integrator p300. *Cell* **93**, 361–372 (1998).
8. Partanen, A., Motoyama, J. & Hui, C. C. Developmentally regulated expression of the transcriptional cofactors/histone acetyltransferases CBP and p300 during mouse embryogenesis. *Int. J. Dev. Biol.* **43**, 487–494 (1999).
9. Bhattacherjee V, Horn KH, Singh S, Webb CL, Pisano MM, Greene RM. CBP/p300 and associated transcriptional co-activators exhibit distinct expression patterns during murine craniofacial and neural tube development. *Int. J. Dev. Biol.* **53**, 1097–1104 (2009).
10. Anckar, J. & Sistonen, L. Regulation of HSF1 function in the heat stress response: implications in aging and disease. *Annu. Rev. Biochem.* **80**, 1089–1115 (2011).
11. Huttlin, E. L. Ting L, Bruckner RJ, Gebreab F, Gygi MP, Szpyt J, Tam S, Zarraga G, Colby G, Baltier K, Dong R, Guarani V, Vaites LP, Ordureau A, Rad R, Erickson BK, Wühr M, Chick J, Zhai B, Kolippakkam D, Mintseris J, Obar RA, Harris T, Artavanis-Tsakonas S, Sowa ME, De Camilli P, Paulo JA, Harper JW, Gygi SP. The BioPlex Network: A Systematic Exploration of the Human Interactome. *Cell* **162**, 425–440 (2015).
12. Tang S, Chen H, Cheng Y, Nasir MA, Kemper N, Bao E. The interactive association between heat shock factor 1 and heat shock proteins in primary myocardial cells subjected to heat stress. *Int. J. Mol. Med.* **37**, 56–62 (2016).
13. Sandqvist A, Björk JK, Akerfelt M, Chitikova Z, Grichine A, Vourc'h C, Jolly C, Salminen TA, Nymalm Y, Sistonen L. Heterotrimerization of heat-shock factors 1 and 2 provides a transcriptional switch in response to distinct stimuli. *Mol. Biol. Cell* **20**, 1340–1347 (2009).
14. Shu, W., Ji, H. & Lu, M. Trimerization specificity in HIV-1 gp41: analysis with a GCN4 leucine zipper model. *Biochemistry* **38**, 5378–5385 (1999).
15. Joutsen J, Da Silva AJ, Luoto JC, Budzynski MA, Nylund AS, de Thonel A, Concordet JP, Mezger V, Sabéran-Djoneidi D, Henriksson E, Sistonen L. Heat Shock Factor 2 Protects against Proteotoxicity by Maintaining Cell-Cell Adhesion. *Cell Rep.* **30**, 583-597.e6 (2020).
16. Mosser, D. D., Theodorakis, N. G. & Morimoto, R. I. Coordinate changes in heat shock element-binding activity and HSP70 gene transcription rates in human cells. *Mol. Cell. Biol.* **8**, 4736–4744 (1988).

17. Abravaya, K., Phillips, B. & Morimoto, R. I. Heat shock-induced interactions of heat shock transcription factor and the human hsp70 promoter examined by in vivo footprinting. *Mol. Cell. Biol.* **11**, 586–592 (1991).
18. Jin Q, Yu LR, Wang L, Zhang Z, Kasper LH, Lee JE, Wang C, Brindle PK, Dent SY, Ge K. Distinct roles of GCN5/PCAF-mediated H3K9ac and CBP/p300-mediated H3K18/27ac in nuclear receptor transactivation. EMBO J. 30, 249-262 (2011).
19. Yu J, Vodyanik MA, Smuga-Otto K, Antosiewicz-Bourget J, Frane JL, Tian S, Nie J, Jonsdottir GA, Ruotti V, Stewart R, Slukvin II, Thomson JA. Induced pluripotent stem cell lines derived from human somatic cells. *Science* **318**, 1917-1920 (2007)
20. Yu J, Hu K, Smuga-Otto K, Tian S, Stewart R, Slukvin II, Thomson JA. Human induced pluripotent stem cells free of vector and transgene sequences. *Science* **324**, 797-801 (2009).
21. Chen G, Gulbranson DR, Hou Z, Bolin JM, Ruotti V, Probasco MD, Smuga-Otto K, Howden SE, Diol NR, Propson NE, Wagner R, Lee GO, Antosiewicz-Bourget J, Teng JM, Thomson JA. Chemically defined conditions for human iPSC derivation and culture. *Nat Methods* **8**:424-9 (2011)
22. Hu K, Yu J, Suknuntha K, Tian S, Montgomery K, Choi KD, Stewart R, Thomson JA, Slukvin II. Efficient generation of transgene-free induced pluripotent stem cells from normal and neoplastic bone marrow and cord blood mononuclear cells. *Blood* **117**:109-19 (2011).
23. Westerheide, S. D., Anckar, J., Stevens, S. M., Sistonen, L. & Morimoto, R. I. Stress-inducible regulation of heat shock factor 1 by the deacetylase SIRT1. *Science* **323**, 1063–1066 (2009).
24. Shu, W., Liu, J., Ji, H. & Lu, M. Core structure of the outer membrane lipoprotein from Escherichia coli at 1.9 A resolution. *J. Mol. Biol.* **299**, 1101–1112 (2000).
25. Shu, W., Ji H., Lu M. Trimerization specificity in HIV-1 gp41: analysis with a GCN4 leucine zipper model. *Biochemistry*. **38**:5378-85 (1999).
26. Bordoli L, Hüsser S, Lüthi U, Netsch M, Osmani H, Eckner R. Functional analysis of the p300 acetyltransferase domain: the PHD finger of p300 but not of CBP is dispensable for enzymatic activity. *Nucleic Acids Res.* **29**, 4462–4471 (2001).
27. Pundir, S., Martin, M. J. & O'Donovan, C. UniProt Protein Knowledgebase. *Methods Mol. Biol. Clifton NJ* **1558**, 41–55 (2017).
28. Thompson, J. D., Higgins, D. G. & Gibson, T. J. CLUSTAL W: improving the sensitivity of progressive multiple sequence alignment through sequence weighting, position-specific gap penalties and weight matrix choice. *Nucleic Acids Res.* **22**, 4673–4680 (1994).
29. Webb B, Sali A. Comparative protein structure modeling using Modeller. *Curr. Protoc. Bioinforma.* **Chapter 5**, Unit-5.6 (2006).
30. Colovos, C. & Yeates, T. O. Verification of protein structures: patterns of nonbonded atomic interactions. *Protein Sci. Publ. Protein Soc.* **2**, 1511–1519 (1993).
31. Berjanskii, M., Zhou, J., Liang, Y., Lin, G. & Wishart, D. S. Resolution-by-proxy: a simple measure for assessing and comparing the overall quality of NMR protein structures. *J. Biomol. NMR* **53**, 167–180 (2012).
32. Ramachandran, G. N., Ramakrishnan, C. & Sasisekharan, V. Stereochemistry of polypeptide chain configurations. *J. Mol. Biol.* **7**, 95–99 (1963).
33. Schneidman-Duhovny, D., Inbar, Y., Nussinov, R. & Wolfson, H. J. PatchDock and SymmDock: servers for rigid and symmetric docking. *Nucleic Acids Res.* **33**, W363-367 (2005).
34. Brüschweiler, S., Konrat, R. & Tollinger, M. Allosteric communication in the KIX domain proceeds through dynamic repacking of the hydrophobic core. *ACS Chem. Biol.* **8**, 1600–1610 (2013).
35. Pierce, B. G. *et al.* ZDOCK server: interactive docking prediction of protein-protein complexes and symmetric multimers. *Bioinforma. Oxf. Engl.* **30**, 1771–1773 (2014).
36. Mashiach, E., Schneidman-Duhovny, D., Andrusier, N., Nussinov, R. & Wolfson, H. J. FireDock: a web server for fast interaction refinement in molecular docking. *Nucleic Acids Res.* **36**, W229-232 (2008).
37. Odoux A, Jindal D, Tamas TC, Lim BW, Pollard D, Xu W. Experimental and molecular dynamics studies showed that CBP KIX mutation affects the stability of CBP:c-Myb complex. *Comput. Biol. Chem.* **62**, 47–59 (2016).

38. Fernandez-Recio, J., Totrov, M., Skorodumov, C. & Abagyan, R. Optimal docking area: a new method for predicting protein-protein interaction sites. *Proteins* **58**, 134–143 (2005).
39. Duval R, Fritsch L, Bui LC, Berthelet J, Guidez F, Mathieu C, Dupret JM, Chomienne C, Ait-Si-Ali S, Rodrigues-Lima F. An acetyltransferase assay for CREB-binding protein based on reverse phase-ultra-fast liquid chromatography of fluorescent histone H3 peptides. *Anal. Biochem.* **486**, 35–37 (2015).
40. Cook, P. R., Polakowski, N. & Lemasson, I. HTLV-1 HBZ protein deregulates interactions between cellular factors and the KIX domain of p300/CBP. *J. Mol. Biol.* **409**, 384–398 (2011).
41. Sanial M, Bécam I, Hofmann L, Behague J, Argüelles C, Gourhand V, Bruzzone L, Holmgren RA, Plessis A. Dose-dependent transduction of Hedgehog relies on phosphorylation-based feedback between the G-protein-coupled receptor Smoothened and the kinase Fused. *Dev. Camb. Engl.* **144**, 1841–1850 (2017).
42. Kauppi M, Murphy JM, de Graaf CA, Hyland CD, Greig KT, Metcalf D, Hilton AA, Nicola NA, Kile BT, Hilton DJ, Alexander WS. Point mutation in the gene encoding p300 suppresses thrombocytopenia in Mpl-/- mice. *Blood* **112**, 3148–3153 (2008).
43. Harhouri K, Navarro C, Depetris D, Mattei MG, Nissan X, Cau P, De Sandre-Giovannoli A, Lévy N. MG132-induced progerin clearance is mediated by autophagy activation and splicing regulation. *EMBO Mol. Med.* **9**, 1294–1313 (2017).
44. Rossi A, Riccio A, Coccia M, Trotta E, La Frazia S, Santoro MG. The proteasome inhibitor bortezomib is a potent inducer of zinc finger AN1-type domain 2a gene expression: role of heat shock factor 1 (HSF1)-heat shock factor 2 (HSF2) heterocomplexes. *J. Biol. Chem.* **289**, 12705–12715 (2014).
45. Aguilera C, Gabau E, Ramirez-Mallafré A, Brun-Gasca C, Dominguez-Carral J, Delgadillo V, Laurie S, Derdak S, Padilla N, de la Cruz X, Capdevila N, Spataro N, Baena N, Guitart M, Ruiz A. New genes involved in Angelman syndrome-like: Expanding the genetic spectrum. *PloS One* **16**, e0258766 (2021).
46. Naimi, D. R., Munoz, J., Rubinstein, J. & Hostoffer, R. W. Rubinstein-Taybi syndrome: an immune deficiency as a cause for recurrent infections. *Allergy Asthma Proc.* **27**, 281–284 (2006).
47. Herriot, R. & Miedzybrodzka, Z. Antibody deficiency in Rubinstein-Taybi syndrome. *Clin. Genet.* **89**, 355–358 (2016).
48. Xiao, X. *et al.* HSF1 is required for extra-embryonic development, postnatal growth and protection during inflammatory responses in mice. *EMBO J.* **18**, 5943–5952 (1999).
49. Inouye S, Fujimoto M, Nakamura T, Takaki E, Hayashida N, Hai T, Nakai A. Heat shock transcription factor 1 opens chromatin structure of interleukin-6 promoter to facilitate binding of an activator or a repressor. *J. Biol. Chem.* **282**, 33210–33217 (2007).
50. Wirth, D., Christians, E., Li, X., Benjamin, I. J. & Gustin, P. Use of Hsf1(-/-) mice reveals an essential role for HSF1 to protect lung against cadmium-induced injury. *Toxicol. Appl. Pharmacol.* **192**, 12–20 (2003).
51. Budzyński, M. A., Puustinen, M. C., Joutsen, J. & Sistonen, L. Uncoupling Stress-Inducible Phosphorylation of Heat Shock Factor 1 from Its Activation. *Mol. Cell. Biol.* **35**, 2530–2540 (2015).
52. Dai, C., Whitesell, L., Rogers, A. B. & Lindquist, S. Heat shock factor 1 is a powerful multifaceted modifier of carcinogenesis. *Cell* **130**, 1005–1018 (2007).
53. Mendillo ML, Santagata S, Koeva M, Bell GW, Hu R, Tamimi RM, Fraenkel E, Ince TA, Whitesell L, Lindquist S. HSF1 drives a transcriptional program distinct from heat shock to support highly malignant human cancers. *Cell* **150**, 549–562 (2012).
54. Santagata S, Mendillo ML, Tang YC, Subramanian A, Perley CC, Roche SP, Wong B, Narayan R, Kwon H, Koeva M, Amon A, Golub TR, Porco JA Jr, Whitesell L, Lindquist S. Tight coordination of protein translation and HSF1 activation supports the anabolic malignant state. *Science* **341**, 1238303 (2013).
55. Björk JK, Åkerfelt M, Joutsen J, Puustinen MC, Cheng F, Sistonen L, Nees M. Heat-shock factor 2 is a suppressor of prostate cancer invasion. *Oncogene* **35**, 1770–1784 (2016).
56. Spena, S., Gervasini, C. & Milani, D. Ultra-Rare Syndromes: The Example of Rubinstein-Taybi Syndrome. *J. Pediatr. Genet.* **4**, 177–186 (2015).

57. Hashimoto-Torii K, Torii M, Fujimoto M, Nakai A, El Fatimy R, Mezger V, Ju MJ, Ishii S, Chao SH, Brennand KJ, Gage FH, Rakic P. Roles of heat shock factor 1 in neuronal response to fetal environmental risks and its relevance to brain disorders. *Neuron* **82**, 560–572 (2014).

SUPPLEMENTARY FIGURES SOURCE DATA

Raw data are provided via *Figshare* and are available through the following links:

FIGSHARE LINKS_SOURCE DATA NCOMMS-20-19437A (The DOI becomes active when the item is published).

FigS1 : DOI : 10.6084/m9.figshare.20170520

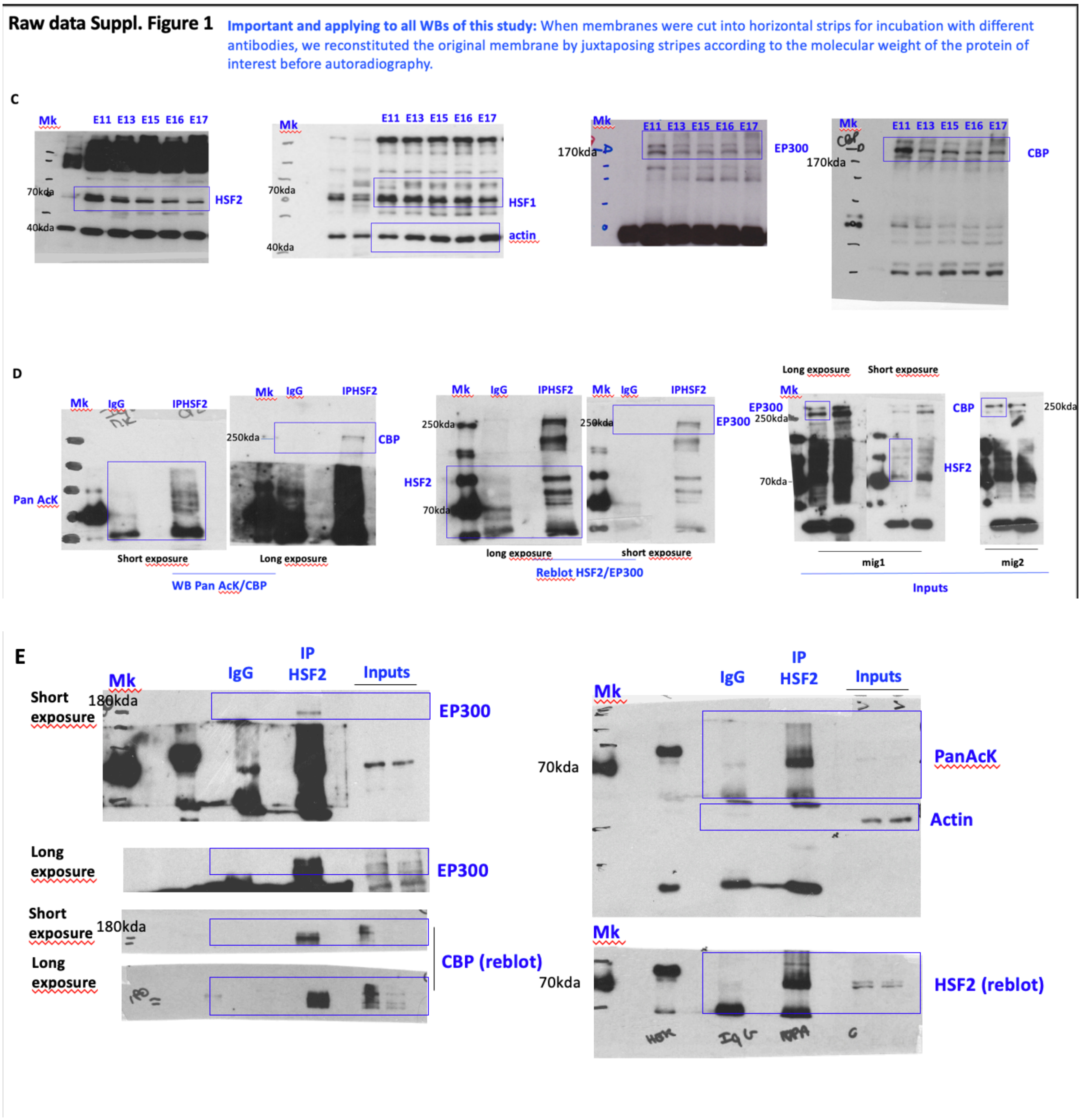

FigS2 : DOI: 10.6084/m9.figshare.20170529

Raw data Suppl. Figure 2

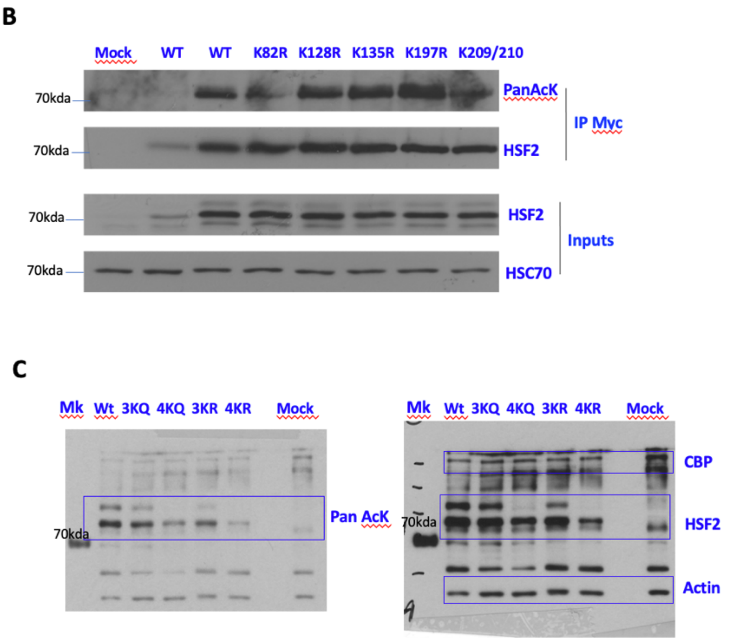

FigS3: DOI: 10.6084/m9.figshare.20170532

Raw data Suppl. Figure 3

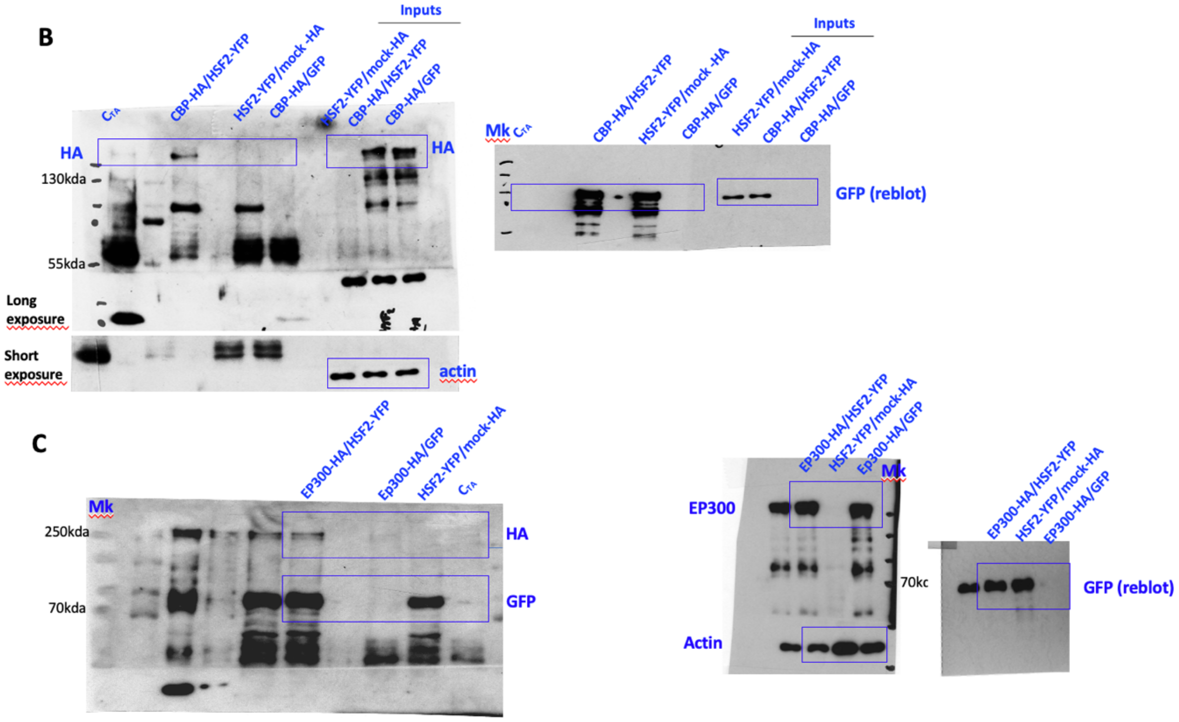

FigS4: DOI: 10.6084/m9.figshare.20170538

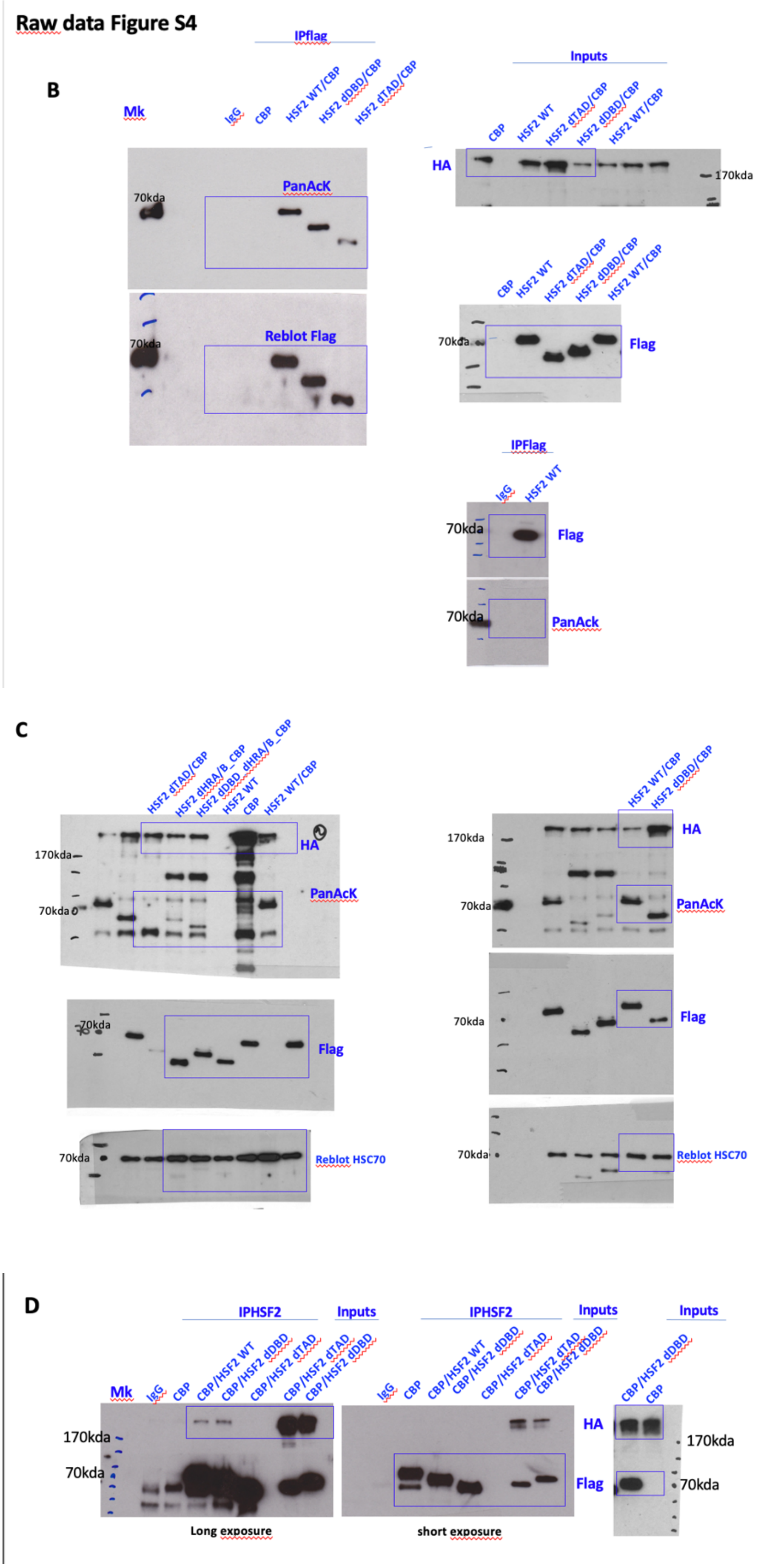

FigS5: DOI: 10.6084/m9.figshare.20170544

Raw data Suppl.Figure 5

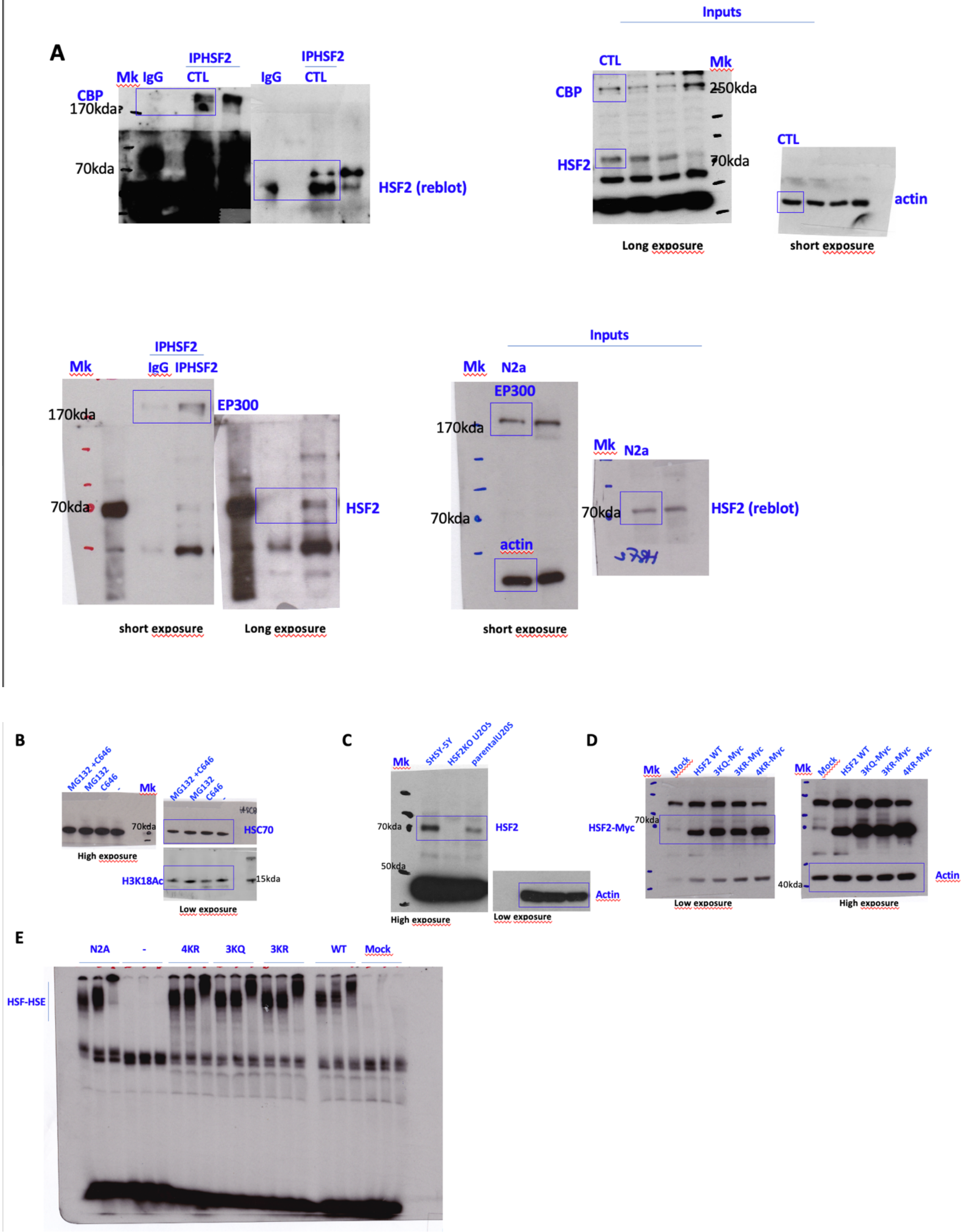

FigS6: DOI: 10.6084/m9.figshare.20170547

Raw data Suppl.Figure 6

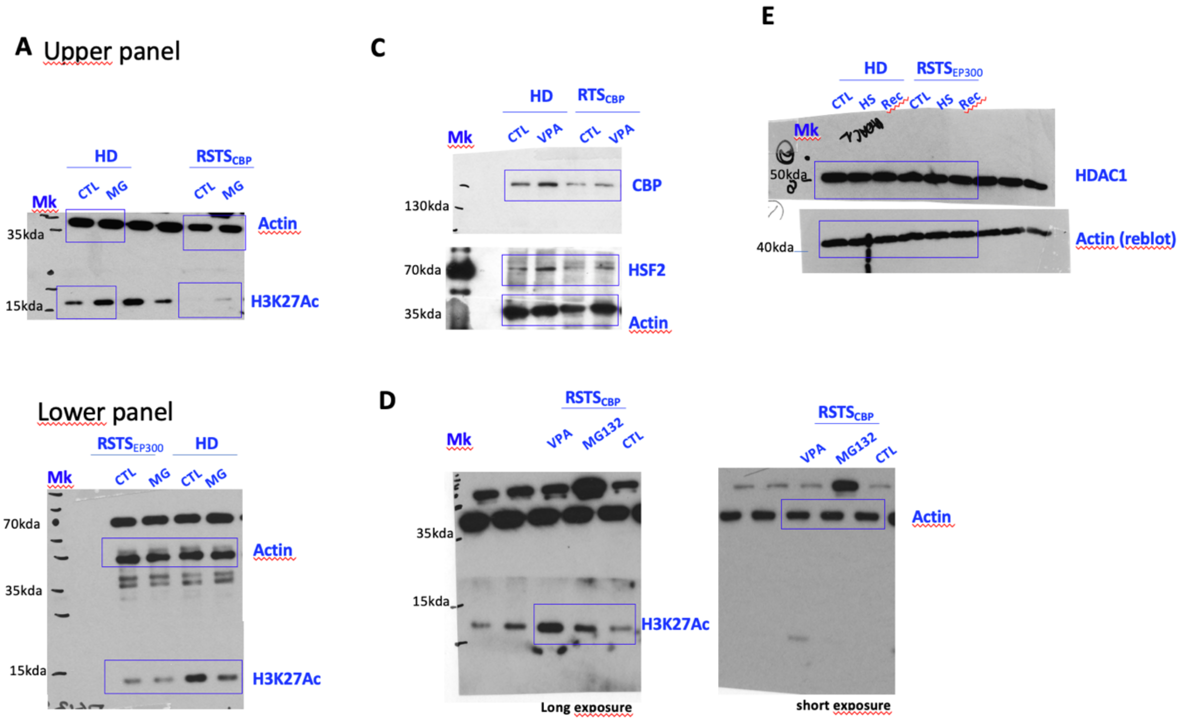

FIgS7: DOI: 10.6084/m9.figshare.20170556

No western-blot in supplementary Figure 7

FigS8: DOI: 10.6084/m9.figshare.20172449

Raw data Suppl. Figure 8

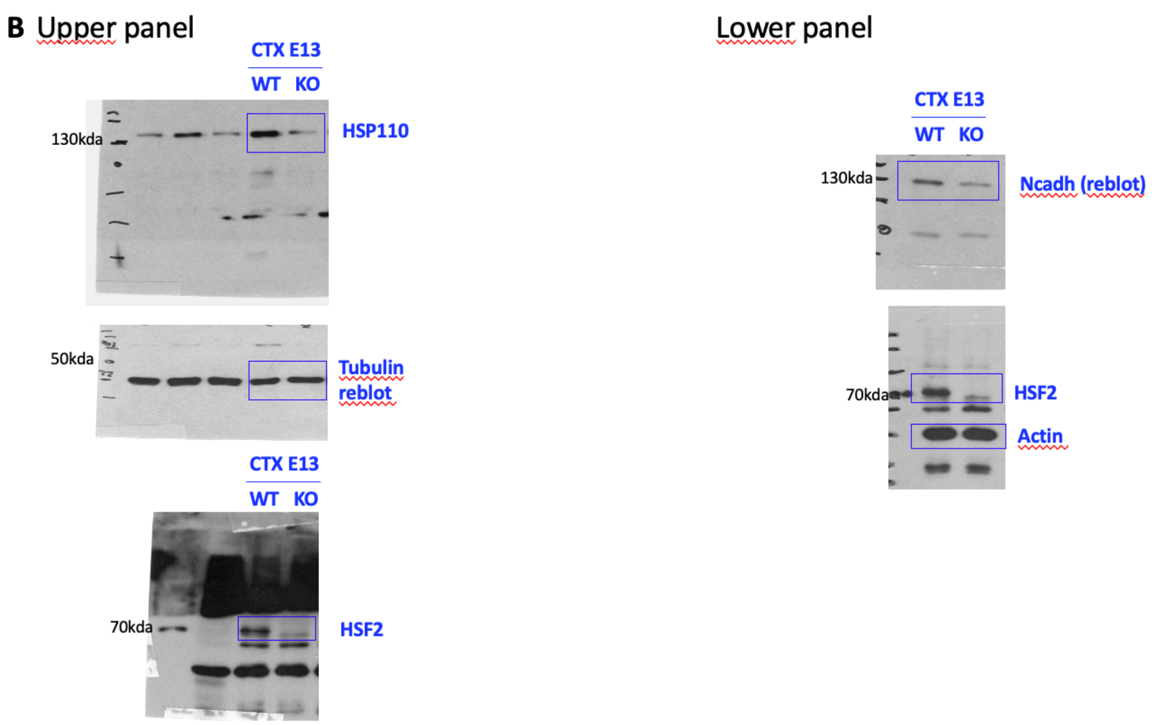

FigS9: DOI:10.6084/m9.figshare.20173469

No western-blot in supplementary Figure 9

FigS10: DOI: 10.6084/m9.figshare.20173484

Raw data Suppl.Figure 10

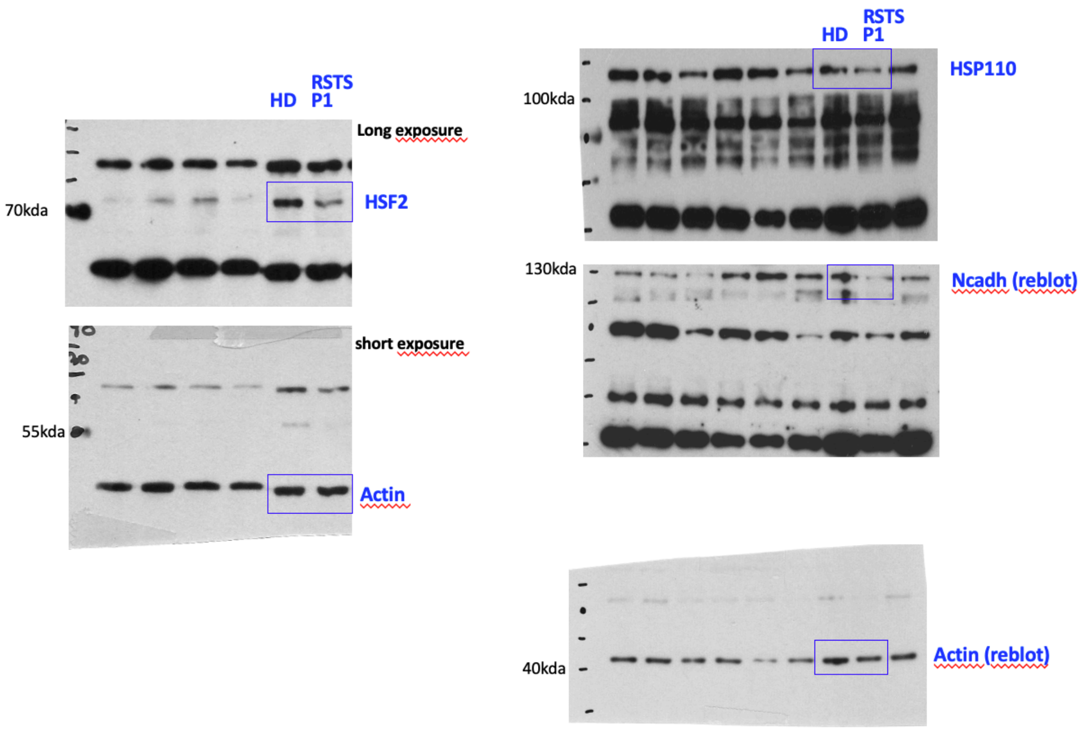

FigS11: DOI: 10.6084/m9.figshare.20173487

Raw data Suppl.Figure 11

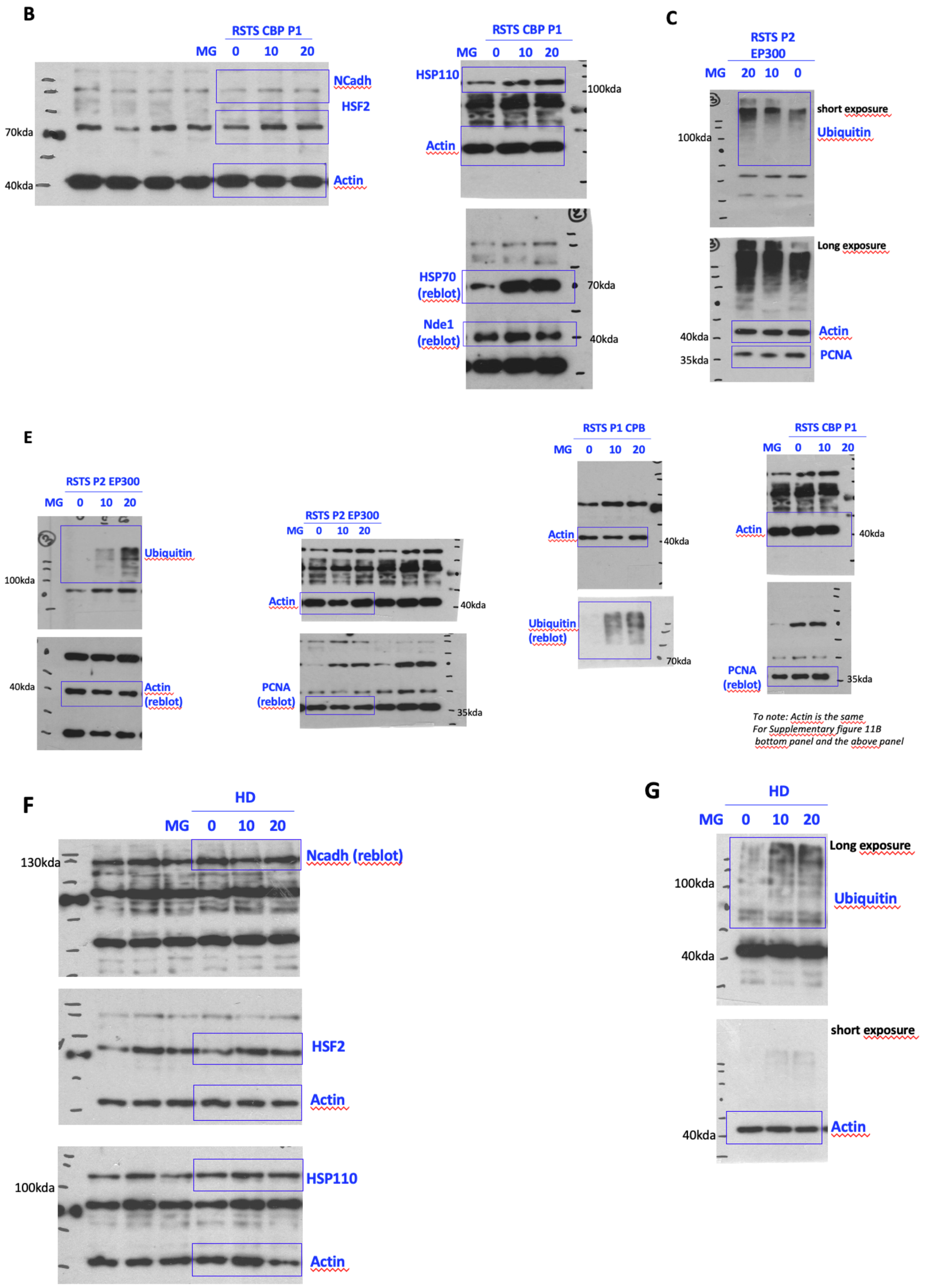

Supplement: Supplementary file 1 — Supplementary Information [file 41467_2022_34476_MOESM1_ESM.pdf]
