# Supplementary material for: CBP-HSF2 structural and functional interplay in Rubinstein-Taybi neurodevelopmental disorder
Source: Nat Commun. 2022 Nov 16;13:7002. doi: 10.1038/s41467-022-34476-2 (PMC9668993; doi:10.1038/s41467-022-34476-2)
Supplement: Supplementary file 3 — Description of Additional Supplementary Files [file 41467_2022_34476_MOESM3_ESM.pdf]

Description of Additional Supplementary Files

Supplementary Data 1 Original tables and spectra corresponding to the HSF2 acetylated peptides identified by MS. (relative to Fig. 2c and Supplementary Fig. 2a)
