# Supplementary material for: CBP-HSF2 structural and functional interplay in Rubinstein-Taybi neurodevelopmental disorder
Source: Nat Commun. 2022 Nov 16;13:7002. doi: 10.1038/s41467-022-34476-2 (PMC9668993; doi:10.1038/s41467-022-34476-2)
Supplement: Supplementary file 4 — Supplementary Data 1 [file 41467_2022_34476_MOESM4_ESM.pdf]

#1 K82

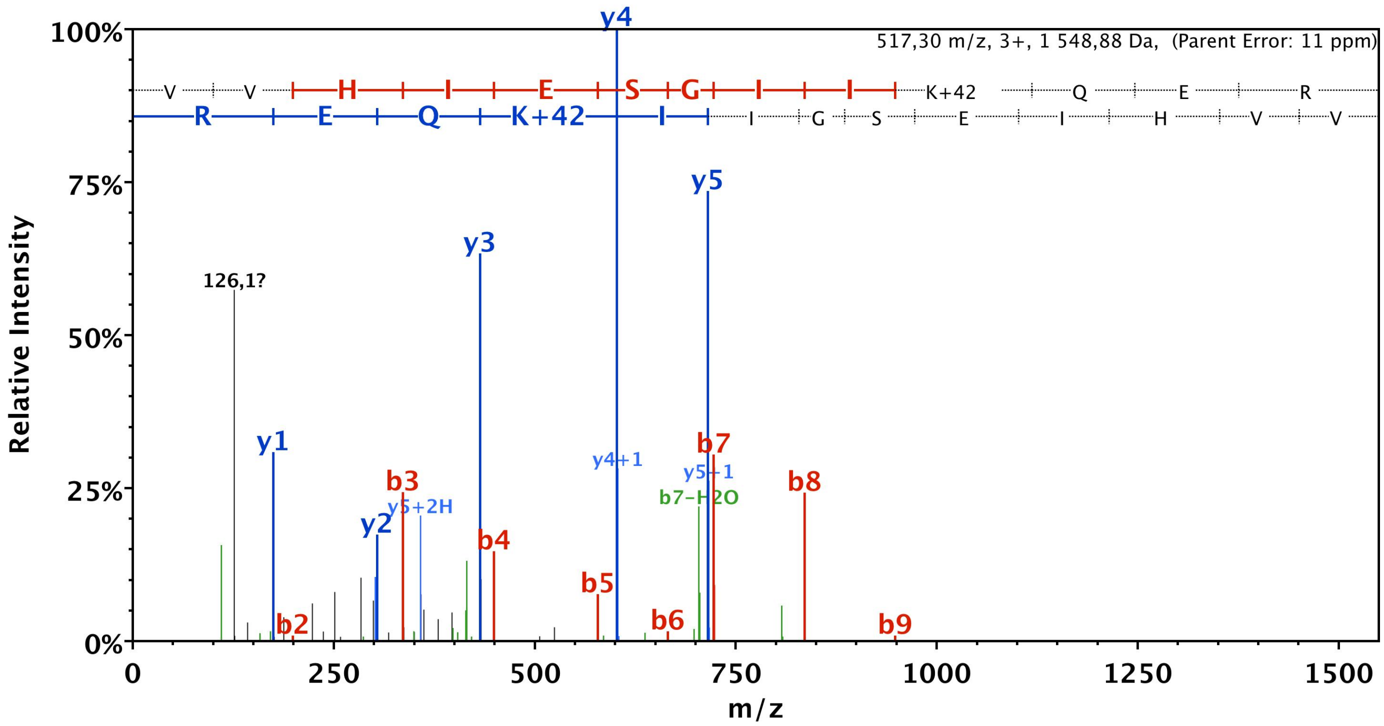

B	B Ions	B+2H	B-NH3	B-H2O	AA	Y Ions	Y+2H	Y-NH3	Y-H2O	Y
1	100,1	50,5			V	1 549,9	775,4	1 532,8	1 531,9	13
2	199,1	100,1			V	1 450,8	725,9	1 433,8	1 432,8	12
3	336,2	168,6			H	1 351,7	676,4	1 334,7	1 333,7	11
4	449,3	225,1			I	1 214,7	607,8	1 197,6	1 196,7	10
5	578,3	289,7		560,3	E	1 101,6	551,3	1 084,6	1 083,6	9
6	665,4	333,2		647,4	S	972,5	486,8	955,5	954,5	8
7	722,4	361,7		704,4	G	885,5	443,3	868,5	867,5	7
8	835,5	418,2		817,5	I	828,5	414,8	811,5	810,5	6
9	948,6	474,8		930,5	I	715,4	358,2	698,4	697,4	5
10	1 118,7	559,8	1 101,6	1 100,6	K+42	602,3	301,7	585,3	584,3	4
11	1 246,7	623,9	1 229,7	1 228,7	Q	432,2	216,6	415,2	414,2	3
12	1 375,8	688,4	1 358,7	1 357,7	E	304,2	152,6	287,1	286,2	2
13	1 549,9	775,4	1 532,8	1 531,9	R	175,1	88,1	158,1		1

#2 K128

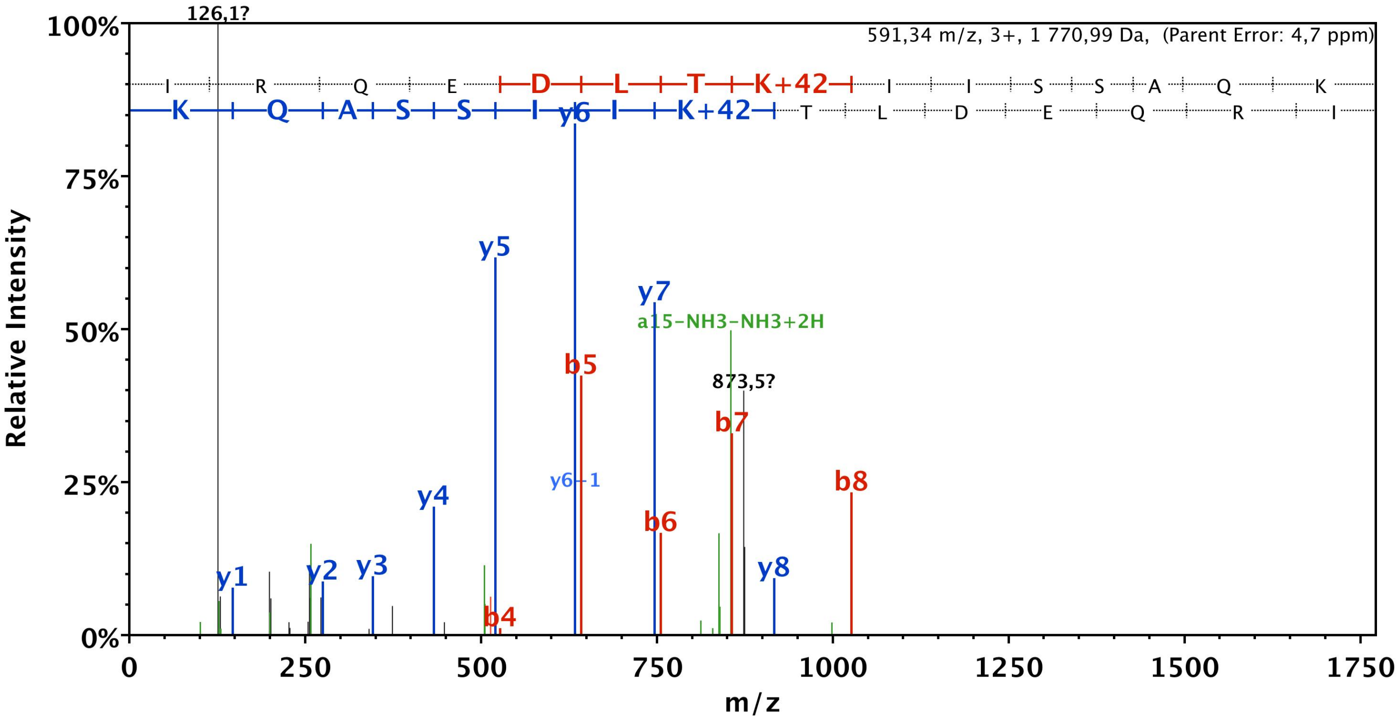

B	B Ions	B+2H	B-NH3	B-H2O	AA	Y Ions	Y+2H	Y-NH3	Y-H2O	Y
1	114,1	57,5			I	1 772,0	886,5	1 755,0	1 754,0	15
2	270,2	135,6	253,2		R	1 658,9	830,0	1 641,9	1 640,9	14
3	398,3	199,6	381,2		Q	1 502,8	751,9	1 485,8	1 484,8	13
4	527,3	264,2	510,3	509,3	E	1 374,7	687,9	1 357,7	1 356,7	12
5	642,3	321,7	625,3	624,3	D	1 245,7	623,4	1 228,7	1 227,7	11
6	755,4	378,2	738,4	737,4	L	1 130,7	565,8	1 113,7	1 112,7	10
7	856,5	428,7	839,4	838,4	T	1 017,6	509,3	1 000,6	999,6	9
8	1 026,6	513,8	1 009,5	1 008,5	K+42	916,5	458,8	899,5	898,5	8
9	1 139,6	570,3	1 122,6	1 121,6	I	746,4	373,7	729,4	728,4	7
10	1 252,7	626,9	1 235,7	1 234,7	I	633,4	317,2	616,3	615,3	6
11	1 339,8	670,4	1 322,7	1 321,7	S	520,3	260,6	503,2	502,3	5
12	1 426,8	713,9	1 409,8	1 408,8	S	433,2	217,1	416,2	415,2	4
13	1 497,8	749,4	1 480,8	1 479,8	A	346,2	173,6	329,2		3
14	1 625,9	813,4	1 608,9	1 607,9	Q	275,2	138,1	258,1		2
15	1 772,0	886,5	1 755,0	1 754,0	K	147,1	74,1	130,1		1

#3 K128

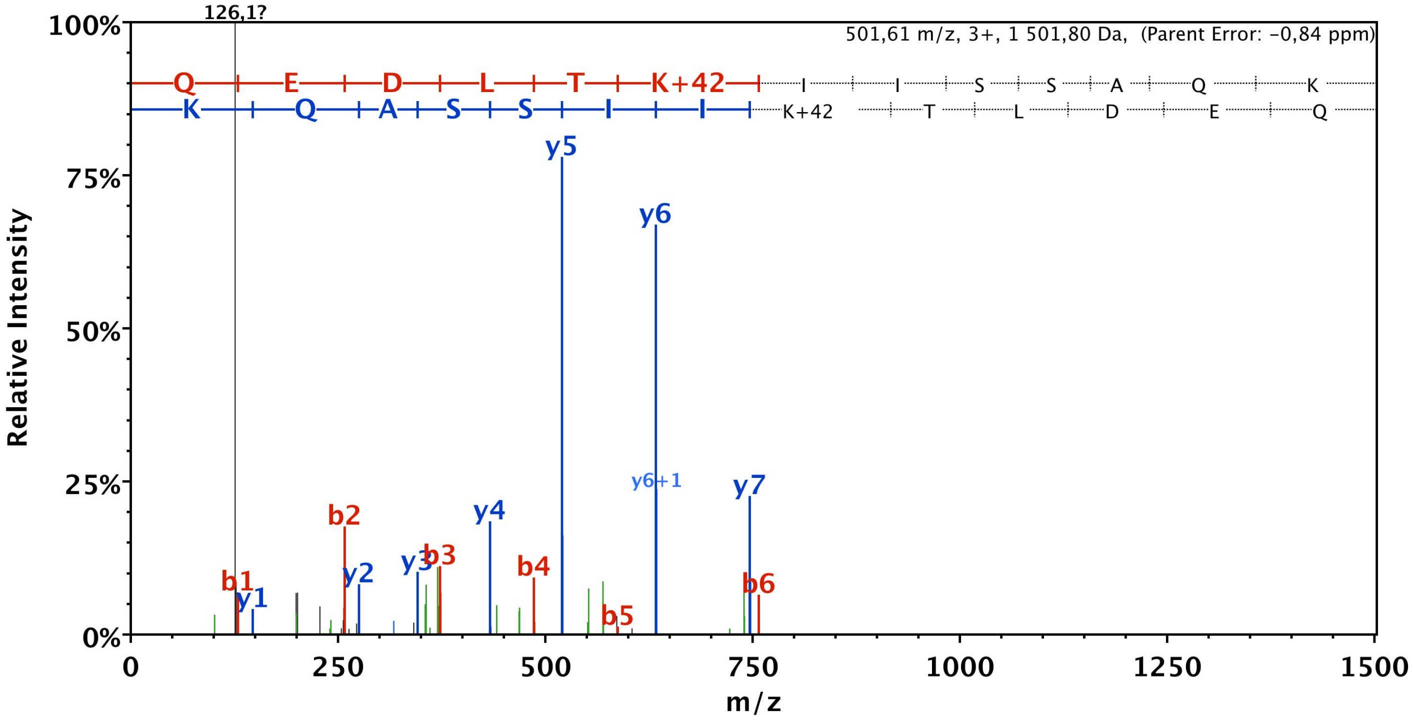

B	B Ions	B+2H	B-NH3	B-H2O	AA	Y Ions	Y+2H	Y-NH3	Y-H2O	Y
1	129,1	65,0	112,0		Q	1 502,8	751,9	1 485,8	1 484,8	13
2	258,1	129,6	241,1	240,1	E	1 374,7	687,9	1 357,7	1 356,7	12
3	373,1	187,1	356,1	355,1	D	1 245,7	623,4	1 228,7	1 227,7	11
4	486,2	243,6	469,2	468,2	L	1 130,7	565,8	1 113,7	1 112,7	10
5	587,3	294,1	570,2	569,3	T	1 017,6	509,3	1 000,6	999,6	9
6	757,4	379,2	740,3	739,4	K+42	916,5	458,8	899,5	898,5	8
7	870,5	435,7	853,4	852,4	I	746,4	373,7	729,4	728,4	7
8	983,5	492,3	966,5	965,5	I	633,4	317,2	616,3	615,3	6
9	1 070,6	535,8	1 053,5	1 052,6	S	520,3	260,6	503,2	502,3	5
10	1 157,6	579,3	1 140,6	1 139,6	S	433,2	217,1	416,2	415,2	4
11	1 228,6	614,8	1 211,6	1 210,6	A	346,2	173,6	329,2		3
12	1 356,7	678,9	1 339,7	1 338,7	Q	275,2	138,1	258,1		2
13	1 502,8	751,9	1 485,8	1 484,8	K	147,1	74,1	130,1		1

#4 K128

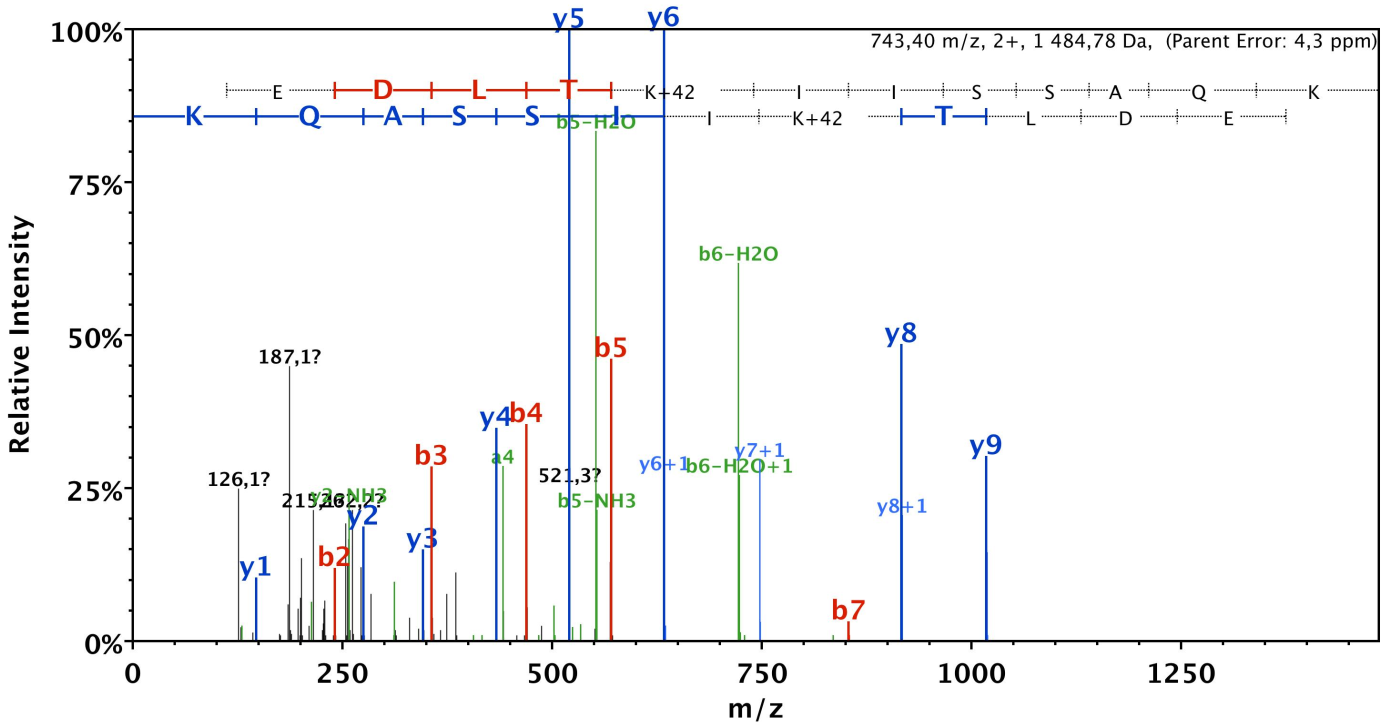

B	B Ions	B+2H	B-NH3	B-H2O	AA	Y Ions	Y+2H	Y-NH3	Y-H2O	Y
1	112,0		95,0		Q-17	1 485,8	743,4	1 468,8	1 467,8	13
2	241,1		224,1	223,1	E	1 374,7	687,9	1 357,7	1 356,7	12
3	356,1		339,1	338,1	D	1 245,7	623,4	1 228,7	1 227,7	11
4	469,2		452,2	451,2	L	1 130,7	565,8	1 113,7	1 112,7	10
5	570,2		553,2	552,2	T	1 017,6	509,3	1 000,6	999,6	9
6	740,3	370,7	723,3	722,3	K+42	916,5	458,8	899,5	898,5	8
7	853,4	427,2	836,4	835,4	I	746,4	373,7	729,4	728,4	7
8	966,5	483,8	949,5	948,5	I	633,4	317,2	616,3	615,3	6
9	1 053,5	527,3	1 036,5	1 035,5	S	520,3		503,2	502,3	5
10	1 140,6	570,8	1 123,6	1 122,6	S	433,2		416,2	415,2	4
11	1 211,6	606,3	1 194,6	1 193,6	A	346,2		329,2		3
12	1 339,7	670,3	1 322,6	1 321,7	Q	275,2		258,1		2
13	1 485,8	743,4	1 468,8	1 467,8	K	147,1		130,1		1

#5 K128/135

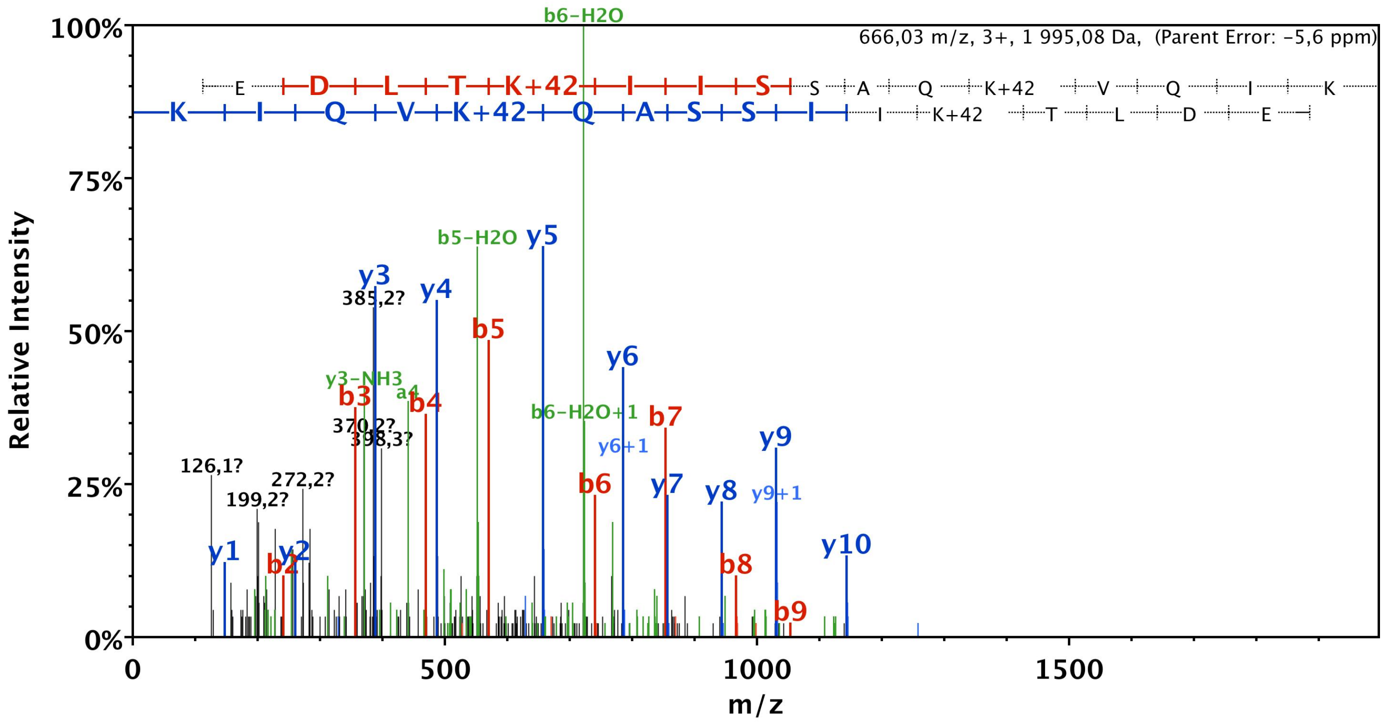

B	B Ions	B+2H	B-NH3	B-H2O	AA	Y Ions	Y+2H	Y-NH3	Y-H2O	Y
1	112,0	56,5	95,0		Q-17	1 996,1	998,6	1 979,1	1 978,1	17
2	241,1	121,0	224,1	223,1	E	1 885,1	943,0	1 868,0	1 867,1	16
3	356,1	178,6	339,1	338,1	D	1 756,0	878,5	1 739,0	1 738,0	15
4	469,2	235,1	452,2	451,2	L	1 641,0	821,0	1 624,0	1 623,0	14
5	570,2	285,6	553,2	552,2	T	1 527,9	764,5	1 510,9	1 509,9	13
6	740,3	370,7	723,3	722,3	K+42	1 426,9	713,9	1 409,8	1 408,9	12
7	853,4	427,2	836,4	835,4	I	1 256,8	628,9	1 239,7	1 238,7	11
8	966,5	483,8	949,5	948,5	I	1 143,7	572,3	1 126,6	1 125,7	10
9	1 053,5	527,3	1 036,5	1 035,5	S	1 030,6	515,8	1 013,6	1 012,6	9
10	1 140,6	570,8	1 123,6	1 122,6	S	943,6	472,3	926,5	925,5	8
11	1 211,6	606,3	1 194,6	1 193,6	A	856,5	428,8	839,5		7
12	1 339,7	670,3	1 322,6	1 321,7	Q	785,5	393,2	768,5		6
13	1 509,8	755,4	1 492,8	1 491,8	K+42	657,4	329,2	640,4		5
14	1 608,8	804,9	1 591,8	1 590,8	V	487,3	244,2	470,3		4
15	1 736,9	869,0	1 719,9	1 718,9	Q	388,3	194,6	371,2		3
16	1 850,0	925,5	1 833,0	1 832,0	I	260,2	130,6	243,2		2
17	1 996,1	998,6	1 979,1	1 978,1	K	147,1	74,1	130,1		1

#6 K128/135

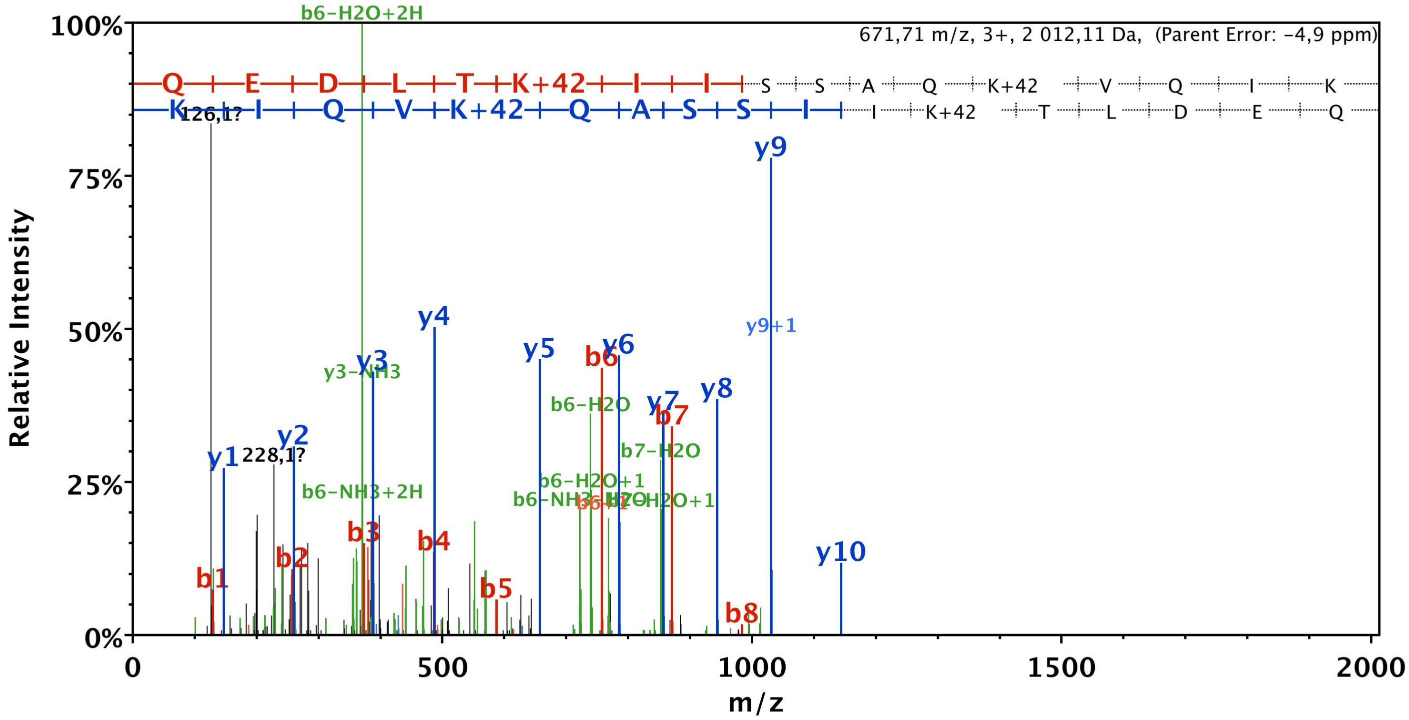

B	B Ions	B+2H	B-NH3	B-H2O	AA	Y Ions	Y+2H	Y-NH3	Y-H2O	Y
1	129,1	65,0	112,0		Q	2 013,1	1 007,1	1 996,1	1 995,1	17
2	258,1	129,6	241,1	240,1	E	1 885,1	943,0	1 868,0	1 867,1	16
3	373,1	187,1	356,1	355,1	D	1 756,0	878,5	1 739,0	1 738,0	15
4	486,2	243,6	469,2	468,2	L	1 641,0	821,0	1 624,0	1 623,0	14
5	587,3	294,1	570,2	569,3	T	1 527,9	764,5	1 510,9	1 509,9	13
6	757,4	379,2	740,3	739,4	K+42	1 426,9	713,9	1 409,8	1 408,9	12
7	870,5	435,7	853,4	852,4	I	1 256,8	628,9	1 239,7	1 238,7	11
8	983,5	492,3	966,5	965,5	I	1 143,7	572,3	1 126,6	1 125,7	10
9	1 070,6	535,8	1 053,5	1 052,6	S	1 030,6	515,8	1 013,6	1 012,6	9
10	1 157,6	579,3	1 140,6	1 139,6	S	943,6	472,3	926,5	925,5	8
11	1 228,6	614,8	1 211,6	1 210,6	A	856,5	428,8	839,5		7
12	1 356,7	678,9	1 339,7	1 338,7	Q	785,5	393,2	768,5		6
13	1 526,8	763,9	1 509,8	1 508,8	K+42	657,4	329,2	640,4		5
14	1 625,9	813,4	1 608,8	1 607,9	V	487,3	244,2	470,3		4
15	1 753,9	877,5	1 736,9	1 735,9	Q	388,3	194,6	371,2		3
16	1 867,0	934,0	1 850,0	1 849,0	I	260,2	130,6	243,2		2
17	2 013,1	1 007,1	1 996,1	1 995,1	K	147,1	74,1	130,1		1

#7 K135

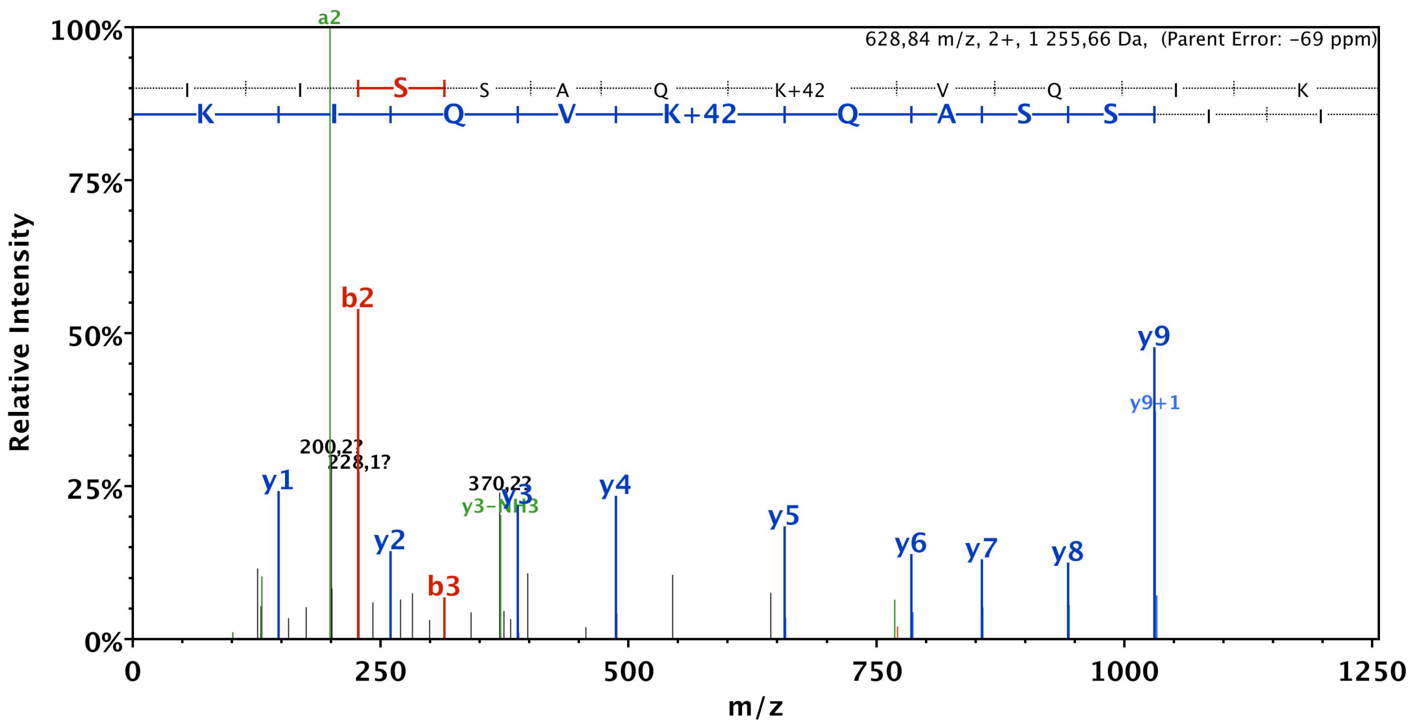

B	B Ions	B+2H	B-NH3	B-H2O	AA	Y Ions	Y+2H	Y-NH3	Y-H2O	Y
1	114,1				I	1 256,8	628,9	1 239,7	1 238,7	11
2	227,2				I	1 143,7	572,3	1 126,6	1 125,7	10
3	314,2			296,2	S	1 030,6	515,8	1 013,6	1 012,6	9
4	401,2			383,2	S	943,6	472,3	926,5	925,5	8
5	472,3			454,3	A	856,5	428,8	839,5		7
6	600,3	300,7	583,3	582,3	Q	785,5	393,2	768,5		6
7	770,4	385,7	753,4	752,4	K+42	657,4	329,2	640,4		5
8	869,5	435,3	852,5	851,5	V	487,3		470,3		4
9	997,6	499,3	980,5	979,6	Q	388,3		371,2		3
10	1 110,7	555,8	1 093,6	1 092,6	I	260,2		243,2		2
11	1 256,8	628,9	1 239,7	1 238,7	K	147,1		130,1		1

#8 K197

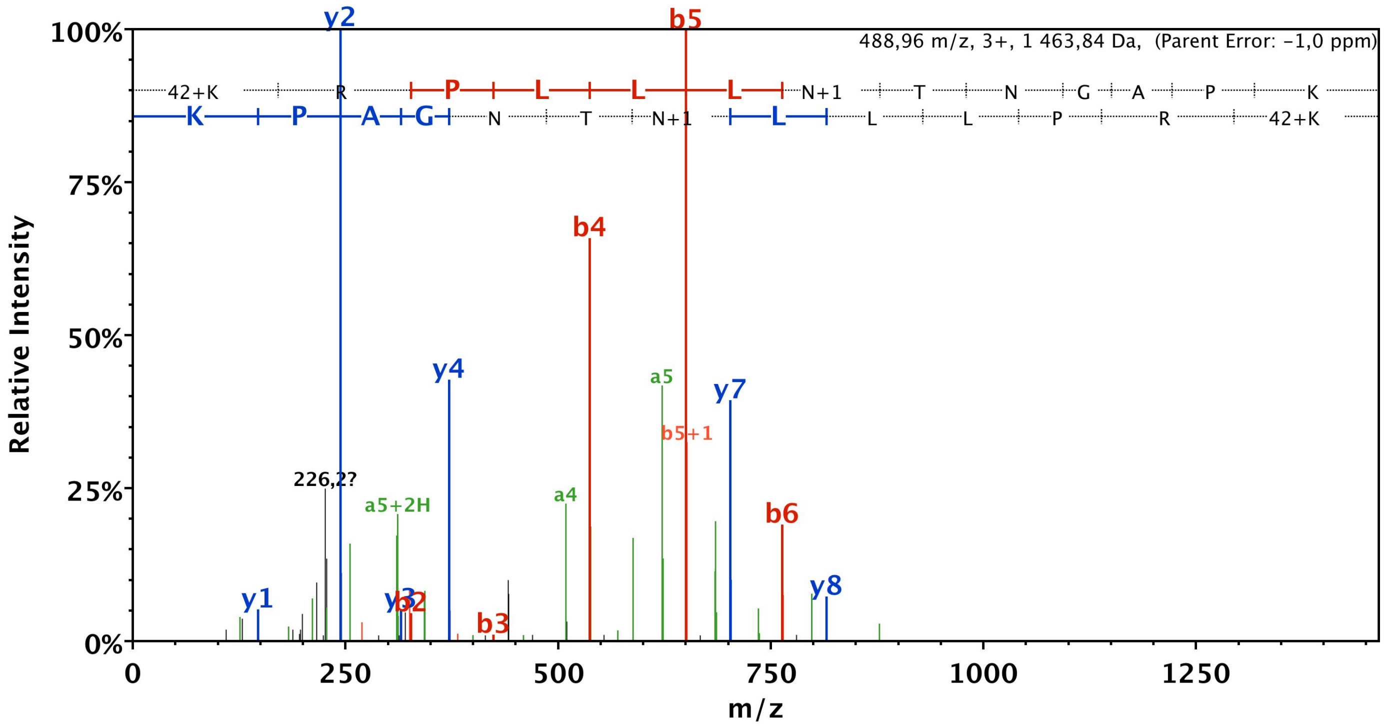

B	B Ions	B+2H	B-NH3	B-H2O	AA	Y Ions	Y+2H	Y-NH3	Y-H2O	Y
1	171,1	86,1	154,1		K+42	1 464,9	732,9	1 447,8	1 446,8	13
2	327,2	164,1	310,2		R	1 294,7	647,9	1 277,7	1 276,7	12
3	424,3	212,6	407,2		P	1 138,6	569,8	1 121,6	1 120,6	11
4	537,4	269,2	520,3		L	1 041,6	521,3	1 024,6	1 023,6	10
5	650,4	325,7	633,4		L	928,5	464,8	911,5	910,5	9
6	763,5	382,3	746,5		L	815,4	408,2	798,4	797,4	8
7	878,5	439,8	861,5		N+1	702,3	351,7	685,3	684,3	7
8	979,6	490,3	962,6	961,6	T	587,3	294,2	570,3	569,3	6
9	1 093,6	547,3	1 076,6	1 075,6	N	486,3	243,6	469,2		5
10	1 150,7	575,8	1 133,6	1 132,6	G	372,2	186,6	355,2		4
11	1 221,7	611,4	1 204,7	1 203,7	A	315,2	158,1	298,2		3
12	1 318,7	659,9	1 301,7	1 300,7	P	244,2	122,6	227,1		2
13	1 464,9	732,9	1 447,8	1 446,8	K	147,1	74,1	130,1		1

#9 K209

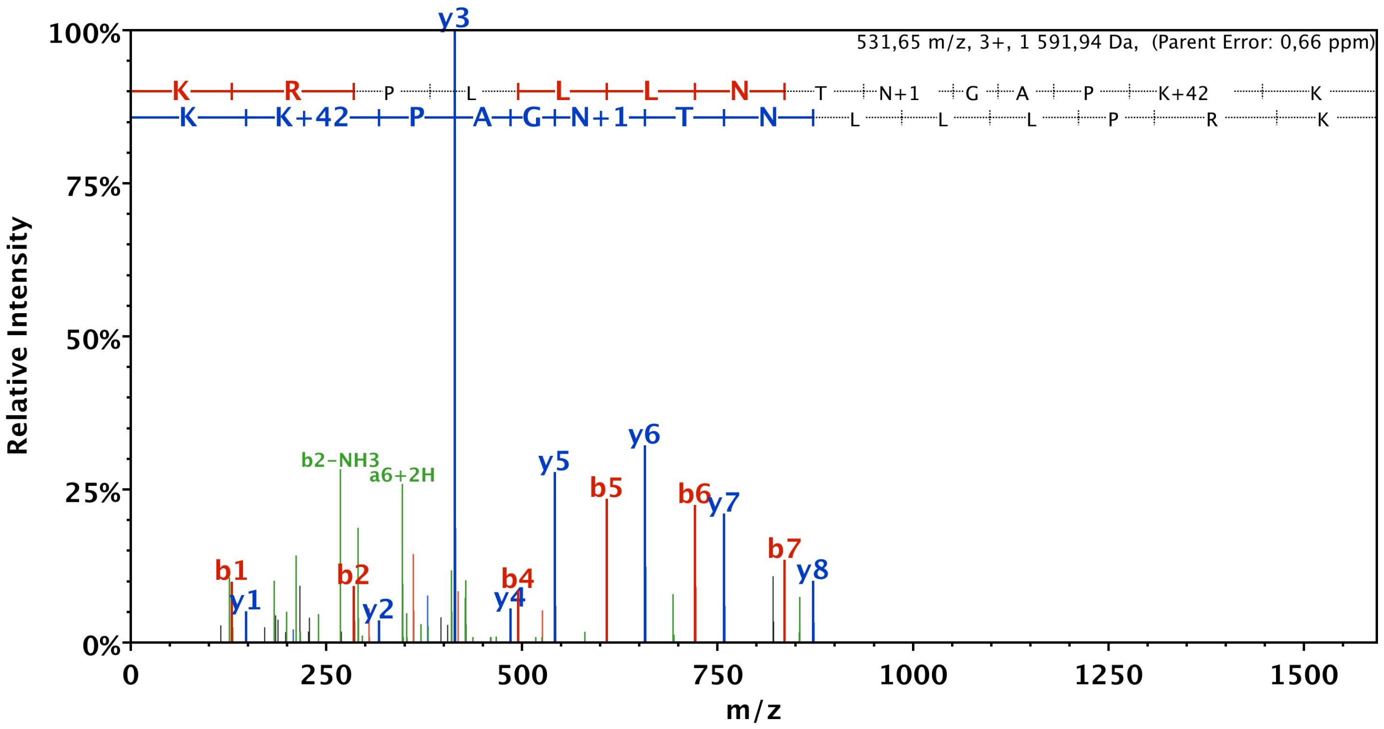

B	B Ions	B+2H	B-NH3	B-H2O	AA	Y Ions	Y+2H	Y-NH3	Y-H2O	Y
1	129,1	65,1	112,1		K	1 592,9	797,0	1 575,9	1 574,9	14
2	285,2	143,1	268,2		R	1 464,9	732,9	1 447,8	1 446,8	13
3	382,3	191,6	365,2		P	1 308,8	654,9	1 291,7	1 290,7	12
4	495,3	248,2	478,3		L	1 211,7	606,4	1 194,7	1 193,7	11
5	608,4	304,7	591,4		L	1 098,6	549,8	1 081,6	1 080,6	10
6	721,5	361,3	704,5		L	985,5	493,3	968,5	967,5	9
7	835,6	418,3	818,5		N	872,4	436,7	855,4	854,4	8
8	936,6	468,8	919,6	918,6	T	758,4	379,7	741,4	740,4	7
9	1 051,6	526,3	1 034,6	1 033,6	N+1	657,4	329,2	640,3		6
10	1 108,6	554,8	1 091,6	1 090,6	G	542,3	271,7	525,3		5
11	1 179,7	590,3	1 162,7	1 161,7	A	485,3	243,2	468,3		4
12	1 276,7	638,9	1 259,7	1 258,7	P	414,3	207,6	397,2		3
13	1 446,8	723,9	1 429,8	1 428,8	K+42	317,2	159,1	300,2		2
14	1 592,9	797,0	1 575,9	1 574,9	K	147,1	74,1	130,1		1

#10 K209/210

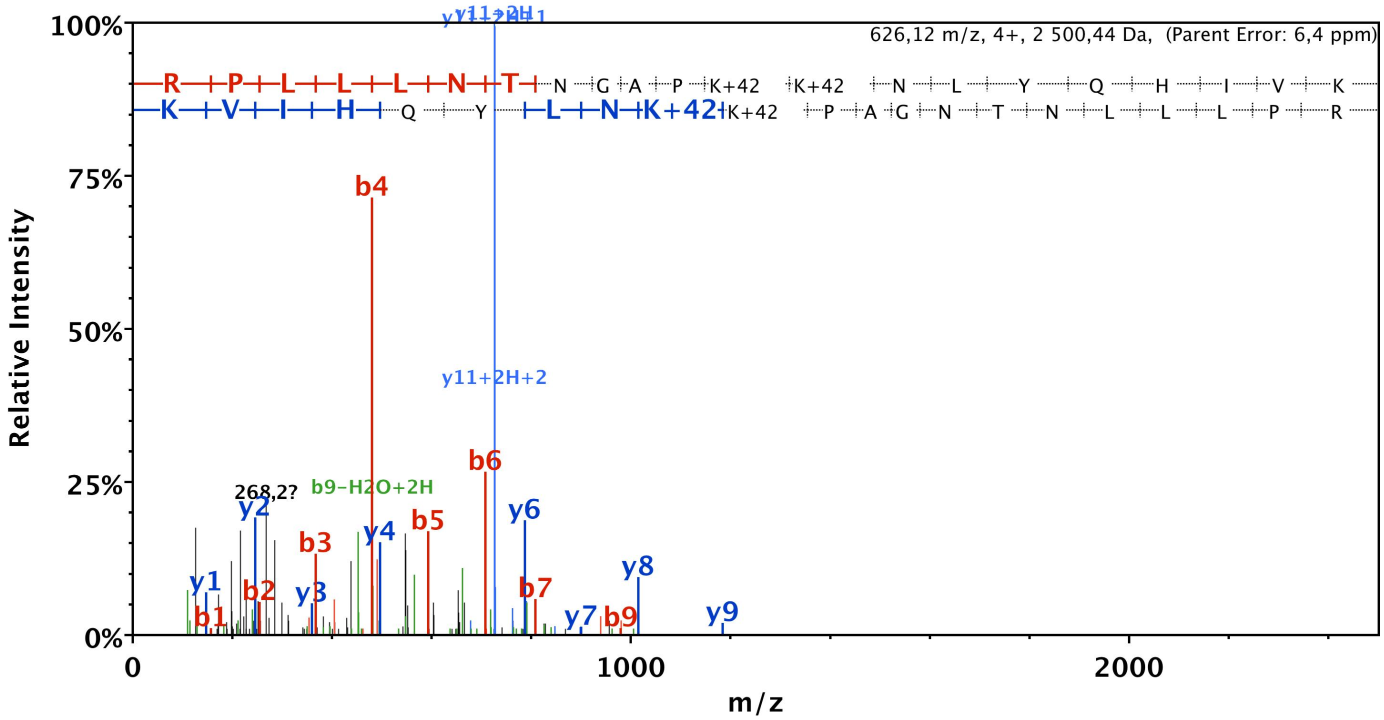

B	B Ions	B+2H	B-NH3	B-H2O	AA	Y Ions	Y+2H	Y-NH3	Y-H2O	Y
1	157,1	79,1	140,1		R	2 501,4	1 251,2	2 484,4	2 483,4	21
2	254,2	127,6	237,1		P	2 345,3	1 173,2	2 328,3	2 327,3	20
3	367,2	184,1	350,2		L	2 248,3	1 124,6	2 231,3	2 230,3	19
4	480,3	240,7	463,3		L	2 135,2	1 068,1	2 118,2	2 117,2	18
5	593,4	297,2	576,4		L	2 022,1	1 011,6	2 005,1	2 004,1	17
6	707,5	354,2	690,4		N	1 909,0	955,0	1 892,0	1 891,0	16
7	808,5	404,8	791,5	790,5	T	1 795,0	898,0	1 778,0	1 777,0	15
8	922,5	461,8	905,5	904,5	N	1 693,9	847,5	1 676,9		14
9	979,6	490,3	962,5	961,6	G	1 579,9	790,5	1 562,9		13
10	1 050,6	525,8	1 033,6	1 032,6	A	1 522,9	761,9	1 505,8		12
11	1 147,7	574,3	1 130,6	1 129,6	P	1 451,8	726,4	1 434,8		11
12	1 317,8	659,4	1 300,7	1 299,8	K+42	1 354,8	677,9	1 337,8		10
13	1 487,9	744,4	1 470,8	1 469,9	K+42	1 184,7	592,8	1 167,7		9
14	1 601,9	801,5	1 584,9	1 583,9	N	1 014,6	507,8	997,5		8
15	1 715,0	858,0	1 698,0	1 697,0	L	900,5	450,8	883,5		7
16	1 878,1	939,5	1 861,0	1 860,0	Y	787,4	394,2	770,4		6
17	2 006,1	1 003,6	1 989,1	1 988,1	Q	624,4	312,7	607,4		5
18	2 143,2	1 072,1	2 126,2	2 125,2	H	496,3	248,7	479,3		4
19	2 256,3	1 128,6	2 239,2	2 238,3	I	359,3	180,1	342,2		3
20	2 355,3	1 178,2	2 338,3	2 337,3	V	246,2	123,6	229,2		2
21	2 501,4	1 251,2	2 484,4	2 483,4	K	147,1	74,1	130,1		1

#11 K209/210

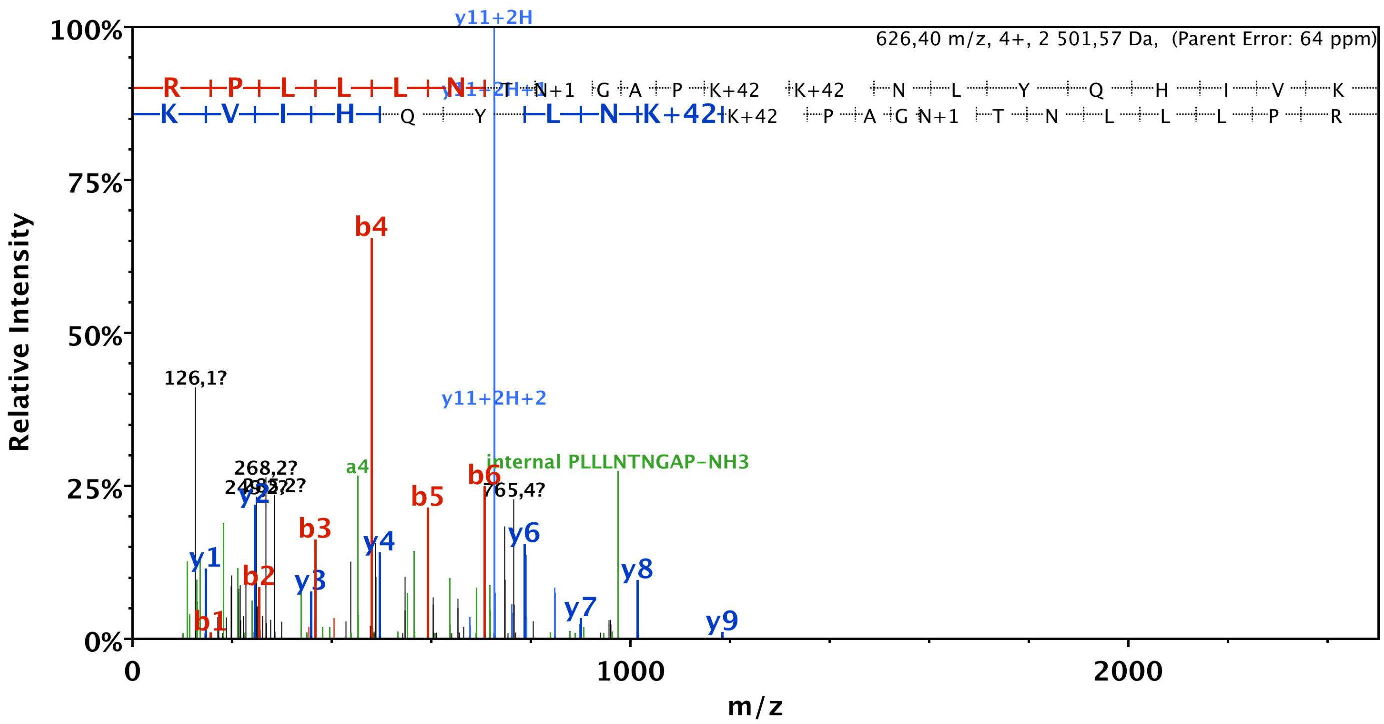

B	B Ions	B+2H	B-NH3	B-H2O	AA	Y Ions	Y+2H	Y-NH3	Y-H2O	Y
1	157,1	79,1	140,1		R	2 502,4	1 251,7	2 485,4	2 484,4	21
2	254,2	127,6	237,1		P	2 346,3	1 173,7	2 329,3	2 328,3	20
3	367,2	184,1	350,2		L	2 249,3	1 125,1	2 232,2	2 231,3	19
4	480,3	240,7	463,3		L	2 136,2	1 068,6	2 119,2	2 118,2	18
5	593,4	297,2	576,4		L	2 023,1	1 012,1	2 006,1	2 005,1	17
6	707,5	354,2	690,4		N	1 910,0	955,5	1 893,0	1 892,0	16
7	808,5	404,8	791,5	790,5	T	1 796,0	898,5	1 778,9	1 778,0	15
8	923,5	462,3	906,5	905,5	N+1	1 694,9	848,0	1 677,9		14
9	980,6	490,8	963,5	962,5	G	1 579,9	790,5	1 562,9		13
10	1 051,6	526,3	1 034,6	1 033,6	A	1 522,9	761,9	1 505,8		12
11	1 148,6	574,8	1 131,6	1 130,6	P	1 451,8	726,4	1 434,8		11
12	1 318,7	659,9	1 301,7	1 300,7	K+42	1 354,8	677,9	1 337,8		10
13	1 488,9	744,9	1 471,8	1 470,8	K+42	1 184,7	592,8	1 167,7		9
14	1 602,9	802,0	1 585,9	1 584,9	N	1 014,6	507,8	997,5		8
15	1 716,0	858,5	1 699,0	1 698,0	L	900,5	450,8	883,5		7
16	1 879,0	940,0	1 862,0	1 861,0	Y	787,4	394,2	770,4		6
17	2 007,1	1 004,1	1 990,1	1 989,1	Q	624,4	312,7	607,4		5
18	2 144,2	1 072,6	2 127,1	2 126,2	H	496,3	248,7	479,3		4
19	2 257,2	1 129,1	2 240,2	2 239,2	I	359,3	180,1	342,2		3
20	2 356,3	1 178,7	2 339,3	2 338,3	V	246,2	123,6	229,2		2
21	2 502,4	1 251,7	2 485,4	2 484,4	K	147,1	74,1	130,1		1

#12 K210

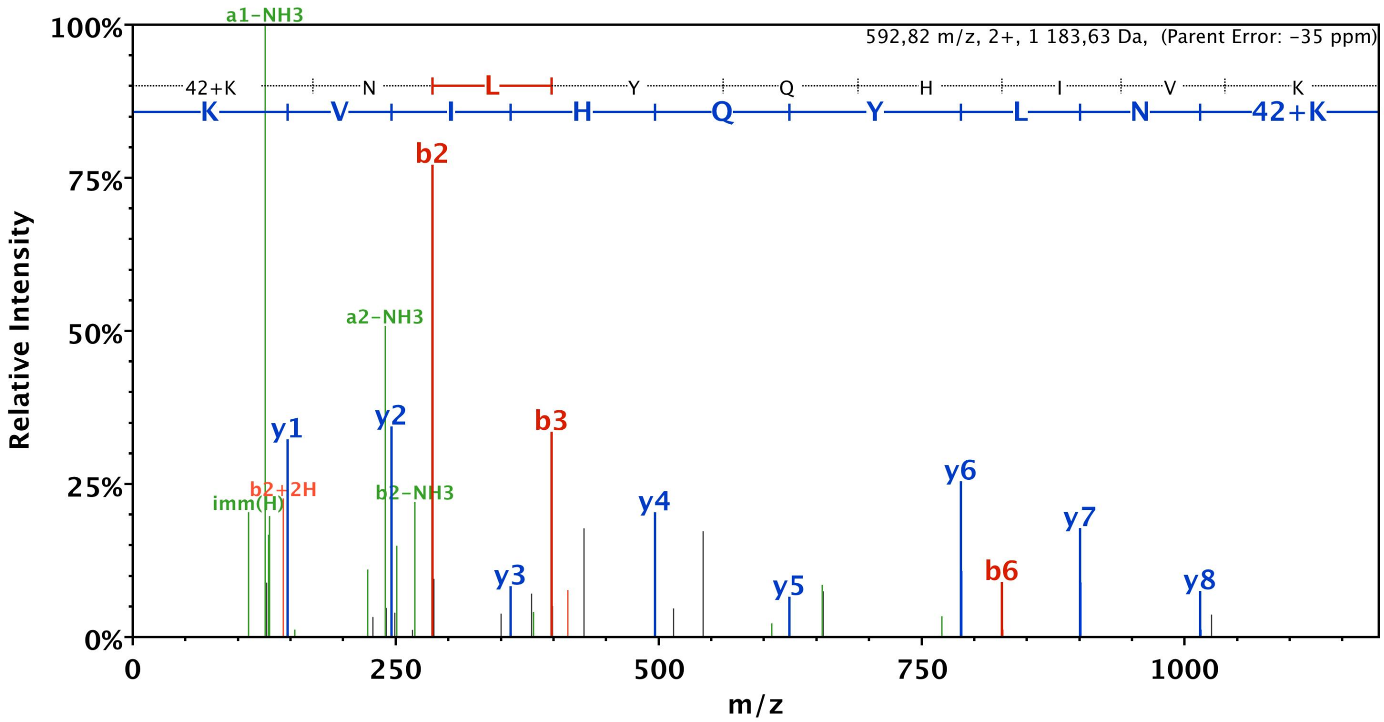

B	B Ions	B+2H	B-NH3	B-H2O	AA	Y Ions	Y+2H	Y-NH3	Y-H2O	Y
1	171,1	86,1	154,1		K+42	1 184,7	592,8	1 167,7	1 166,7	9
2	285,2	143,1	268,1		N	1 014,6	507,8	997,5	996,6	8
3	398,2	199,6	381,2		L	900,5	450,8	883,5	882,5	7
4	561,3	281,2	544,3	543,3	Y	787,4	394,2	770,4	769,4	6
5	689,4	345,2	672,3	671,4	Q	624,4	312,7	607,4	606,4	5
6	826,4	413,7	809,4	808,4	H	496,3	248,7	479,3	478,3	4
7	939,5	470,3	922,5	921,5	I	359,3		342,2		3
8	1 038,6	519,8	1 021,5	1 020,6	V	246,2		229,2		2
9	1 184,7	592,8	1 167,7	1 166,7	K	147,1		130,1		1

#13 K395

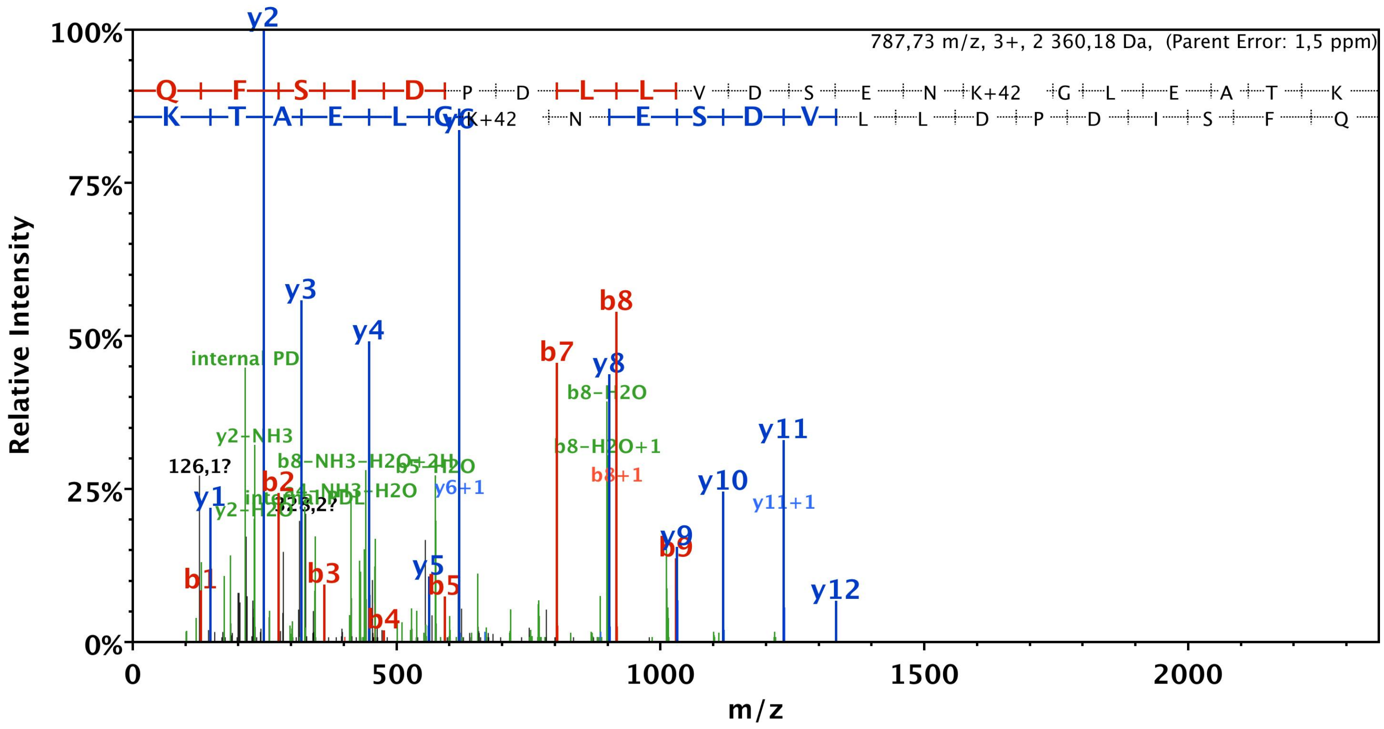

B	B Ions	B+2H	B-NH3	B-H2O	AA	Y Ions	Y+2H	Y-NH3	Y-H2O	Y
1	129,1	65,0	112,0		Q	2 361,2	1 181,1	2 344,2	2 343,2	21
2	276,1	138,6	259,1		F	2 233,1	1 117,1	2 216,1	2 215,1	20
3	363,2	182,1	346,1	345,2	S	2 086,1	1 043,5	2 069,0	2 068,0	19
4	476,3	238,6	459,2	458,2	I	1 999,0	1 000,0	1 982,0	1 981,0	18
5	591,3	296,1	574,3	573,3	D	1 885,9	943,5	1 868,9	1 867,9	17
6	688,3	344,7	671,3	670,3	P	1 770,9	886,0	1 753,9	1 752,9	16
7	803,4	402,2	786,3	785,3	D	1 673,9	837,4	1 656,8	1 655,8	15
8	916,4	458,7	899,4	898,4	L	1 558,8	779,9	1 541,8	1 540,8	14
9	1 029,5	515,3	1 012,5	1 011,5	L	1 445,7	723,4	1 428,7	1 427,7	13
10	1 128,6	564,8	1 111,6	1 110,6	V	1 332,7	666,8	1 315,6	1 314,7	12
11	1 243,6	622,3	1 226,6	1 225,6	D	1 233,6	617,3	1 216,6	1 215,6	11
12	1 330,7	665,8	1 313,6	1 312,6	S	1 118,6	559,8	1 101,5	1 100,6	10
13	1 459,7	730,4	1 442,7	1 441,7	E	1 031,5	516,3	1 014,5	1 013,5	9
14	1 573,7	787,4	1 556,7	1 555,7	N	902,5	451,8	885,5	884,5	8
15	1 743,8	872,4	1 726,8	1 725,8	K+42	788,5	394,7	771,4	770,4	7
16	1 800,9	900,9	1 783,8	1 782,9	G	618,3	309,7	601,3	600,3	6
17	1 913,9	957,5	1 896,9	1 895,9	L	561,3	281,2	544,3	543,3	5
18	2 043,0	1 022,0	2 026,0	2 025,0	E	448,2	224,6	431,2	430,2	4
19	2 114,0	1 057,5	2 097,0	2 096,0	A	319,2	160,1	302,2	301,2	3
20	2 215,1	1 108,0	2 198,0	2 197,1	T	248,2	124,6	231,1	230,1	2
21	2 361,2	1 181,1	2 344,2	2 343,2	K	147,1	74,1	130,1		1

#14 K395

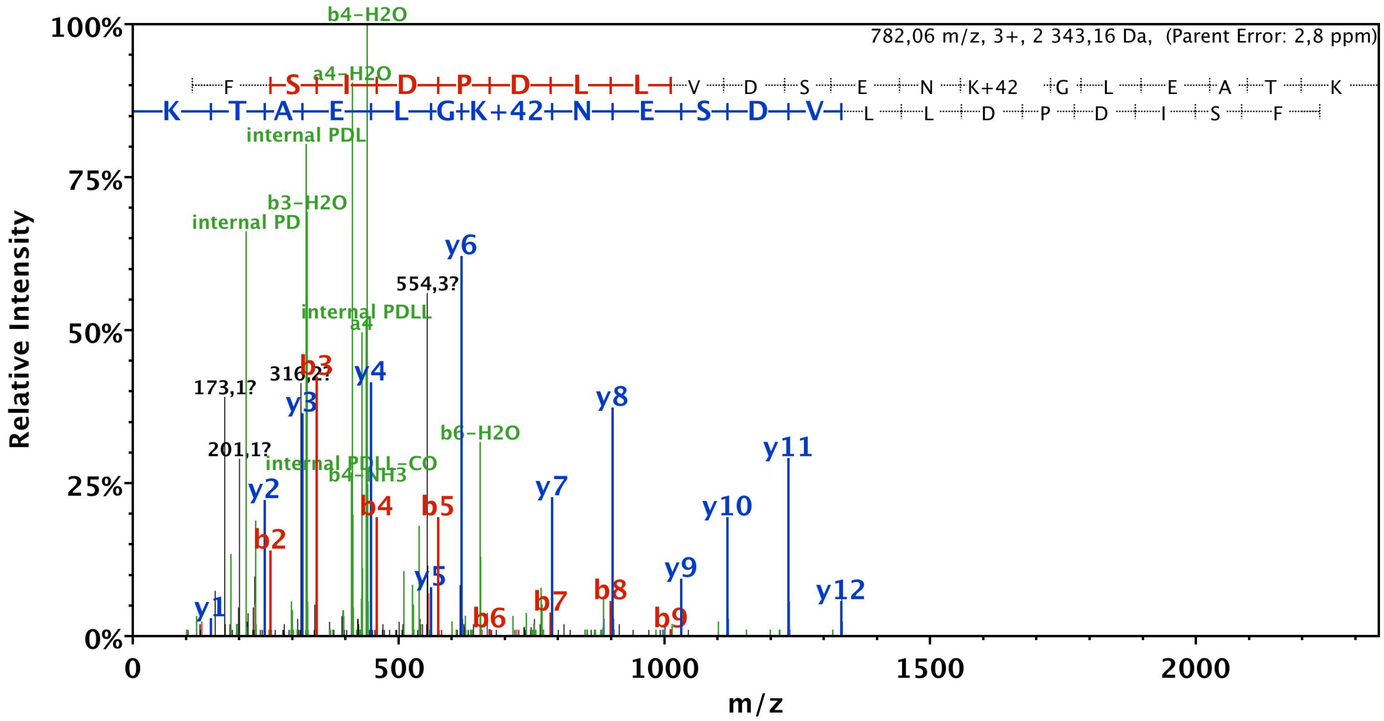

B	B Ions	B+2H	B-NH3	B-H2O	AA	Y Ions	Y+2H	Y-NH3	Y-H2O	Y
1	112,0	56,5	95,0		Q-17	2 344,2	1 172,6	2 327,1	2 326,1	21
2	259,1	130,1	242,1		F	2 233,1	1 117,1	2 216,1	2 215,1	20
3	346,1	173,6	329,1	328,1	S	2 086,1	1 043,5	2 069,0	2 068,0	19
4	459,2	230,1	442,2	441,2	I	1 999,0	1 000,0	1 982,0	1 981,0	18
5	574,3	287,6	557,2	556,2	D	1 885,9	943,5	1 868,9	1 867,9	17
6	671,3	336,2	654,3	653,3	P	1 770,9	886,0	1 753,9	1 752,9	16
7	786,3	393,7	769,3	768,3	D	1 673,9	837,4	1 656,8	1 655,8	15
8	899,4	450,2	882,4	881,4	L	1 558,8	779,9	1 541,8	1 540,8	14
9	1 012,5	506,8	995,5	994,5	L	1 445,7	723,4	1 428,7	1 427,7	13
10	1 111,6	556,3	1 094,5	1 093,6	V	1 332,7	666,8	1 315,6	1 314,7	12
11	1 226,6	613,8	1 209,6	1 208,6	D	1 233,6	617,3	1 216,6	1 215,6	11
12	1 313,6	657,3	1 296,6	1 295,6	S	1 118,6	559,8	1 101,5	1 100,6	10
13	1 442,7	721,8	1 425,6	1 424,7	E	1 031,5	516,3	1 014,5	1 013,5	9
14	1 556,7	778,9	1 539,7	1 538,7	N	902,5	451,8	885,5	884,5	8
15	1 726,8	863,9	1 709,8	1 708,8	K+42	788,5	394,7	771,4	770,4	7
16	1 783,8	892,4	1 766,8	1 765,8	G	618,3	309,7	601,3	600,3	6
17	1 896,9	949,0	1 879,9	1 878,9	L	561,3	281,2	544,3	543,3	5
18	2 026,0	1 013,5	2 008,9	2 008,0	E	448,2	224,6	431,2	430,2	4
19	2 097,0	1 049,0	2 080,0	2 079,0	A	319,2	160,1	302,2	301,2	3
20	2 198,1	1 099,5	2 181,0	2 180,0	T	248,2	124,6	231,1	230,1	2
21	2 344,2	1 172,6	2 327,1	2 326,1	K	147,1	74,1	130,1		1

#15 K401

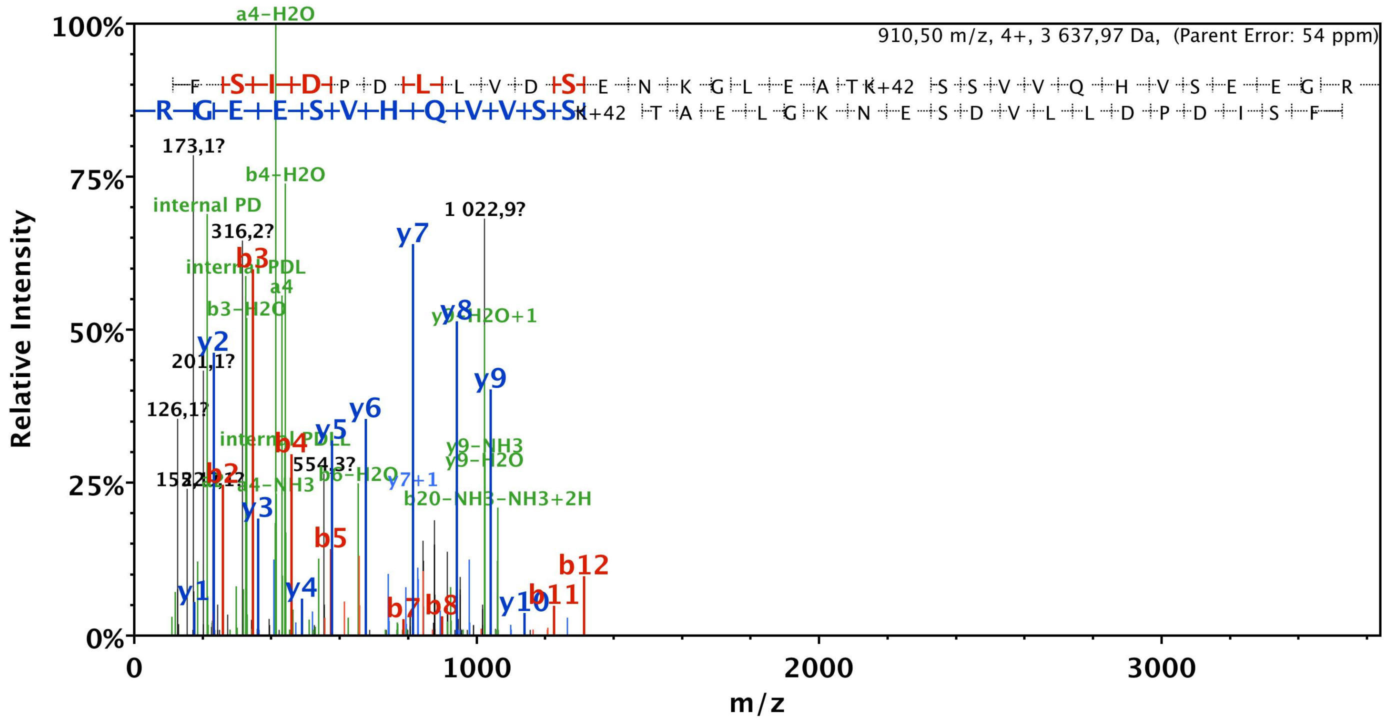

B	B Ions	B+2H	B-NH3	B-H2O	AA	Y Ions	Y+2H	Y-NH3	Y-H2O	Y
1	112,0	56,5	95,0		Q-17	3 638,8	1 819,9	3 621,8	3 620,8	33
2	259,1	130,1	242,1		F	3 527,8	1 764,4	3 510,7	3 509,7	32
3	346,1	173,6	329,1	328,1	S	3 380,7	1 690,8	3 363,7	3 362,7	31
4	459,2	230,1	442,2	441,2	I	3 293,6	1 647,3	3 276,6	3 275,6	30
5	574,3	287,6	557,2	556,2	D	3 180,6	1 590,8	3 163,5	3 162,6	29
6	671,3	336,2	654,3	653,3	P	3 065,5	1 533,3	3 048,5	3 047,5	28
7	786,3	393,7	769,3	768,3	D	2 968,5	1 484,7	2 951,5	2 950,5	27
8	899,4	450,2	882,4	881,4	L	2 853,5	1 427,2	2 836,4	2 835,4	26
9	1 012,5	506,8	995,5	994,5	L	2 740,4	1 370,7	2 723,3	2 722,4	25
10	1 111,6	556,3	1 094,5	1 093,6	V	2 627,3	1 314,1	2 610,3	2 609,3	24
11	1 226,6	613,8	1 209,6	1 208,6	D	2 528,2	1 264,6	2 511,2	2 510,2	23
12	1 313,6	657,3	1 296,6	1 295,6	S	2 413,2	1 207,1	2 396,2	2 395,2	22
13	1 442,7	721,8	1 425,6	1 424,7	E	2 326,2	1 163,6	2 309,1	2 308,2	21
14	1 556,7	778,9	1 539,7	1 538,7	N	2 197,1	1 099,1	2 180,1	2 179,1	20
15	1 684,8	842,9	1 667,8	1 666,8	K	2 083,1	1 042,0	2 066,1	2 065,1	19
16	1 741,8	871,4	1 724,8	1 723,8	G	1 955,0	978,0	1 938,0	1 937,0	18
17	1 854,9	928,0	1 837,9	1 836,9	L	1 898,0	949,5	1 880,9	1 880,0	17
18	1 984,0	992,5	1 966,9	1 965,9	E	1 784,9	892,9	1 767,9	1 766,9	16
19	2 055,0	1 028,0	2 038,0	2 037,0	A	1 655,8	828,4	1 638,8	1 637,8	15
20	2 156,0	1 078,5	2 139,0	2 138,0	T	1 584,8	792,9	1 567,8	1 566,8	14
21	2 326,1	1 163,6	2 309,1	2 308,1	K+42	1 483,7	742,4	1 466,7	1 465,7	13
22	2 413,2	1 207,1	2 396,2	2 395,2	S	1 313,6	657,3	1 296,6	1 295,6	12
23	2 500,2	1 250,6	2 483,2	2 482,2	S	1 226,6	613,8	1 209,6	1 208,6	11
24	2 599,3	1 300,1	2 582,3	2 581,3	V	1 139,6	570,3	1 122,6	1 121,6	10
25	2 698,3	1 349,7	2 681,3	2 680,3	V	1 040,5	520,8	1 023,5	1 022,5	9
26	2 826,4	1 413,7	2 809,4	2 808,4	Q	941,4	471,2	924,4	923,4	8
27	2 963,5	1 482,2	2 946,4	2 945,5	H	813,4	407,2	796,4	795,4	7
28	3 062,5	1 531,8	3 045,5	3 044,5	V	676,3	338,7	659,3	658,3	6
29	3 149,6	1 575,3	3 132,5	3 131,6	S	577,3	289,1	560,2	559,2	5
30	3 278,6	1 639,8	3 261,6	3 260,6	E	490,2	245,6	473,2	472,2	4
31	3 407,6	1 704,3	3 390,6	3 389,6	E	361,2	181,1	344,2	343,2	3
32	3 464,7	1 732,8	3 447,6	3 446,7	G	232,1	116,6	215,1		2
33	3 638,8	1 819,9	3 621,8	3 620,8	R	175,1	88,1	158,1		1

#16 K401

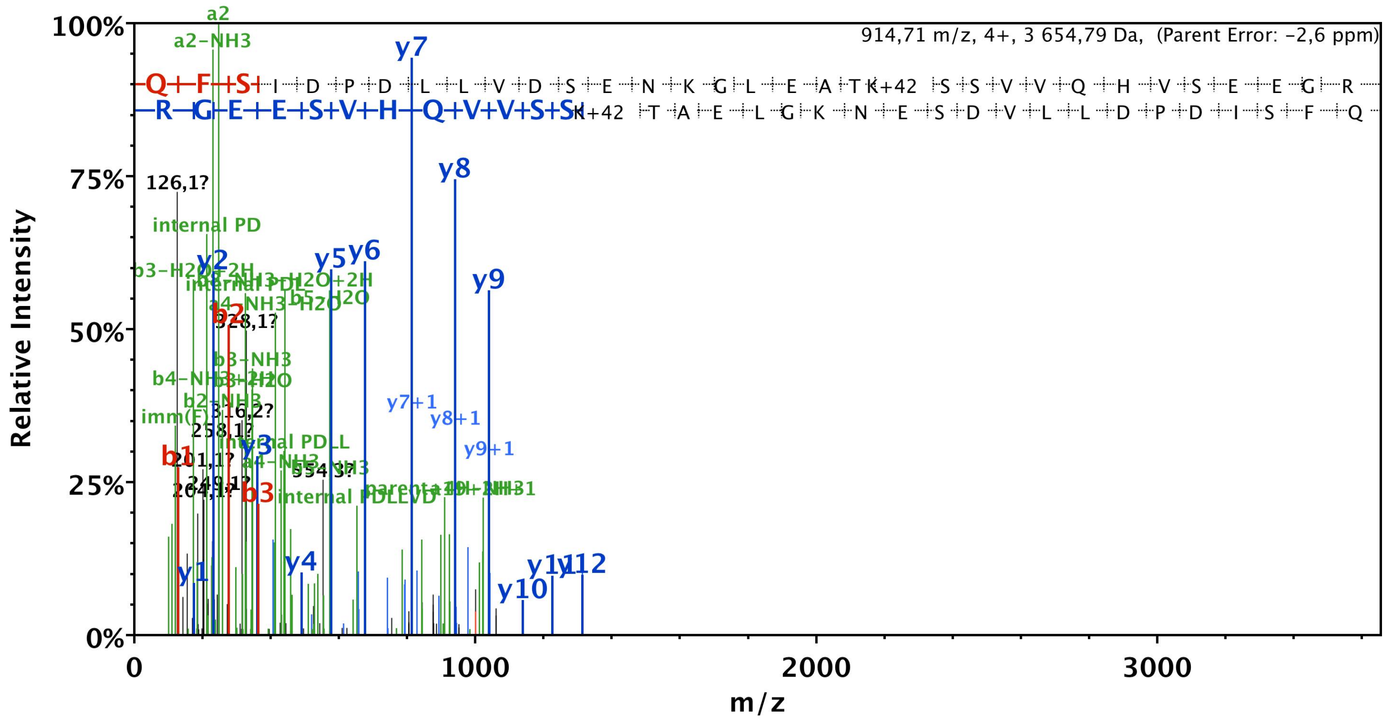

B	B Ions	B+2H	B-NH3	B-H2O	AA	Y Ions	Y+2H	Y-NH3	Y-H2O	Y
1	129,1	65,0	112,0		Q	3 655,8	1 828,4	3 638,8	3 637,8	33
2	276,1	138,6	259,1		F	3 527,8	1 764,4	3 510,7	3 509,7	32
3	363,2	182,1	346,1	345,2	S	3 380,7	1 690,8	3 363,7	3 362,7	31
4	476,3	238,6	459,2	458,2	I	3 293,6	1 647,3	3 276,6	3 275,6	30
5	591,3	296,1	574,3	573,3	D	3 180,6	1 590,8	3 163,5	3 162,6	29
6	688,3	344,7	671,3	670,3	P	3 065,5	1 533,3	3 048,5	3 047,5	28
7	803,4	402,2	786,3	785,3	D	2 968,5	1 484,7	2 951,5	2 950,5	27
8	916,4	458,7	899,4	898,4	L	2 853,5	1 427,2	2 836,4	2 835,4	26
9	1 029,5	515,3	1 012,5	1 011,5	L	2 740,4	1 370,7	2 723,3	2 722,4	25
10	1 128,6	564,8	1 111,6	1 110,6	V	2 627,3	1 314,1	2 610,3	2 609,3	24
11	1 243,6	622,3	1 226,6	1 225,6	D	2 528,2	1 264,6	2 511,2	2 510,2	23
12	1 330,7	665,8	1 313,6	1 312,6	S	2 413,2	1 207,1	2 396,2	2 395,2	22
13	1 459,7	730,4	1 442,7	1 441,7	E	2 326,2	1 163,6	2 309,1	2 308,2	21
14	1 573,7	787,4	1 556,7	1 555,7	N	2 197,1	1 099,1	2 180,1	2 179,1	20
15	1 701,8	851,4	1 684,8	1 683,8	K	2 083,1	1 042,0	2 066,1	2 065,1	19
16	1 758,9	879,9	1 741,8	1 740,8	G	1 955,0	978,0	1 938,0	1 937,0	18
17	1 871,9	936,5	1 854,9	1 853,9	L	1 898,0	949,5	1 880,9	1 880,0	17
18	2 001,0	1 001,0	1 984,0	1 983,0	E	1 784,9	892,9	1 767,9	1 766,9	16
19	2 072,0	1 036,5	2 055,0	2 054,0	A	1 655,8	828,4	1 638,8	1 637,8	15
20	2 173,1	1 087,0	2 156,0	2 155,1	T	1 584,8	792,9	1 567,8	1 566,8	14
21	2 343,2	1 172,1	2 326,1	2 325,2	K+42	1 483,7	742,4	1 466,7	1 465,7	13
22	2 430,2	1 215,6	2 413,2	2 412,2	S	1 313,6	657,3	1 296,6	1 295,6	12
23	2 517,2	1 259,1	2 500,2	2 499,2	S	1 226,6	613,8	1 209,6	1 208,6	11
24	2 616,3	1 308,7	2 599,3	2 598,3	V	1 139,6	570,3	1 122,6	1 121,6	10
25	2 715,4	1 358,2	2 698,3	2 697,4	V	1 040,5	520,8	1 023,5	1 022,5	9
26	2 843,4	1 422,2	2 826,4	2 825,4	Q	941,4	471,2	924,4	923,4	8
27	2 980,5	1 490,7	2 963,5	2 962,5	H	813,4	407,2	796,4	795,4	7
28	3 079,6	1 540,3	3 062,5	3 061,5	V	676,3	338,7	659,3	658,3	6
29	3 166,6	1 583,8	3 149,6	3 148,6	S	577,3	289,1	560,2	559,2	5
30	3 295,6	1 648,3	3 278,6	3 277,6	E	490,2	245,6	473,2	472,2	4
31	3 424,7	1 712,8	3 407,6	3 406,7	E	361,2	181,1	344,2	343,2	3
32	3 481,7	1 741,4	3 464,7	3 463,7	G	232,1	116,6	215,1		2
33	3 655,8	1 828,4	3 638,8	3 637,8	R	175,1	88,1	158,1		1

#17 K401

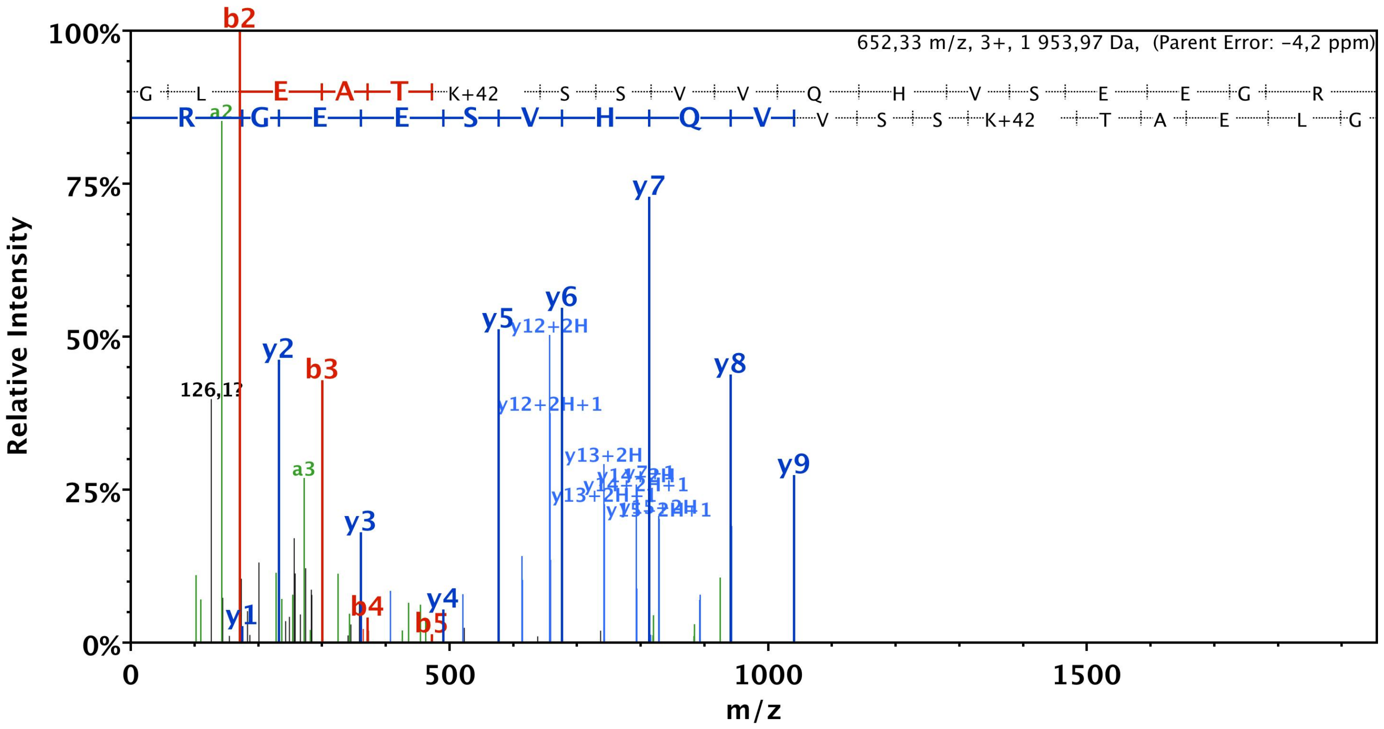

B	B Ions	B+2H	B-NH3	B-H2O	AA	Y Ions	Y+2H	Y-NH3	Y-H2O	Y
1	58,0	29,5			G	1 955,0	978,0	1 938,0	1 937,0	18
2	171,1	86,1			L	1 898,0	949,5	1 880,9	1 880,0	17
3	300,2	150,6		282,1	E	1 784,9	892,9	1 767,9	1 766,9	16
4	371,2	186,1		353,2	A	1 655,8	828,4	1 638,8	1 637,8	15
5	472,2	236,6		454,2	T	1 584,8	792,9	1 567,8	1 566,8	14
6	642,3	321,7	625,3	624,3	K+42	1 483,7	742,4	1 466,7	1 465,7	13
7	729,4	365,2	712,4	711,4	S	1 313,6	657,3	1 296,6	1 295,6	12
8	816,4	408,7	799,4	798,4	S	1 226,6	613,8	1 209,6	1 208,6	11
9	915,5	458,2	898,5	897,5	V	1 139,6	570,3	1 122,6	1 121,6	10
10	1 014,5	507,8	997,5	996,5	V	1 040,5	520,8	1 023,5	1 022,5	9
11	1 142,6	571,8	1 125,6	1 124,6	Q	941,4	471,2	924,4	923,4	8
12	1 279,7	640,3	1 262,6	1 261,7	H	813,4	407,2	796,4	795,4	7
13	1 378,7	689,9	1 361,7	1 360,7	V	676,3	338,7	659,3	658,3	6
14	1 465,8	733,4	1 448,7	1 447,8	S	577,3	289,1	560,2	559,2	5
15	1 594,8	797,9	1 577,8	1 576,8	E	490,2	245,6	473,2	472,2	4
16	1 723,8	862,4	1 706,8	1 705,8	E	361,2	181,1	344,2	343,2	3
17	1 780,9	890,9	1 763,8	1 762,9	G	232,1	116,6	215,1		2
18	1 955,0	978,0	1 938,0	1 937,0	R	175,1	88,1	158,1		1

#18 K401

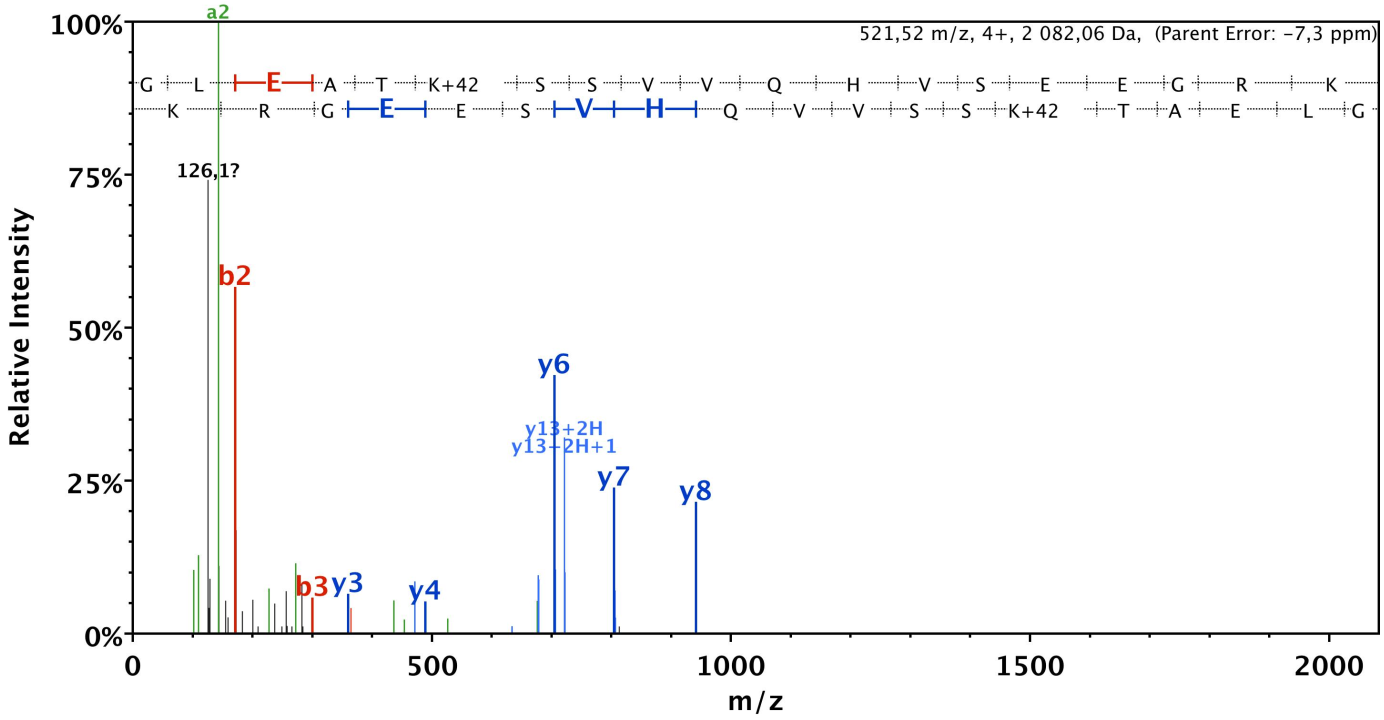

B	B Ions	B+2H	B-NH3	B-H2O	AA	Y Ions	Y+2H	Y-NH3	Y-H2O	Y
1	58,0	29,5			G	2 083,1	1 042,0	2 066,1	2 065,1	19
2	171,1	86,1			L	2 026,1	1 013,5	2 009,0	2 008,0	18
3	300,2	150,6		282,1	E	1 913,0	957,0	1 895,9	1 895,0	17
4	371,2	186,1		353,2	A	1 783,9	892,5	1 766,9	1 765,9	16
5	472,2	236,6		454,2	T	1 712,9	856,9	1 695,9	1 694,9	15
6	642,3	321,7	625,3	624,3	K+42	1 611,8	806,4	1 594,8	1 593,8	14
7	729,4	365,2	712,4	711,4	S	1 441,7	721,4	1 424,7	1 423,7	13
8	816,4	408,7	799,4	798,4	S	1 354,7	677,9	1 337,7	1 336,7	12
9	915,5	458,2	898,5	897,5	V	1 267,7	634,3	1 250,6	1 249,7	11
10	1 014,5	507,8	997,5	996,5	V	1 168,6	584,8	1 151,6	1 150,6	10
11	1 142,6	571,8	1 125,6	1 124,6	Q	1 069,5	535,3	1 052,5	1 051,5	9
12	1 279,7	640,3	1 262,6	1 261,7	H	941,5	471,2	924,5	923,5	8
13	1 378,7	689,9	1 361,7	1 360,7	V	804,4	402,7	787,4	786,4	7
14	1 465,8	733,4	1 448,7	1 447,8	S	705,4	353,2	688,3	687,3	6
15	1 594,8	797,9	1 577,8	1 576,8	E	618,3	309,7	601,3	600,3	5
16	1 723,8	862,4	1 706,8	1 705,8	E	489,3	245,1	472,3	471,3	4
17	1 780,9	890,9	1 763,8	1 762,9	G	360,2	180,6	343,2		3
18	1 937,0	969,0	1 919,9	1 919,0	R	303,2	152,1	286,2		2
19	2 083,1	1 042,0	2 066,1	2 065,1	K	147,1	74,1	130,1		1
