# Supplementary material for: CBP-HSF2 structural and functional interplay in Rubinstein-Taybi neurodevelopmental disorder
Source: Nat Commun. 2022 Nov 16;13:7002. doi: 10.1038/s41467-022-34476-2 (PMC9668993; doi:10.1038/s41467-022-34476-2)
Supplement: Supplementary file 5 — Reporting Summary [file 41467_2022_34476_MOESM5_ESM.pdf]

nature portfolio

Corresponding author(s): Aurélie de Thonel, Valerie Mezger

Last updated by author(s): 2022/10/12

Reporting Summary

Nature Portfolio wishes to improve the reproducibility of the work that we publish. This form provides structure for consistency and transparency in reporting. For further information on Nature Portfolio policies, see our Editorial Policies and the Editorial Policy Checklist.

Statistics

For all statistical analyses, confirm that the following items are present in the figure legend, table legend, main text, or Methods section.

n/a	Confirmed	
☐	☒	The exact sample size (*n*) for each experimental group/condition, given as a discrete number and unit of measurement
☐	☒	A statement on whether measurements were taken from distinct samples or whether the same sample was measured repeatedly
☐	☒	The statistical test(s) used AND whether they are one- or two-sided<br>*Only common tests should be described solely by name; describe more complex techniques in the Methods section.*
☒	☐	A description of all covariates tested
☒	☐	A description of any assumptions or corrections, such as tests of normality and adjustment for multiple comparisons
☐	☒	A full description of the statistical parameters including central tendency (e.g. means) or other basic estimates (e.g. regression coefficient) AND variation (e.g. standard deviation) or associated estimates of uncertainty (e.g. confidence intervals)
☐	☒	For null hypothesis testing, the test statistic (e.g. *F*, *t*, *r*) with confidence intervals, effect sizes, degrees of freedom and *P* value noted<br>*Give P values as exact values whenever suitable.*
☒	☐	For Bayesian analysis, information on the choice of priors and Markov chain Monte Carlo settings
☒	☐	For hierarchical and complex designs, identification of the appropriate level for tests and full reporting of outcomes
☒	☐	Estimates of effect sizes (e.g. Cohen's *d*, Pearson's *r*), indicating how they were calculated

Our web collection on statistics for biologists contains articles on many of the points above.

Software and code

Policy information about availability of computer code

Data collection

For mass spectrometry: Mascot (Matrix Science, Boston, MA; version 1.9.05) and X! Tandem (www.thegpm.org; version 2006.04.01.2) database 4 search algorithms; Peptide Prophet algorithm (7).
NB: the code is not available because this MS analysis was the seminal study for this paper and thus performed a long time ago. All the MS spectra are provided in Suppl. dataset 1. Note that the acetylation of the lysine residues that appeared relevant based on the MS study was nevertheless confirmed by independent methods (use of single or combined lysine mutations in immunoprecipitation experiments, in vitro acetylation coupled to RP-UFLC, rescue by 3KQ constructs etc...).

For modelisation of protein structure : we used web-servers and Open source programs. We didn't generate any code.
PsiPred : https://bioinfo3d.cs.tau.ac.il/FireDock/php.php
Blast : https://www.uniprot.org/blast
ClustalW : https://www.ebi.ac.uk/Tools/msa/clustalw2/).
Discovery Studio : version 18.1
Modeller (v9.19): downloaded according to the following protocol: https://salilab.org/modeller/tutorial/
SymmDock : http://bioinfo3d.cs.tau.ac.il/SymmDock/php.php
ERRAT (http://servicesn.mbi.ucla.edu/ERRAT/) , RESPROX (http://www.resprox.ca/) , Verify3D (http://servicesn.mbi.ucla.edu/Verify3D/)

Data analysis

- Immunofluorescence analysis were done with ImageJ (Fiji) software (ImageJ2 v2;3;0/1.53k)
- Statistical analysis were done with GraphPad Prism 8
- Modeling of the interaction between HSF2 and CBP domains : Zdock (v 2.1), Firedock, PyMOL (v2.0)
- Mass spectrometry analysis: Scaffold proteome software (1.0) was used for the visualization of the MS data. All the MS spectra are provided in Suppl. dataset 1.

Biolayer interferometry : ForteBio, Data analysis software version 7.1.0.89.

Analysis of SNP arrays : GenomeStudio software (v2.05)

For manuscripts utilizing custom algorithms or software that are central to the research but not yet described in published literature, software must be made available to editors and reviewers. We strongly encourage code deposition in a community repository (e.g. GitHub). See the Nature Portfolio guidelines for submitting code & software for further information.

Data

Policy information about availability of data

All manuscripts must include a data availability statement. This statement should provide the following information, where applicable:

- Accession codes, unique identifiers, or web links for publicly available datasets
- A description of any restrictions on data availability
- For clinical datasets or third party data, please ensure that the statement adheres to our policy

All the data generated during this study are available from the corresponding authors on reasonable request. No code was generated for this study.

Field-specific reporting

Please select the one below that is the best fit for your research. If you are not sure, read the appropriate sections before making your selection.

☒ Life sciences ☐ Behavioural & social sciences ☐ Ecological, evolutionary & environmental sciences

For a reference copy of the document with all sections, see nature.com/documents/nr-reporting-summary-flat.pdf

Life sciences study design

All studies must disclose on these points even when the disclosure is negative.

Sample size	No sample-size calculations were performed, rather, at least three individual samples (cell pellets, pools of embryonic cortices or of organoids, see below) were used in each independent experiment, based on our previous work (DOI 10.15252/emmm.201303311) and the literature (https://www.nature.com/articles/s41586-019-1289-x).<br>For WB analysis on organoids and mouse cortices : 2-3 organoids/ 4-5 embryonic cortices of each genotype per experiment (around 20µg of protein extract per sample).<br>For immunofluorescence experiments on cells: 100-200 cells per experiment.<br>For immunofluorescence experiments comparing HD and RSTS CBP organoids : for HD, n = 3 hCOs, 15 loops, 142 mitoses; for RSTSCBP, n = 4 hCOs, 18 loops.<br>For immunofluorescence experiments comparing HD and RSTS EP300 organoids : for HD, n = 11 hCOs, 52 loops, 526 mitoses; for RSTS, n = 8 hCOs, 43 loops.
Data exclusions	On principle, data were only excluded for technically failed experiments (failure of antibody staining in WB or IF (around 5% of the experiments); failure in quality control in hCO production (exacerbated generation of choroid plexus (around 3% in RSTS EP300 and 10% in CBP hCOs).
Replication	Experiments were replicated multiple times with reproducible results, as indicated in the figure legends.
Randomization	Randomization was not performed because we used a batch of cells or organoids that are paired (e.g. control versus treated cells, wild-type versus mutant cortices, or organoids patients - versus heathy donors). Organoids used for analyses within each experimental group, were chosen from each batch to be representative of the morphology seen at this stage of differentiation (DIV).
Blinding	Investigators were not blinded. For phenotyping of organoids, the quantification of ectopic mitoses in organoids from healthy donors and RSTS patients was performed by three researchers under blinded conditions.

Reporting for specific materials, systems and methods

We require information from authors about some types of materials, experimental systems and methods used in many studies. Here, indicate whether each material, system or method listed is relevant to your study. If you are not sure if a list item applies to your research, read the appropriate section before selecting a response.

Materials & experimental systems

n/a	Involved in the study
[ ]	[x] Antibodies
[ ]	[x] Eukaryotic cell lines
[x]	[ ] Palaeontology and archaeology
[ ]	[x] Animals and other organisms
[ ]	[x] Human research participants
[x]	[ ] Clinical data
[x]	[ ] Dual use research of concern

Methods

n/a	Involved in the study
[x]	[ ] ChIP-seq
[x]	[ ] Flow cytometry
[x]	[ ] MRI-based neuroimaging

Antibodies

Antibodies used

The table below, describing the list of all antibodies used, its dilutions, provider, validation and catalogue number, is also included in the "Supplementary Methods" section of our manuscript (see the "Supplementary Information" file)

This list contains: Antibodies species; Clone reference (mouse); Manufacturer; dilutions for WBs; for IPs, IFs; Validation or RRID
Acetyl-lysine (Pan) rabbit pAb #9441 Cell signalling Technology WB : 1/1000 IF: 1/1000 RRID: AB_331805
Actin mouse AC40 A3853 Sigma-Aldrich WB : 1/4000 RRID:AB_262137
Alexa Fluor 488 mouse 715-546-151 J.ImmunoRes IF:1/800
Alexa Fluor 488 rabbit A-11008 J.ImmunoRes IF:1/800
Alexa Fluor 594 rabbit A-11037 J.ImmunoRes IF:1/800
CBP rabbit IgG D6C5 #7389 Cell signalling Technology WB : 1/1000 IF:1/100 RRID: AB_2616020
CBP rabbit A-22 sc-369 Santa-Cruz WB : 1/1000 RRID:AB_631006
Cy3TM-3 mouse 715-165-150 J.ImmunoRes IF:1/800
EP300/CBP Mouse pAb sc-32244 Santa-Cruz IF:1/25 RRID:AB_628076
EP300 rabbit pAb sc-584 Santa-Cruz WB : 1/500 IF:1/200 RRID:AB_2293429
FABP7/BLBP mouse AT1D1 AM09059PU-S Origen IF:1/400 RRID:AB_1652805
Flag tag mouse M2 F1804 Sigma-Aldrich WB : 1/1000 IP: 2µg RRID:AB_262044
GFP tag mouse IgG1 MAB2510 Millipore WB : 1/1000 RRID:AB_94623
GFP-Trap-A mouse gta-20 chromotek IP: 25µL RRID:AB_263135
GST tag mouse IgG AE001 Ab Clonal WB : 1/2000 IP: 2.5µL RRID:AB_2770403
H3K18Ac rabbit pAb GTX128943-S Euromedex WB: 1/2000 RRID: AB_2885843
H3K27Ac rabbit pAb C15410174 Diagenode WB : 1/1000 RRID:AB_2716835
HA tag mouse 16B12 MMS101R Covance WB : 1/2000 IF:36526 RRID:AB_291262
HDAC1 rabbit pAb ab7028 Abcam WB : 1/4000 IF:1/900 RRID:AB_305705
HRP mouse mouse 115 035 135 J.ImmunoRes WB : 1/50 000
HRP mouse Fab mouse F(ab')2 115 036 072 J.ImmunoRes WB : 1/50 000
HRP rabbit rabbit IgG1 211 032 171 J.ImmunoRes WB : 1/50 000
HSC70 rat mAb ADI-SPA-815 Stressgen WB : 1/1000 RRID:AB_10617277
HSF1 rabbit pAb #4356 Cell signalling Technology WB : 1/1000 IF:1/800 RRID:AB_2861388
HSF2 mouse G11 sc-74529 Santa-Cruz WB : 1/250 IP: 4µg validation: Hsf2-/- mouse line (see Supplementary Fig. 8b).
HSF2 mouse 3E2 ab69621 abcam IP: 4µg; validation: El Fatimy et al. (2014)
HSF2 rabbit pAb SFI57 Lea Sistonen Lab (Ostling et al., 2007) IF:1/600; validation: Ostling et al. (2007)
HSP70 mouse C92F3A-5 ADI-SPA-810 Stressgen WB: 1/1000 RRID: AB_2039260
HSP90 mouse H9010 SMC-107 Stressmarq WB : 1/3000 RRID: AB_2697870
HSP110 mouse 58F12 NCL-HSP105 Leica Biosystem WB : 1/10 000 RRID: AB_563775
HuC/D mouse 16A11 A21271 Thermoscientific IF:1/500 RRID: AB_221448
IgG mouse I5381 sigma-Aldrich
Myc tag mouse 9B11 #2276 Cell signalling Technology WB : 1/1000 RRID: AB_331783
Myc-Trap-A mouse yta-20 chromotek IP: 25µL RRID: AB_2631369
Nanog rabbit pAb 14295-1-AP Proteintech IF:1/200 RRID: AB_1607719
Nde1 rabbit 10233-1-AP Proteintech WB : 1/1500 RRID: AB_2149877
Nestin mouse 10C2 MA1-110 Invitrogen IF:1/200 RRID: AB_2536821
N-cadherin rabbit 22018-1-AP Proteintech WB : 1/2000 RRID: AB_2813891
N-cadherin mouse GC4 C2542-100UL Sigma IF:1/1000 RRID: AB_258801
Oct-3/4 rabbit EPR17929 Ab181557 Abcam IF:1/200 RRID: AB_2687916
Pax 6 rabbit pAb 12323-1-AP Proteintech IF:1/500 RRID: AB_2159695
PCNA rabbit PA5 #272-14 Thermoscientific WB : 1/1000 RRID: AB_2544690
Phospho-Histone H3 rat HTA28 ab10543 Abcam IF:1/200 RRID: AB_2295065
Snap rabbit pAb P9310 NEB WB : 1/1000 RRID: AB_10631145
Sox 2 rabbit pAb ab97959 Abcam IF:1/200 RRID: AB_2341193
Trap-A CTL bab-20 Chromotek IP: 25µL RRID: AB_2827547
Anti-TJP1/ZO-1 mouse 1A12 33-9100 FisherScientifique IF:1/1000 RRID: AB_2533147
TBR1 coralite 594 mouse IgG1-k CL594-66564 Proteintech IF:1/500 RRID: AB_2529847

	Tuj-1 mouse 2G10 T8578 sigma-Aldrich IF:1/1000 RRID: AB_1841228<br>Ubiquitin mouse FK2 BML-PW8810-0100 Enzolifes Sciences WB : 1/500 RRID: AB_10541840
Validation	- Most of the antibodies were commercially validated (RRID, Table for antibodies).<br>- HSF2 antibody (G11; Santa Cruz) sc-74529 was validated using in house - Hsf2 -/- mouse cortex extracts (Supplementary Fig. 7b.).<br>- HSF2 mouse 3E2 ab69621 abcam IP: 4µg; validation: El Fatimy et al. EMBO Mol Med (2014)<br>- HSF2 antibody produced by the Lea Sistonen's Lab was validated previously (SFI57, Ostling et al., 2007 Journal of Biological Chemistry)

Eukaryotic cell lines

Policy information about cell lines

Cell line source(s)

For RSTS patients P1 to P5, biopsies were performed in childhood, preadolescence or young adulthood, and the ratio of the number of males to the number of females was 3 to 2.
I- For the derivation of human iPSC lines from RSTS patients 1 and 2.
Patient RSTS P1 (CREBBP); Patient RSTS P2 (EP300).
I.1 Skin biopsies: Written informed consents for skin biopsies were obtained from the patients' legal guardians by Dr Sandrine Passemard (co-author) at Hôpital Robert Debré (Paris, Assistance Publique – Hôpitaux de Paris (AP-HP)). The corresponding research projects were approved by the National Ethics Committee (Comité de Protection des Personnes (CPP) Ile-de-France II, number 2010AO1481-38).

I.2 Primary skin fibroblasts from Patient RSTS P1 and P2 biopsies: The RSTS primary skin fibroblasts were derived from these biopsies and belong to a collection of skin fibroblasts for in vitro culture, derived from patients with rare hereditary diseases » (agreement n° P100128; « Fibroblastes en culture issus de peau de patients atteints de maladies héréditaires rares »), headed by Dr. Élise Lebigot (co-author) and located at the Centre Hospitalo-Universitaire Bicêtre (AP-HP). The derivation of primary skin fibroblasts and the storage of the collection was approved and registered by the Département de la Recherche Clinique et du Développement (DRCD), Groupement Inter-régional de Recherche Clinique et d'Innovation d'Ile de France) (AP-HP), through consent for use for research (DC 2009-939).

I.3 The derivation of iPSCs from RSTS P1 and RSTS P2 Primary skin fibroblasts: the « Cellule de bioéthique, Direction générale de la recherche et de l'innovation » at the French Ministère de l'enseignement supérieur et de la Recherche (MESRI) delivered the CODECOH agreement (DC-2021-4446) that validated the derivation at the iPSC core facility of Nantes, and banking, storage and use of these iPSC lines at the Epigenetic and Cell Fate Center

II- Purchased commercial healthy donor iPSCs
IMR90-4: WiCell, USA; MTA 21-W0506 (female; fetal). The CODECOH agreement DC-2021-4446) by the above-cited the « Cellule de bioéthique" also approved the use of these commercial iPSCs form WiCell.

III- For ESCs
Human embryonic stem cell H1 and H9 anonymous cell lines are commercially available cell lines (WiCell; https://hpscreg.eu/cell-line/WAe001-A; https://hpscreg.eu/cell-line/WAe009-A:).
III.1 The use of Human ESC H9 (female) to generate cerebral organoids at the Lancaster Lab was approved by the UK Stem Cell Board.
III.2 Human ESC lines (H1 male and H9 female cells) were used by the iPSC core facility of Nantes for producing RNA lysates that were used as positive controls for the characterization of iPSCs derived from RSTS patients P1 and P2. These hESC lines have been imported, banked and used under the agreement of Agence de la Biomédecine RE17_007.

IV- For the derivation of lymphoblastoid cell lines
IV.1 For the derivation of the lymphoblastoid cell line coming from a healthy donor: the lymphoblastoid cell line LLD 138 is a kind gift from Prof. Evani-Viegas Pequignot (Institut Jacques Monod, Université Paris Diderot (now UniversitéParis Cité), a founder of our Epigenetics and Cell Fate Center. LLD 138 has been described in Almeida et al. (1993). At this time, patient 's consent was not necessary to derive this cell line. For this reason, there is no information about the sex, nor the age of the donor.

IV.2 For the derivation of lymphoblastoid cells from RSTS patients (RSTS patients 3 to 5)
- For patient RSTS P3 (EP300) and patient RSTS P5 (CREBBP): these RSTS lymphoblastoid cell lines belong to the collection that was registered at the creation of the « Centre de Ressources Biologique"s CRB-BioJeL » and have the authorization to transfer material for scientific use, after approval by « Cellule de bioéthique, Direction générale de la recherche et de l'innovation » at the French Ministère de l'enseignement supérieur et de la Recherche (MESRI) delivered the CODECOH agreement (DC-2009-1044 and AC-2015-2579) which implies informed consent for the derivation of lymphoblastoid cell lines. The MTA for the use of these cell lines in the context of this study was given by CRB-BioJeL to Epigenetic and Cell Fate center (BB-0033-00016; May 05, 2018, Paris).

- For Patient RSTS P4 (CREBBP). The corresponding RSTS lymphoblastoid cell line, provided by Isabelle COUPRY and Benoit ARVEILER, belongs to the « Génétique-Maladies Rares » (Genetics – Rare diseases) collection, which was registered at the creation of the « Bordeaux Centre de Ressources Biologique » (CRB), after its approval by the "Comité de Protection des Personnes du Sud-Ouest Outre-Mer III" (DC-2014-2164), on the basis of informed consent for the derivation of lymphoblastoid cell lines.

NB1: As we transferred all the ethical parts concerning human material and mouse models to the paragraph called "Ethical regulation", we deleted the corresponded sentence for the paragraph now called "Cell and cell line culture" and "Mouse

	model" (yellow and strikethrough text).<br>NB2: In addition, we noticed that we had made an error in the previous version of the Methods section that is now deleted (page 15, Lane 590-592; notably, this does not modify our data, findings, nor their interpretation): Patient 3-derived lymphoblastoid cells are from CRB-Institut Médical Jérôme Lejeune (CRB-BioJeL, Paris) and not from CHU Bordeaux, and Patient 4 is from CHU Bordeaux. We apologize for that. This emphasizes the importance of such editorial process, thank you for that! The corrections were made in the new version integrated to the Reporting Summary and the Methods.<br><br>V- Primary skin fibroblasts from healthy donors<br>HD1, HD2 (8-day-old males). These primary skin fibroblast cells are described in Yehezkel et al. (2008). They come from anonymous gifts of foreskins, removed during circumcision, for which at the time the study by Yehezkel et al. was done, there was no legal consent to be given. No cell line was derived from these fibroblasts.<br><br>- Commercially available cell line used: Neuro2A (DSMZ # ACC 148) , HEK cells (ATCC®, CRL-11268TM) U20S (osteosarcoma, ATCC®, HTB-96TM), SH-SY5Y (neuroblastoma, ATCC® CRL-2266TM); IMR90-4 (healthy donor iPSC line; WiCell®, 21-W0506).<br>- Hamster BHK cells were kindly provided by Dr. Leonhardt H and cultured as described in Herce et al., (2013, Nat. Commun) and not further authenticated.
Authentication	- Cell lines were authenticated by the commercial supplier and not further authenticated in this study.<br>- RSTS iPSC cell lines were authenticated by the Nantes iSPC Plateform :<br>> Genotype verification of patient-derived iPSCs<br>PCR PRIMERS<br>hEP300-in7-5p : CTGTTCTTGAACTCCTGACC<br>hEP300-ex12-3p: TTGAGAGCTCCAGGTTGAGC<br>hCREBBP_ex18_F: AGAATCCCATGGACCTCTCC<br>hCREBBP_ex18_R: ATCCAAGGGACTGCATGACA<br><br>> RT-qPCR:<br>PCR PRIMERS<br>Gene Name Primer sequence 5'-3' Amplicon size (bp) Melting Temp. (°C) Position<br>GAPDH AATCCCATCACCATCTTCCA<br>TGGACTCCACGACGTACTCA 82 80.5 494-576<br>OCT4 TGGGTGGAGGAAGCTGACAACAAT<br>TTCGGGCACTGCAGGAACAAATTC 142 82.1 1005-1147<br>SOX2 CCTACTCGCAGCAGGGCACC<br>CTCGGCGCCGGGGAGATACA 169 78 1114-1283<br>NANOG ATAGCAATGGTGTGACGCAGAAGG<br>CTGGTTGCTCCACATTGGAAGGTT 116 82 701-816<br><br>> Early germ layer differentiation<br>> Expression profiling by 3'SRP<br>> SNP analysis, (See supplementary Figure 9)<br><br>- iNPC were authenticated by immunofluorescence, using specific pluripotency and neural makers (PAX6; FABP7; OCT3/4; See Supplementary Figure 10)
Mycoplasma contamination	Cell lines were routinely tested for mycoplasma contamination by PCR and confirmed that they were negative for mycoplasma contamination
Commonly misidentified lines<br>(See ICLAC register)	The cells we used are not part of this database

Animals and other organisms

Policy information about studies involving animals; ARRIVE guidelines recommended for reporting animal research

Laboratory animals	All mice were hosted in pathogen-free conditions (area overpressurized), with ad libitum access to rodent laboratory food and water in an ambient temperature set at 21°C+/-1, a day/night cycle is 12/12h, a humidity set between 40 and 70% in accordance to the guidelines of the French Ministère de l'Enseignement Supérieur, de la Recherche et de l'Innovation. All mice stains used in this study are described in "Methods" section of the manuscript. C57BL/N female mice and C57Bl/6N Hsf2-/- male/female mice were used for the experiments, median age (8 weeks +/- 2 weeks).
Wild animals	no wild animals were used in the study.
Field-collected samples	no field-collected samples were used in the study
Ethics oversight	Animal Experimentation Ethical Committee Buffon (CEEA-40) and recorded under the following reference by the Ministère de l'Enseignement Supérieur, de la Recherche et de l'Innovation (#2016040414515579).

Note that full information on the approval of the study protocol must also be provided in the manuscript.

Human research participants

Policy information about studies involving human research participants

Population characteristics	Human embryonic stem cell H1 and H9 anonymous cell lines are commercially available cell lines (WiCell; https://hpscreg.eu/cell-line/WAe001-A; https://hpscreg.eu/cell-line/WAe009-A:). The work was performed on cells from patients (primary skin fibroblasts, lymphoblastoid and induced pluripotent stem cells), Patient characteristics and related ethical aspects are described in the "Eukaryotic cell lines" section.
Recruitment	The work was performed on cells from patients (primary skin fibroblasts, lymphoblastoid and induced pluripotent stem cells), Patient characteristics and related ethical aspects are described in the "Eukaryotic cell lines" section.
Ethics oversight	The work was performed on cells from patients (primary skin fibroblasts, lymphoblastoid and induced pluripotent stem cells), Patient characteristics and related ethical aspects are described in the "Eukaryotic cell lines" section.

Note that full information on the approval of the study protocol must also be provided in the manuscript.
